# General continuous-time Markov model of sequence evolution via insertions/deletions: are alignment probabilities factorable?

**DOI:** 10.1186/s12859-016-1105-7

**Published:** 2016-08-11

**Authors:** Kiyoshi Ezawa

**Affiliations:** 1 Department of Bioscience and Bioinformatics, Kyushu Institute of Technology, Iizuka, 820-8502 Japan; 2 Department of Biology and Biochemistry, University of Houston, Houston, TX 77204-5001 USA

**Keywords:** Stochastic evolutionary model, Insertion/deletion (indel), Sequence alignment probability, Factorability, Biological realism, Power-law distribution, Rate variation, Non-equilibrium evolution

## Abstract

**Background:**

Insertions and deletions (indels) account for more nucleotide differences between two related DNA sequences than substitutions do, and thus it is imperative to develop a stochastic evolutionary model that enables us to reliably calculate the probability of the sequence evolution through indel processes. Recently, indel probabilistic models are mostly based on either hidden Markov models (HMMs) or transducer theories, both of which give the indel component of the probability of a given sequence alignment as a product of either probabilities of column-to-column transitions or block-wise contributions along the alignment. However, it is not *a priori* clear how these models are related with any genuine stochastic evolutionary model, which describes the stochastic evolution of an *entire* sequence along the time-axis. Moreover, currently none of these models can fully accommodate biologically realistic features, such as overlapping indels, power-law indel-length distributions, and indel rate variation across regions.

**Results:**

Here, we theoretically dissect the *ab initio* calculation of the probability of a given sequence alignment under a genuine stochastic evolutionary model, more specifically, a general continuous-time Markov model of the evolution of an entire sequence via insertions and deletions. Our model is a simple extension of the general “substitution/insertion/deletion (SID) model”. Using the operator representation of indels and the technique of time-dependent perturbation theory, we express the *ab initio* probability as a summation over all alignment-consistent indel histories. Exploiting the equivalence relations between different indel histories, we find a “sufficient and nearly necessary” set of conditions under which the probability can be factorized into the product of an overall factor and the contributions from regions separated by gapless columns of the alignment, thus providing a sort of generalized HMM. The conditions distinguish evolutionary models with factorable alignment probabilities from those without ones. The former category includes the “long indel” model (a space-homogeneous SID model) and the model used by Dawg, a genuine sequence evolution simulator.

**Conclusions:**

With intuitive clarity and mathematical preciseness, our theoretical formulation will help further advance the *ab initio* calculation of alignment probabilities under biologically realistic models of sequence evolution via indels.

**Electronic supplementary material:**

The online version of this article (doi:10.1186/s12859-016-1105-7) contains supplementary material, which is available to authorized users.

## Background

The evolution of DNA, RNA and protein sequences is driven by mutations such as base substitutions, insertions and deletions (indels), recombination and other genomic rearrangements (*e.g*., [1–3]). Some recent comparative genomic analyses revealed that indels account for more base differences between closely related genomes than base substitutions do (*e.g*., [4–7]). It is therefore imperative to develop a method to reliably calculate the probability of sequence evolution via mutations including indels. Since the groundbreaking works by Bishop and Thompson [8] and by Thorne, Kishino and Felsenstein [9], many studies have been made to calculate the probabilities of pairwise alignments (PWAs) and multiple sequence alignments (MSAs) under probabilistic models aiming to incorporate the effects of indels. And the methods have greatly improved in terms of the computational efficiency and the scope of application (reviewed, *e.g*., in [10–12]). Most of these studies are based on hidden Markov models (HMMs) (*e.g*., [13]) or transducer theories (*e.g*., [14]). Even today, the studies on these methods are steadily advancing (*e.g.*, [15, 16]), and it seems that their mathematical and algorithmic bases are about to be established.

However, it is important to remember that these desirable properties *alone* are not sufficient for a model in natural science to be satisfactory. In addition to having the mathematical (or algorithmic) soundness, a satisfactory model must also approximate well, or at least decently, the real phenomena it is intended to describe. In the case of an indel probabilistic model, there are two key elements for this requisite: one is the evolutionary consistency of the model, and the other is the model’s flexibility to accommodate various biologically realistic features, *i.e.*, the biological realism, of indels.

**Fig. 1:**
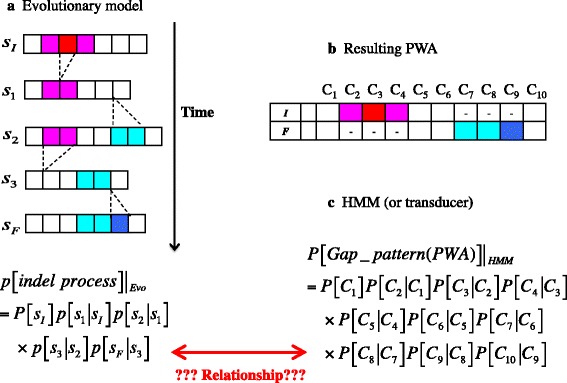
Genuine stochastic evolutionary model vs. HMM (or transducer). **a** Probability density calculation via a genuine stochastic evolutionary model. Each sequence state is represented as an array of sites (boxes). Sites to be deleted are shaded in red or magenta. Inserted sites are shaded in blue or cyan. The *s*_*I*_ and *s*_*F*_, respectively, denote the initial and final states. The *s*_*ν*_ (*ν* = 1, 2, 3) is an intermediate state. (The “*P*[…]” denotes a probability, the “*p*[…]” denotes a probability density.) **b** A pairwise alignment (PWA) between the initial (*I*) and final (*F*) states resulted from the indel process in panel **a**. The *C*_*i*_ (*i* = 1, …, 10) labels the alignment column below. **c** Probability calculation via a HMM (or a transducer). It is a priori unclear whether or how the methods in panels **a** and **c** are related with each other. For clarity, residue states and substitutions were omitted. (Note that the equation in panel **a** is merely a rough expression to give a broad idea on the issue. Rigorous expressions will be given in *Results and discussion*.) Panels **a** and **b** of this figure were adapted from panels B and F of Fig. 1 of [32]

Let us first explain the evolutionary consistency. In natural sequence evolution, the probability (density) of an indel process must be given *vertically*, as a multiplicative accumulation of the probabilities of transitions between states of an *entire* sequence, each from one time point to the next one, along the time axis (or along a phylogenetic tree when dealing with MSAs) (Fig. 1a). If a probabilistic model gives the probability density of each evolutionary process according to this natural design, we call it a “genuine stochastic evolutionary model”, or simply an “evolutionary model” for short. And we will consider an indel probabilistic model as “evolutionarily consistent”, if its alignment probabilities can be derived directly from an evolutionary model, even if it does not appear to follow the aforementioned natural design. By definition, each of HMMs and transducers calculates (the indel component of) an alignment probability *horizontally*, as a product of either inter-column transition probabilities or block-wise contributions (Fig. 1c). Therefore, it is *a priori* unclear whether each HMM or transducer is evolutionarily consistent or not, or, if it is, how (Fig. 1). It would be worth mentioning that some models were indeed derived explicitly from some sorts of evolutionary models (*e.g*., [9, 17, 18]). Unfortunately, all such studies in the past imposed some unnatural assumptions, such as the prohibition of overlapping indels and the restriction of deletions to single-base ones. Such assumptions were necessary for an alignment probability to be trivially factorable, at least as a product of block-wise contributions. Thus, they are unsatisfactory from the viewpoint of the second key element, *i.e*., the biological realism.

Regarding the biological realism, past empirical studies revealed some properties of real indels, in addition to their possibilities to affect multiple contiguous residues at a time and to overlap others. Among the most important would be the studies that showed power-law distributions of indel lengths (see, *e.g*., [19] and references therein). On the contrary, standard HMMs and transducers can usually implement geometric distributions of indel lengths, or at best mixed geometric distributions (*e.g*., [20]), but cannot implement the power-law distributions themselves. But some generalized HMMs (or transducers) (*e.g*., [21, 22]) can incorporate power-law indel length distributions. For example, the HMM of Kim and Sinha [22] is quite flexible, and it can incorporate the power-law distributions and also do away with the commonly imposed time-reversibility. As discussed *e.g*., in [21] and [23], there is no biological reason for imposing the time reversibility, and they were usually imposed to reduce the computational time. In this sense, the HMM of Kim and Sinha is two steps closer to the biological reality than the standard HMMs (and transducers). Unfortunately, similarly to the standard HMMs and transducers, their HMM is not evolutionarily consistent and thus cannot correctly handle overlapping indels along the same branch, though they can handle overlapping indels along different branches. Another possibly important biologically realistic feature is the indel rate variation across sites (or regions) (*e.g*., [24]), due to selection and the mutational predispositions (caused, *e.g.*, by the sequence or epigenetic contexts). Thus far, attempts to incorporate this feature have been rare (*e.g*., [25]), and most studies have handled space-homogeneous models, whose indel rates are homogeneous along the sequence.

As far as we know, except the models implemented in some genuine sequence evolution simulators (*e.g*., [26–28]), there is only one class of genuine stochastic evolutionary models discussed thus far that is also considerably biologically realistic, which are the “substitution/insertion/deletion (SID) models” proposed by Miklós et al. [21]. The SID models in general do not impose the aforementioned unnatural restrictions on indels. Moreover, the general SID model can accommodate any indel length distributions, and also some indel rate variations across sites (albeit through the residue state context alone). Unfortunately, however, we have not seen any further theoretical development of the general SID model since it was proposed. Instead, Miklós et al. developed the “long indel” model [21], which is a space-homogeneous, time-reversible SID model. (More precisely, the insertion rate depends on the inserted sequence only through the product of the frequencies of its constituent residues. As mentioned above, the time-reversibility was introduced just for computational convenience, and it could be dispensed with if desired.) And they gave a verbal justification that the probability of a PWA under the “long indel” model can be calculated via a generalized HMM, as a product of contributions from “chop-zones” delimited by gapless columns. In the present viewpoint, this is the most satisfactory HMM that we know was used for actual sequence analyses, because it satisfies both the evolutionary consistency and the biological realism to some degree. Nevertheless, their justification, although plausible, has two problems. First, it is unclear exactly how their HMM is related to the *ab initio* probabilities of evolutionary (especially indel) processes under their evolutionary model. And second, their justification takes advantage of the space-homogeneity of the model, which makes it unclear how their HMM can be extended to be space-heterogeneous while keeping the evolutionary consistency. To solve these two problems, we need at least to get back to their origin, *i.e.*, the general SID model. It seems, however, that this model has never been theoretically dissected thus far, possibly due to the lack of mathematical or conceptual tools to handle it easily.

In this study, we examine a general continuous-time Markov model of the evolution of an *entire* sequence via insertions, deletions and substitutions. This model could be regarded as an extension of the general SID model, in the sense that it allows explicit (or inherent) rate variation across sites, not only due to residue state contexts. Such rate variation could be regarded as the effect of, *e.g*., the epigenetic context and/or the context within the 3D structure of the protein product (*e.g*., [25]).^1^ To theoretically dissect the *ab initio* calculation of alignment probabilities under this model, we introduce some useful tools. Among the most important would be the operator representation of mutations, namely, insertions, deletions and substitutions. This enabled us to shift our focus from the trajectory of sequence states, which played a central role in [21], to the history of mutations (especially indels). Moreover, the operator representation enabled to algebraically define the equivalence relationships between two different series of indels. They, in cooperation with the focus shift, enabled to define the local history set (LHS) equivalence classes. These equivalence classes play an essential role when deriving the “sufficient and nearly necessary” set of conditions under which alignment probabilities are indeed factorable, thus providing a sort of generalized HMM. We also adapt techniques from the time-dependent perturbation expansion in quantum mechanics [29, 30], expanding each alignment probability into a summation over contributing mutational histories with different numbers of indels. It should be noted, however, that we formally deal with all terms in the expansion.^2^ Thus, at least formally, the probabilities we deal with are exact solutions of the model’s defining equation. For clarity, we will focus on insertions/deletions in the bulk of the manuscript. However, we can also incorporate substitutions; see, *e.g*., [31] for more details.

This paper describes the backbone of our study (more extensively recorded in an unpublished paper [32]) to give the theoretical basis of our *ab initio* probability calculation under the general continuous-time Markov model of indels. Peripheral topics surrounding the study can be found in [32].^3^ Throughout the paper, we suppose that each probability is calculated under a given evolutionary model setting, including the phylogenetic tree of the sequences. In section R1 of Results and discussion, we briefly review the most general form of the SID model [21]. Then, in section R2, we introduce two important tools, namely, the ancestry index and the operator representation of mutations including indels. Using the results of sections R1 and R2, we define our general continuous-time Markov model in section R3, and formally give the general solution to its defining equation in terms of the operator representation. In section R4, we formally express the *ab initio* probability of a given PWA in a perturbation expansion. Then, using the concept of the LHS equivalence classes defined in section R5, we derive in section R6 the conditions under which the PWA probability is factorable. In section R7, the derivation is extended to the probability of a given MSA. In section S8, some examples are given to illustrate models with factorable and non-factorable alignment probabilities. The former category includes the indel evolutionary model of Dawg [26] and the “long indel” model [21], among others. In section R9, we discuss the merits, possible uses and extensions, as well as some outstanding issues, of the results in this study. In Table 1, we summarize the key concepts and results of this paper, mainly for those who want its gist quickly. Likewise, Table S1 (in Additional file 1) summarizes mathematical symbols used commonly in this paper, to facilitate the readers’ cruise through the equations. Supplementary methods (in Additional file 1) and Supplementary appendix (in Additional file 2) give detailed derivations of some important results. The former is more essential and accessible to a wider audience; the latter is for those who are interested in further mathematical details.

**Table 1:** Key concepts and results in this paper

Concept/result	Description	Main location
**Ancestry index**	An ancestry index is assigned to each site. Sharing of an ancestry index among sites indicates the sites’ mutual homology. As a fringe benefit, the indices enable the mutation rates to vary across regions (or sites) beyond the mere dependence on the residue state of the sequence.	Section R2 (1st and 2nd paragraphs), Fig. 2
**Operator representation of mutations**	This enables the intuitively clear and yet mathematically precise description of mutations, especially insertions/deletions, on sequence states. This is a core tool in our *ab initio* theoretical formulation of the genuine stochastic evolutionary model.	Section R2 (3rd paragraph), **Fig.** **3**
Rate operator	An operator version of the rate matrix, which specifies the rates of the instantaneous transitions between the states in our evolutionary model. In other words, the rate operator describes the instantaneous stochastic effects of single mutations on a given sequence state.	Section R3, Eqs. (R3.1-R3.9) (full mutational model), **Eqs. (** **R3.2**,**R3.6**,**R3.11**-**R3.15** **)** (indel model)
Finite-time transition operator	An operator version of the finite-time transition matrix, each element of which gives the probability of transition from a state to another after a finite time-lapse. This results from the cumulative effects of the rate operator during a finite time-interval.	Section R3, **Eq. (** **R3.17** **)**, Eq. (R3.18)
Defining equations (differential)	1st-order time differential equations (forward and backward) that define our indel evolutionary model. They are operator versions of the standard defining equations of a continuous-time Markov model.	Section R3, Eqs. (R3.19,R3.21) (forward), Eqs. (R3.20,R3.21) (backward)
**Defining equations (integral)**	Two integral equations (forward and backward) that are equivalent to the aforementioned differential equations defining our indel evolutionary model. They play an essential role when deriving the perturbation expansion of the finite-time transition operator.	Section R4, **Eq. (** R4.4 **)** (forward), **Eq. (** **R4.5** **)** (backward)
**Perturbation expansion (transition operator)**	The perturbation expansion of the finite-time transition operator. It was derived in an intuitively clear yet mathematically precise manner, by using the aforementioned defining integral equations.	Section R4, **Eqs. (** **R4.6**,**R4.7** **)**
Perturbation expansion (*ab initio* PWA probability)	The perturbation expansion of the *ab initio* probability of a given PWA, conditioned on the ancestral sequence state, under a given model setting.	Section R4, Eq. (R4.8) or Eq. (R4.9)
**Binary equivalence relation**	An equivalence relation between the products of two indel operators each. The relations play key roles when defining LHS equivalence classes.	Section R5, **Eqs. (** **R5.2a**-**R5.2d** **)**
**Local-history-set (LHS) equivalence class**	An equivalence class consisting of global indel histories that share all local history components. The classes play an essential role when proving the factorability of a given PWA probability.	Section R5, **below Eq. (** **R5.4** **)**, (*e.g*., Fig. 5)
**Factorability (** ***ab initio*** **PWA probability)**	We proved that, under **conditions (i) and (ii)** (below Eq. (R6.4)), the *ab initio* probability of a given PWA is factorable into the product of an overall factor and contributions from local PWAs.	Section R6, **Eqs. (** **R6.7**,**R6.8** **)**, (see also Eqs. (R6.2,R6.3,R6.4))
Perturbation expansion (*ab initio* MSA probability)	The “perturbation expansion” of the *ab initio* probability of a given MSA, under a given model setting including a given phylogenetic tree.	Section R7, Eqs. (R7.2,R7.3,R7.4)
**Factorability (** ***ab initio*** **MSA probability)**	We proved that, under **conditions (i), (ii)** (below Eq. (R6.4) and **(iii)** (**Eq. (** **R7.8** **)**), the *ab initio* probability of a given MSA is factorable into the product of an overall factor and contributions from local MSAs.	Section R7, **Eq. (** **R7.9** **)**
**Totally space-homogeneous model**	Such a model gives factorable PWA probabilities, because the exit rate is an affine function of the sequence length (regardless of whether indel rates are time-dependent or not). The indel model of Dawg [26] and the “long indel” model [21] belong to this class.	Subsection R8-1, Eqs. (R8-1.1,R8-1.2), **Eqs. (** **R8-1.3**,**R8-1.4** **)**
**Equivalence (with caveat) of the “chop-zone” method and our** ***ab initio*** **method**	We showed that the “chop-zone” method in [21], adapted to calculate the probability of a given LHS equivalence class, is equivalent to our *ab initio* method, at least if the indel model is spatiotemporally homogeneous.	Subsection R8-1, **Supplementary appendix SA-3**
Model with simple insertion rate variation	If the deletion rates are space-homogeneous and the insertion rates depend only on the insertions’ flanking sites, the PWA probabilities are still factorable.	Subsection R8-1, Eq. (R8-1.5)
**Space-homogenous model flanked by essential sites**	This kind of model is a simplest example of the indel model whose *ab initio* PWA probabilities are **non-factorable**.	Subsection R8-2, **Eqs. (** **R8-2.1**,**R8-2.3** **)**
Degree of non-factorability	The “difference of exit-rate differences” (Eq. (R8-2.4)) could measure the “degree of non-factorability.”	Subsection R8-2, Eq. (R8-2.4)
Space-heterogeneous model with factorable PWA probability	We found that a class of indel models with rate-heterogeneity across regions (Eqs. (R8-3.1,R8-3.2)) have partially factorable PWA probabilities.	Subsection R8-3, **Eqs. (** **R8-3.1** **,** **R8-3.2** **)**, Eqs. (R8-3.3,R8-3.4,R8-3.5), Figure S3

NOTE: Especially important things are in boldface

We end this section with two notes. First, in this paper, the term “an evolutionary (or indel) process” means a series of successive mutation (or indel) events with both the order and the specific timing specified, and the term “an evolutionary (or indel) history” means a series of successive events with only the order specified. This usage should conform to the common practice in this field. Second, we will describe the results in the bra-ket notation, similar to that in quantum mechanics [29, 33]. However, those who are unfamiliar with the notation need not worry about it. Our formulation via the bra-ket notation can be proven to be equivalent to the standard formulation of the continuous-time Markov model via the vector-matrix notation. (We refer the interested readers to Supplementary appendix SA-1 in Additional file 2.) Therefore, if desired, the symbols of a bra (〈*x*|), a ket (|*y*〉), and an operator (*Ô*) could be regarded simply as convenient reminders of a row vector, a column vector, and a matrix, respectively.^4^

## Results and discussion

The key concepts and results proposed/obtained in this paper are summarized in Table 1. Readers can use the table to quickly grasp an overview of this paper, as well as to easily locate what they look for. Also, most mathematical symbols are briefly explained in Table S1 in Additional file 1.

### R1. Brief review of general SID model

**Fig. 2:**
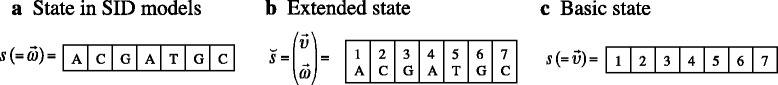
Sequence states. **a** A sequence state in the SID models [21]. Each site (cell) is assigned a residue state (A, T, G or C). **b** The corresponding extended sequence state in our evolutionary model. Each site is assigned an ancestry index (number) in addition to a residue state. **c** The corresponding basic sequence state. Each site is assigned an ancestry index alone. The $\overrightarrow{\omega}$ represents the set of residue states assigned to all sites. The $\overrightarrow{\upsilon}$ represents the set of ancestry indices assigned to all sites. (Note that the identical symbols (*s*’s) used in panels **a** and **c** represent different types of states (of the same sequence))

Miklós et al. [21] proposed a class of evolutionary models, which they called the “substitution/insertion/deletion (SID) models”. They are continuous-time Markov models defined on the space of strings (*i.e*., sequences) of any lengths, each of which consists of letters (*i.e*., residues, such as bases or amino acids) from a given alphabet (denoted as *Ω* here). Following [21], their state space will be denoted as: Ω*≡ ∪_*L* = 0 _^∞^*Ω*^*L*^, whose component, *Ω*^*L*^, is the space of all sequences of length *L*. If desired, a sequence state, *s* ∈ *Ω*^*L*^, could be represented as: *s* = [*ω*_1_, *ω*_2_, …, *ω*_*L*_] (with *ω*_*x*_ ∈ *Ω* for *x* = 1, 2, …, *L*) (see Fig. 2a). In this model, mutations are defined as transitions from a sequence state to another, and their instantaneous rates can be given via the following “rate grammar” they proposed:

- Substitution: $$ s={s}_L\omega {s}_R\overset{\rho_S\left({s}_L,\omega, {\omega}^{\prime },{s}_R\right)}{\to }{s}^{\prime }={s}_L{\omega}^{\prime }{s}_R; $$ (R1.1)
- Insertion: $$ s={s}_L{s}_R\overset{\rho_I\left({s}_L,{s}_I,{s}_R\right)}{\to }{s}^{\prime }={s}_L{s}_I{s}_R; $$ (R1.2)
- Deletion: $$ s={s}_L{s}_D{s}_R\overset{\rho_D\left({s}_L,{s}_D,{s}_R\right)}{\to }{s}^{\prime }={s}_L{s}_R. $$ (R1.3)

Here, *ρ*_*m*_(…) with *m* = *S*, *I and D* denote the rates of the substitution, the insertion, and the deletion, respectively, possibly depending on the arguments in the parentheses. In each of the above rules, *s* and *s*′, respectively, denote the sequence states before and after the mutation. The symbols *s*_*L*_ and *s*_*R*_ denote the subsequences flanking the mutated portion from the left and from the right, respectively.^5^ These SID models equipped with this “rate grammar” are genuine stochastic evolutionary models, and thus do not usually impose unnatural restrictions on the mutations (except possibly through restrictions on mutation rates). And the most general SID model can accommodate quite general mutation rates, including indel length distributions, by allowing their dependence on the sequence states before and after the mutation.^6^ As far as we know, however, this most general SID model was not theoretically examined further (at least thus far), maybe because adequate mathematical or conceptual tools were not devised and because of some other reasons (mentioned below). In the following sections, we will provide such tools, which in turn will help theoretically dissect an extended version of the most general SID model.

### R2. Ancestry indices and operator representation of mutations

First, we slightly extend the framework of the SID models, by assigning an ancestry to each site, which is a unit position in the sequence that accommodates a single residue. Hereafter, we will consider that it is the sites, instead of the residues, that are inserted/deleted. For example, the above example sequence state, *s* = [*ω*_1_, *ω*_2_, …, *ω*_*L*_](∈Ω^*L*^), can be extended as:$\breve {s}=\left[\left({\upsilon}_1,{\omega}_1\right),\left({\upsilon}_2,{\omega}_2\right),\dots, \left({\upsilon}_L,{\omega}_L\right)\right]\left(\in {\left(\varUpsilon \times \Omega \right)}^L\right)$ (Fig. 2b). Here, *υ*_*x*_(∈*ϒ*) is the ancestry index assigned to the *x*-th site of the sequence (with *x* = 1, 2, …, *L*). Alternatively, the extended sequence state could also be represented as: $\breve {s}=\left(\overrightarrow{\upsilon},\overrightarrow{\omega}\right),$^7^ where $\overrightarrow{\upsilon}=\left[{\upsilon}_1,{\upsilon}_2,\dots, {\upsilon}_L\right]\left(\in {\varUpsilon}^L\right)$ is an array of ancestry indices assigned to the sites, and $\overrightarrow{\omega}=\left[{\omega}_1,{\omega}_2,\dots, {\omega}_L\right]\left(\in {\Omega}^L\right)$ is an array of residue states that fill in the sites. (Note that $\overrightarrow{\omega}$ corresponds to the sequence state (*s*) in the SID models (in section R1)). The ancestry indices follow a number of rules: (i) different sites in the same sequence always have different ancestry indices; (ii) the ancestry index of a site remain unchanged as long as the site exists; and (iii) every time when an insertion takes place, new ancestry indices are assigned to the newly inserted sites. Other than these rules, the assignment of the indices is arbitrary. Especially, their values themselves are not so important. The most essential thing is whether two sites of different sequences share the same ancestry index or not; if so, the sites are mutually homologous (actually orthologous unless duplications are considered). Another important thing is the spatial relationship of the site having each ancestry with other sites, especially preserved ancestral sites (PASs, explained shortly). For the space of ancestry indices, *ϒ*, we will *tentatively* use the set of all positive integers (*Ν*_1_≡{1, 2, 3, …}), although there should be a more appropriate mathematical entity.^8^ Because of the rules imposed above, the space of the extended sequence states (denoted as ${\breve {s}}^{II}$ in [31]) is included in but never equal to {*ϒ* × Ω}^*^ = ∪_*L* = 0_^∞^{*ϒ* × Ω}^*L*^.

We devised the ancestry indices to facilitate the description of indel histories by keeping track of the evolutionary course of each site. For example, consider that a sequence whose initial state had the ancestry indices ${\overrightarrow{\upsilon}}_I=\left[1,2,3,4,5,6,7\right]$ evolved into the final state with ${\overrightarrow{\upsilon}}_F=\left[1,5,6,8,9,A,7\right]$. (Here the “A” is an abbreviation of 10.) Then, we can immediately infer what happened during its evolution by aligning the two sequences (represented by the arrays of ancestry indices):

R2.1 $$ \begin{array}{c}\hfill {\overrightarrow{\upsilon}}_I\kern1em 1\;2\;3\;4\;5\;6---7\hfill \\ {}\hfill {\overrightarrow{\upsilon}}_F\kern1em 1---5\;6\;8\;9\;A\;7\hfill \end{array}. $$

This alignment tells that the sites with ancestries 2, 3 and 4 were deleted and that the sites with ancestries 8, 9 and A were inserted. We can also see that the sites with 1, 5, 6 and 7 were preserved during the evolution. We will henceforth refer to such sites as “preserved ancestral sites (PASs)”. The PASs indicate that no indels occurred at or through the sites during the evolution under consideration. Thus, they can be used to narrow down the possible indel histories that might have resulted in the pairwise alignment (PWA) (as argued, *e.g*., in [21]). A fringe benefit of the ancestry indices is that they enable the mutation rates to vary beyond the dependence on the residue states (section R3). Hereafter, we refer to an array of ancestry indices (like $\overrightarrow{\upsilon}$) as a “basic sequence state” (abbreviated as a “sequence state” or a “basic state”), which is a backbone to be fleshed out by the residue states (like $\overrightarrow{\omega}$) to give the extended sequence state (like $\breve {s}$). Hereafter, the basic sequence state will often be denoted, *e.g*., as *s* (Fig. 2c). (Henceforth, symbols like *s* will never denote a residue state (like $\overrightarrow{\omega}$), which was a sequence state in section R1). And *S*^*II*^(⊂*ϒ* * = ∪_*L* = 0_^∞^*ϒ* ^*L*^) denotes the space of the basic states.

**Fig. 3:**
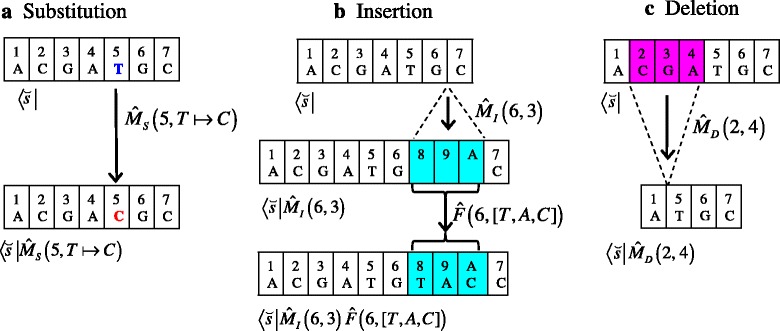
Operator representation of mutations. **a** A substitution operator, ${\widehat{M}}_S\left(5,\kern0.5em T\mapsto C\right)$. The residues before and after the substitution are in boldface in blue and red, respectively. **b** An insertion operator, ${\widehat{M}}_I\left(6,\kern0.5em 3\right)$, and a fill-in operator, $\widehat{F}\left(6,\kern0.5em \left[T,A,C\right]\right)$. The inserted sites are shaded in cyan. (Note that “A” at the top of the rightmost inserted column means the ancestry index of 10, not the residue state of A). **c** A deletion operator, ${\widehat{M}}_D\left(2,\kern0.5em 4\right)$. The sites to be deleted are shaded in magenta. In this figure, the extended sequence states were used for illustration. The bra-vector below each array denotes the state. The extended state, $\breve {s}$, is identical to that in Fig. 2b. Each vertical arrow indicates the action of the mutation operator beside it. Note that the first arguments of all operators and the second argument of the deletion operator specify positions along the sequence, and not ancestries (specified at the top of the sites)

**Fig. 4:**
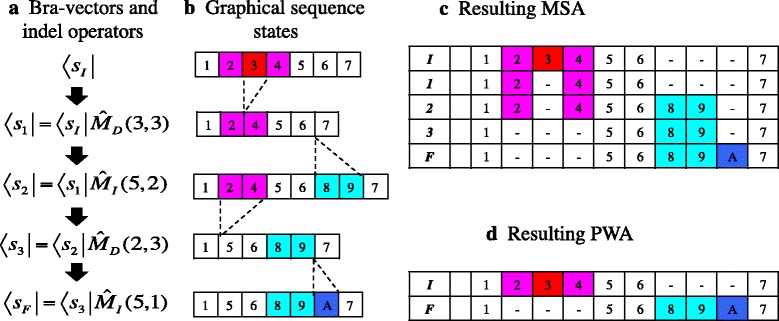
Example indel history and resulting alignments. **a** An example indel history in terms of the bra-vectors of sequence states and indel operators. **b** The graphical illustration of the history using basic sequence states. Each sequence state in panel **a** is horizontally aligned with its graphical representation in panel **b**. **c** The resulting MSA among the sequence states that the indel history went through. **d** The resulting PWA between the initial and final sequences. In both **c** and **d**, the bold italicized characters in the leftmost column are the suffixes indicating the sequence states in panel **a**. In panels **b**, **c**, and **d**, the number in each site (cell) represents its ancestry, but not necessarily its position along the sequence. The ‘A’ in the final sequence abbreviates 10. The same shading scheme as in Fig. 1 is used. The figure was adapted from Fig. 1 of [32]

Another, probably more important, tool we introduce here is the operator representation of mutations. When considering the evolutionary processes (or histories), we symbolically represent each (extended) sequence state as a bra-vector, like $\left\langle {\breve {s}}_I\right|$, which could be regarded as an abstract extension of a row vector in the normal representation of the continuous-time Markov model. Then, each mutation of a sequence can be represented as a linear operator (regarded as an abstract extension of a matrix) acting on a bra-vector (Fig. 3). Operator ${\widehat{M}}_S\left(x,\omega \mapsto {\omega}^{\prime}\right)$ denotes the substitution of the residue to *ω*^′^(≠*ω*) if it was *ω* at the *x* th site (or the null action otherwise) (Fig. 3a). Operator ${\widehat{M}}_I\left(x,l\right)$ denotes the insertion of *l* sites between the *x*-th and (*x* + 1)-th sites (Fig. 3b). The insertion operator *alone* does not determine the residue states of the inserted sites. It is the job of the “fill-in” operator, $\widehat{F}\left(x,\delta {\overrightarrow{\omega}}^{\prime}\left[l\right]\right)$, which fills in the *l* inserted sites with a new array of residues, $\delta {\overrightarrow{\omega}}^{\prime}\left[l\right]\left(\in {\Omega}^l\right)$. (This “division of labor” facilitates the decoupling of the substitution component and the indel component of an alignment probability. See Appendix A1 of [31] for more details). Finally, operator ${\widehat{M}}_D\left({x}_B,{x}_E\right)$ denotes the deletion of the subsequence between (and including) the *x*_*B*_-th and *x*_*E*_-th sites in the sequence immediately before the event (Fig. 3c). The action of multiple successive mutation events can be expressed as a product of mutation operators on an initial (extended) sequence state. For example, the indel history illustrated in panels a and b of Fig. 4 can be represented as a series of indel events, $\left[{\widehat{M}}_D\left(3,3\right),{\widehat{M}}_I\left(5,2\right),{\widehat{M}}_D\left(2,3\right),{\widehat{M}}_I\left(5,1\right)\right]$, on the initial basic state *s*_*I*_ (given above). Then, the final result of this indel history is expressed as: $\left\langle {s}_I\right|{\widehat{M}}_D\left(3,3\right){\widehat{M}}_I\left(5,2\right){\widehat{M}}_D\left(2,3\right){\widehat{M}}_I\left(5,1\right)$. Figure 4c shows the MSA among the initial, intermediate and final sequence states. Figure 4d shows the resulting PWA between the initial and final sequence states.

These new tools, the ancestry indices and the operator representation of mutations, will play essential roles in our theoretical development described below. In the SID models [21], each evolutionary process was expressed as a (time-recorded) trajectory of sequence states, each of which was represented by an array of residues (without ancestry assignments). In consequence, an instantaneous transition from a state to the next state was often expressed as a summation of multiple possible mutations. (For example, the transition from $\overrightarrow{\omega}=\left[A,A\right]$ to ${\overrightarrow{\omega}}^{\prime }=\left[A\right]$ could result from either ${\widehat{M}}_D\left(1,1\right)$ or ${\widehat{M}}_D\left(2,2\right)$). Ancestry indices help avoid such ambiguous channels by uniquely defining each instantaneous state-to-state transition as an action of a single mutation. (In the above example, the former causes the transition from $\overrightarrow{\upsilon}=\left[1,2\right]$ to ${\overrightarrow{\upsilon}}_{(1)}^{\prime }=\left[2\right]$, and the latter yields the transition from $\overrightarrow{\upsilon}$ to ${\overrightarrow{\upsilon}}_{(2)}^{\prime }=\left[1\right]$). This, in conjunction with the operator representation of mutations, enables us to shift the focus from the trajectory of sequence states to the history of mutations, especially indels. This shift of focus, as well as the “equivalence relations” between the products of operators (section R5), facilitates the examination of the factorability of alignment probabilities, as we will see in sections R4-R6.

### R3. Instantaneous transition (or rate) operator and finite-time transition operator

Now we are ready to define our evolutionary model, *i.e*., the general continuous-time Markov model to describe the evolution of an entire sequence along a time axis. If desired, this could be done by extending the rate grammar, Eqs. (R1.1,R1.2,R1.3), so that each mutation rate depend on the entire extended sequence states (*i.e*., not only on the residue states) immediately before and after the mutation. (We will also introduce the explicit time dependence. This may allow us to incorporate the effects of changes in the physiology, genomic contexts, external environments, etc. (See also section 2.4 of [32].)) However, to define the model more neatly, we will parametrize the mutation rates in coordination with the mutation operators defined in section R2. Although the parametrization is different from (the extended version of) Eqs. (R1.1,R1.2,R1.3), the two sets of mutation rates are equivalent. (To remember these differences in the parametrization, we will use the symbol *r*_*m*_(…) (*m* = *S*, *I and D*) instead of *ρ*_*m*_(…).) Let $s$ be the extended sequence state immediately before the mutation. And let *t* be the time at which the mutation occurred. Then, ${r}_S\left(x,\omega \mapsto {\omega}^{\hbox{'}};\breve {s},t\right)$ is the rate of the substitution, ${\widehat{M}}_S\left(x,\omega \mapsto {\omega}^{\prime}\right)$. It must be zero unless *ω* is at the *x*-th site of $\breve {s}$. ${r}_I\left(x,l,\delta {\overrightarrow{\omega}}^{\prime}\left[l\right];\breve {s},t\right)$ is the rate of the insertion, ${\widehat{M}}_I\left(x,l\right)$ (accompanied by $\widehat{F}\left(x,\delta {\overrightarrow{\omega}}^{\prime}\left[l\right]\right)$). And ${r}_D\left({x}_B,{x}_E;\breve {s},t\right)$ is the rate of the deletion, ${\widehat{M}}_D\left({x}_B,{x}_E\right)$. Using these mutation rates that accompany the mutation operators, we can define our evolutionary model in a manner closer to the standard, by defining the instantaneous transition rate operator (or the “rate operator” for short), which is an analog of the instantaneous transition rate matrix in a continuous-time Markov model. Because the state space we are working in, ${\breve {s}}^{II}$, is essentially infinite, we cannot give the explicit matrix expression of the rate operator on the entire state space. Nevertheless, the rate operator can be defined if we give its action on every state in ${\breve {s}}^{II}$. Let ${\widehat{Q}}^{SID}(t)$ denote our rate operator (at time *t*). It is convenient to decompose it as follows:

R3.1 $$ {\widehat{Q}}^{SID}(t)={\widehat{Q}}^S(t)+{\widehat{Q}}^I(t)+{\widehat{Q}}^D(t), $$

where ${\widehat{Q}}^m(t)$ with *m* = *S*, *I and D*, respectively, are the substitution, insertion, and deletion components of the rate operator. Each of the three components can be further decomposed as:

R3.2 $$ {\widehat{Q}}^m(t)={\widehat{Q}}_M^m(t)+{\widehat{Q}}_X^m(t)\kern1em \left(m=S,I\kern0.24em  and\kern0.24em D\right). $$

Here, the “mutation part”, ${\widehat{Q}}_M^m(t)$, describes the transitions to different states via mutations of type *m* (=*S*, *I or D*). And the “exit rate part”, ${\widehat{Q}}_X^m(t)$, attenuates the state retention probability at the exit rate, ${R}_X^m\left(\breve {s},t\right)$, which is determined by the type *m* mutations. It guarantees that the state probabilities sum up to unity at any time. Specifically, the mutation parts are defined by the following actions on every state:

R3.3 $$ \left\langle \overset{\smile }{s}\right|{\widehat{Q}}_M^S(t)\equiv {\displaystyle {\sum}_{x=1}^{L\left(\breve {s}\right)}{\displaystyle {\sum}_{\begin{array}{l}{\omega}^{\prime}\in \Omega, \\ {}{\omega}^{\hbox{'}}\ne {\omega}_x\left(\overset{\smile }{s}\right)\end{array}}\left[{r}_S\left(x,{\omega}_x\left(\overset{\smile }{s}\right)\mapsto {\omega}^{\hbox{'}};\breve {s},t\right)\times \left\langle \breve {s}\right|{\widehat{M}}_S\left(x,{\omega}_x\left(\breve {s}\right)\mapsto {\omega}^{\hbox{'}}\right)\right]}}, $$

R3.4 $$ \left\langle \breve {s}\right|{\widehat{Q}}_M^I(t)\equiv {\displaystyle {\sum}_{x=0}^{L\left(\breve {s}\right)}{\displaystyle {\sum}_{l=1}^{\infty }{\displaystyle {\sum}_{\delta {\overrightarrow{\omega}}^{\prime}\left[l\right]\in {\Omega}^l}\left[{r}_I\left(x,l,\delta {\overrightarrow{\omega}}^{\prime}\left[l\right];\breve {s},t\right)\times \left\langle \breve {s}\right|{\widehat{M}}_I\left(x,l\right)\widehat{F}\left(x,\delta {\overrightarrow{\omega}}^{\prime}\left[l\right]\right)\right]}}}, $$

R3.5 $$ \left\langle \breve {s}\right|{\widehat{Q}}_M^D(t)\equiv {\displaystyle {\sum}_{x_B=-\infty}^{L\left(\breve {s}\right)}{\displaystyle {\sum}_{x_E= max\left\{1,{x}_B\right\}}^{+\infty}\left[{r}_D\left({x}_B,{x}_E;\breve {s},t\right)\times \left\langle \breve {s}\right|{\widehat{M}}_D\left({x}_B,{x}_E\right)\right]}}. $$

Here, $L\left(\breve {s}\right)$ denotes the length (*i.e*., the number of sites) of $\breve {s}$, and ${\omega}_x\left(\breve {s}\right)$ denotes the residue at the *x*-th site of $\breve {s}$. Figure 3 exemplifies the states on the right hand sides of Eqs. (R3.3-R3.5). The terms with *x* = 0 and with $x=L\left(\breve {s}\right)$ in Eq. (R3.4) represent insertions at the left and right ends, respectively, of the sequence. How to deal with such insertions varies depending on various factors including the model setting, and can be implemented by adjusting the insertion rates accordingly (see, *e.g*., [21, 26]). The terms with *x*_*B*_ < 1 or ${x}_E>L\left(\breve {s}\right)$ in Eq. (R3.5) represent the deletions of the subsequences sticking out of the subject sequence. These terms were included because the subject sequence is regarded as embedded in a “chromosome” with a virtually infinite length (*e.g*., [21, 26]).^9^ The exit rate parts are defined in nearly the same form:

R3.6 $$ \left\langle \breve {s}\right|{\widehat{Q}}_X^m(t)\equiv -{R}_X^m\left(\breve {s},t\right)\left\langle \breve {s}\right|\kern1em \left(m=S,I\kern0.24em  and\kern0.24em D\right). $$

They differ only in the exit rates:

R3.7 $$ {R}_X^S\left(\breve {s},t\right)={\displaystyle {\sum}_{x=1}^{L\left(\breve {s}\right)}{\displaystyle {\sum}_{\begin{array}{l}{\omega}^{\prime}\in \Omega, \\ {}{\omega}^{\prime}\ne {\omega}_x\left(\breve {s}\right)\end{array}}{r}_S\left(x,{\omega}_x\left(\breve {s}\right)\mapsto {\omega}^{\hbox{'}};\breve {s},t\right)}}, $$

R3.8 $$ {R}_X^I\left(\breve {s},t\right)={\displaystyle {\sum}_{x=0}^{L\left(\breve {s}\right)}{\displaystyle {\sum}_{l=1}^{\infty }{\displaystyle {\sum}_{\delta {\overrightarrow{\omega}}^{\prime}\left[l\right]\in {\Omega}^l}{r}_I\left(x,l,\delta {\overrightarrow{\omega}}^{\prime}\left[l\right];\breve {s},t\right)}}}, $$

R3.9 $$ {R}_X^D\left(\breve {s},t\right)={\displaystyle {\sum}_{x_B=-\infty}^{L\left(\breve {s}\right)}{\displaystyle {\sum}_{x_E= max\left\{1,{x}_B\right\}}^{+\infty }{r}_D\left({x}_B,{x}_E;\breve {s},t\right)}}. $$

These equations, Eqs. (R3.1-R3.9), cooperatively define the instantaneous transition rate operator, ${\widehat{Q}}^{SID}(t)$, and thus define our evolutionary model. If desired, ${\widehat{Q}}^{SID}(t)$ could be decomposed as:

R3.10 $$ {\widehat{Q}}^{SID}(t)={\widehat{Q}}_M^{SID}(t)+{\widehat{Q}}_X^{SID}(t). $$

Here ${\widehat{Q}}_M^{SID}(t)\equiv {\widehat{Q}}_M^S(t)+{\widehat{Q}}_M^I(t)+{\widehat{Q}}_M^D(t)$ is the collection of all mutational transition terms, and ${\widehat{Q}}_X^{SID}(t)\equiv {\widehat{Q}}_X^S(t)+{\widehat{Q}}_X^I(t)+{\widehat{Q}}_X^D(t)$ is the entire exit rate part, which attenuates the state retention probability at the total exit rate, ${R}_X^{SID}\left(\breve {s},t\right)\equiv {R}_X^S\left(\breve {s},t\right)+{R}_X^I\left(\breve {s},t\right)+{R}_X^D\left(\breve {s},t\right).$ Actually, the total mutational part, ${\widehat{Q}}_M^{SID}(t)$, is equivalent to (the extended version of) the “instantaneous rate matrix” of the general SID model (defined by Eq. (1) in [21]), although the two expressions appear quite different from each other. The difference mainly stems from the parametrization and the state representation. We use our parametrization because we believe it to clarify the meaning of each term in the rate operator. And, in this section, the sequence state after the mutation (say, $\left\langle {\breve {s}}^{\prime}\right|$) was represented as the result of the mutation operator (say, ${\widehat{M}}_m\left(\dots \right)$) acting on the state before the mutation (say, $\left\langle \breve {s}\right|$), like $\left\langle {\breve {s}}^{\prime}\right|=\left\langle \breve {s}\right|{\widehat{M}}_m\left(\dots \right)$. This is legitimate because a mutation transfers a subject state uniquely to another state. This state representation will facilitate the unfolding of our theory below.

Thus far, we included substitutions (and residue states) mainly in order to discuss the relationship between our evolutionary model and the general SID model. However, our main interest here is in calculating the probability of the *skeleton* of a sequence alignment, composed only of the sites and gaps, which will then be filled in with residues to form a full alignment. Because such a skeleton can be created only through an evolutionary process of indels, we will hereafter omit the description of substitutions and the resulting changes in the residue state ($\overrightarrow{\omega}$). (This includes omitting the “fill-in” operators, $\widehat{F}\left(x,\delta {\overrightarrow{\omega}}^{\prime}\left[l\right]\right)$’s.) (Henceforth, the alignment skeleton will be called the “alignment” for short.) But we will retain the basic sequence state consisting of ancestry indices ($s=\overrightarrow{\upsilon}$). The rate heterogeneity will be realized only through the ancestry dependence. This would be almost sufficient for representing the dependence of the rates on, *e.g*., the epigenetic or 3D structural context (*e.g*., [25]). And this may also be able to approximate the dependence on some residue state contexts, especially in highly conserved regions. Now, the rate operator defined by Eqs. (R3.1-R3.9) is reduced as follows. (The reduced total rate operator will be denoted as ${\widehat{Q}}^{ID}(t)$).

R3.11 $$ {\widehat{Q}}^{ID}(t)={\widehat{Q}}^I(t)+{\widehat{Q}}^D(t), $$

R3.12 $$ \left\langle s\right|{\widehat{Q}}_M^I(t)\equiv {\displaystyle {\sum}_{x=0}^{L(s)}{\displaystyle {\sum}_{l=1}^{\infty }{r}_I\left(x,l;s,t\right)\left\langle s\right|{\widehat{M}}_I\left(x,l\right)}}, $$

R3.13 $$ \left\langle s\right|{\widehat{Q}}_M^D(t)\equiv {\displaystyle {\sum}_{x_B=-\infty}^{L(s)}{\displaystyle {\sum}_{x_E= max\left\{1,{x}_B\right\}}^{+\infty }{r}_D\left({x}_B,{x}_E,s,t\right)\left\langle s\right|{\widehat{M}}_D\left({x}_B,{x}_E\right)}}, $$

R3.14 $$ {R}_X^I\left(s,t\right)={\displaystyle {\sum}_{x=0}^{L(s)}{\displaystyle {\sum}_{l=1}^{\infty }{r}_I\left(x,l;s,t\right)}}, $$

R3.15 $$ {R}_X^D\left(s,t\right)={\displaystyle {\sum}_{x_B=-\infty}^{L(s)}{\displaystyle {\sum}_{x_E= max\left\{1,{x}_B\right\}}^{+\infty }{r}_D\left({x}_B,{x}_E;s,t\right)}}. $$

Equations (R3.2,R3.6) remain unchanged except the exclusion of *m* = *S* and the replacement of $\breve {s}$ by *s*. In Eqs. (R3.12,R3.14), the insertion rates could be related to those in Eqs. (R3.4,R3.8) by the equation:

R3.16 $$ {r}_I\left(x,l;s,t\right)\equiv {\displaystyle {\sum}_{\delta {\overrightarrow{\omega}}^{\prime}\left[l\right]\in {\Omega}^l}{r}_I\left(x,l,\delta {\overrightarrow{\omega}}^{\prime}\left[l\right];s,t\right)}, $$

where the $\overrightarrow{\omega}$-dependence of the right hand side was omitted.

Now we can calculate the operator that gives the finite-time transition probabilities between states. We will call it the “finite-time transition operator”. Let ${\widehat{P}}^{ID}\left(t,{t}^{\prime}\right)$ be such an operator describing the state transition via indels alone from an initial time, *t*, to a final time, *t*^′^ (> *t*). Operator ${\widehat{P}}^{ID}\left(t,{t}^{\prime}\right)$ is defined to give the following equations:

R3.17 $$ \left\langle s\right|{\widehat{P}}^{ID}\left(t,{t}^{\prime}\right)\left|{s}^{\prime}\right\rangle =P\left[\left({s}^{\prime },t\hbox{'}\right)\left|\left(s,t\right)\right.\right]\kern0.36em for\kern1em {}^{\forall}\left(s,{s}^{\prime}\right)\in {\left({S}^{II}\right)}^2. $$

On the right hand side, *P*[(*s*^′^, *t* ^′^)|(*s*, *t*)] is the probability that the sequence is in state *s*^′^ at time *t*^′^ conditioned on that it was in state *s* at time *t*. On the left hand side, |*s*^′^〉 denotes a ket-vector (an abstract extension of a column vector), whose exclusive role here is to give “inner-products” with bra-vectors: 〈*s*| *s*^′^〉 = 1 (if *s* = *s*^′^), = 0 (otherwise). From the evolutionary principle that our model must satisfy, or equivalently, from the fundamental properties (such as the Chapman-Kolmogorov equation) of the continuous-time Markov model, the finite-time transition operator must be given by the multiplicative accumulation of the effects of the rate operators along the time axis. Thus, ${\widehat{P}}^{ID}\left(t,{t}^{\prime}\right)$ is formally calculated as:

R3.18 $$ \begin{array}{c}{\widehat{P}}^{ID}\left(t,{t}^{\prime}\right)=\underset{N_P\to \infty }{lim}\left(\widehat{I}+{\scriptscriptstyle \frac{t^{\prime }-t}{N_P}}{\widehat{Q}}^{ID}\left({t}_1^{\left({N}_P\right)}\right)\right)\left(\widehat{I}+{\scriptscriptstyle \frac{t^{\prime }-t}{N_P}}{\widehat{Q}}^{ID}\left({t}_2^{\left({N}_P\right)}\right)\right)\cdots \left(\widehat{I}+{\scriptscriptstyle \frac{t^{\prime }-t}{N_P}}{\widehat{Q}}^{ID}\left({t}_{N_P}^{\left({N}_P\right)}\right)\right)\\ {}\equiv T\left\{ exp\left({\displaystyle {\int}_t^{t^{\prime }}d\tau {\widehat{Q}}^{ID}\left(\tau \right)}\right)\right\}.\end{array} $$

In the middle of the equation, *Î* is the identity operator (i.e., 〈*s*|*Î* = 〈*s*| for every *s* ∈ *S*^*II*^), and ${t}_k^{\left({N}_P\right)}\equiv t+\left(k-{\scriptscriptstyle \frac{1}{2}}\right){\scriptscriptstyle \frac{t^{\prime }-t}{N_P}}$. On the rightmost side, *T*{…} denotes the meta-operator that rearranges the operators in each operator product term in the temporal order so that the earliest operator comes leftmost. Another way to give ${\widehat{P}}^{ID}\left(t,{t}^{\prime}\right)$ is through the first-order time-differential equation. Again, from the fundamental properties of the continuous-time Markov model, or equivalently, from Eq. (R3.18), we can show that the operator satisfies the following differential equations:

R3.19 $$ \frac{\partial }{\partial {t}^{\prime }}{\widehat{P}}^{ID}\left(t,{t}^{\prime}\right)={\widehat{P}}^{ID}\left(t,{t}^{\prime}\right){\widehat{Q}}^{ID}\left({t}^{\prime}\right), $$

R3.20 $$ \frac{\partial }{\partial t}{\widehat{P}}^{ID}\left(t,{t}^{\prime}\right)=-{\widehat{Q}}^{ID}(t){\widehat{P}}^{ID}\left(t,{t}^{\prime}\right). $$

Equation (R3.19) is the “forward equation”, and Eq. (R3.20) is the “backward equation”. And the evolutionary principle naturally includes the following equation:

R3.21 $$ {\widehat{P}}^{ID}\left(t,t\right)=\widehat{I}\;for\;{}^{\forall }t\in \left[{t}_I,{t}_F\right], $$

where [*t*_*I*_, *t*_*F*_] is the time interval in which the model is defined. This equation could be used as the initial condition for each of Eqs. (R3.19,R3.20). In the next section, we will obtain the solutions for Eqs. (R3.19, R3.20, R3.21) in a more tractable form than the defining solution, Eq. (R3.18).

### R4. Perturbation expansion of finite-time transition operator and pairwise alignment probability: brief description

In time-dependent perturbation theory of quantum mechanics (*e.g*., [29, 30]), the instantaneous time evolution operator (Hamiltonian *Ĥ*(*t*)) is considered as a sum of two operators, $\widehat{H}(t)={\widehat{H}}_0(t)+\widehat{V}(t)$, and the time evolution of the system is described as if the system mostly evolves according to the well-solvable instantaneous time-evolution operator (*Ĥ*_0_(*t*)) and is occasionally perturbed by the “interaction” operator ($\widehat{V}(t)$). We adapt such a technique of time-dependent perturbation expansion to our evolutionary model. Here, we briefly describe the results. For their detailed derivations, see Supplementary methods SM-1 in Additional file 1. We first re-express our rate operator as:

R4.1 $$ {\widehat{Q}}^{ID}(t)={\widehat{Q}}_0^{ID}(t)+{\widehat{Q}}_M^{ID}(t). $$

Here ${\widehat{Q}}_0^{ID}(t)\equiv {\widehat{Q}}_X^I(t)+{\widehat{Q}}_X^D(t)$ describes the mutation-free evolution, and ${\widehat{Q}}_M^{ID}(t)\equiv {\widehat{Q}}_M^I(t)+{\widehat{Q}}_M^D(t)$ describes the single-mutation transition between states. From the reduced form of Eq. (R3.6), we get:

R4.2 $$ \left\langle s\right|{\widehat{Q}}_0^{ID}(t)=-{R}_X^{ID}\left(s,t\right)\left\langle s\right|, $$

R4.3 $$ \mathrm{with}\kern1em {R}_X^{ID}\left(s,t\right)\equiv {R}_X^I\left(s,t\right)+{R}_X^D\left(s,t\right). $$

Using Eq. (R4.1), the forward equation (Eq. (R3.19)) accompanied by the initial condition (Eq. (R3.21)) can be shown to be equivalent to a crucial integral equation:

R4.4 $$ {\widehat{P}}^{ID}\left(t,{t}^{\prime}\right)={\widehat{P}}_0^{ID}\left(t,{t}^{\prime}\right)+{\displaystyle {\int}_t^{t^{\prime }}d\tau {\widehat{P}}^{ID}\left(t,\tau \right){\widehat{Q}}_M^{ID}\left(\tau \right){\widehat{P}}_0^{ID}\left(\tau, {t}^{\prime}\right)}. $$

Here, ${\widehat{P}}_0^{ID}\left({t}^{\prime },{t}^{\prime \prime}\right)\equiv T\left\{ exp\left({\displaystyle {\int}_{t^{\prime}}^{t^{\prime \prime }}d\tau {\widehat{Q}}_0^{ID}\left(\tau \right)}\right)\right\}$. Similarly, the backward equation (Eq. (R3.20)) accompanied by Eq. (R3.21) is equivalent to another crucial integral equation:

R4.5 $$ {\widehat{P}}^{ID}\left(t,{t}^{\prime}\right)={\widehat{P}}_0^{ID}\left(t,{t}^{\prime}\right)+{\displaystyle {\int}_t^{t^{\prime }}d\tau {\widehat{P}}_0^{ID}\left(t,\tau \right){\widehat{Q}}_M^{ID}\left(\tau \right){\widehat{P}}^{ID}\left(\tau, {t}^{\prime}\right)}. $$

Now, to formally solve Eq. (R4.4), we assume that the solution can be expanded as: ${\widehat{P}}^{ID}\left(t,{t}^{\prime}\right)={\displaystyle {\sum}_{N=0}^{\infty }{\widehat{P}}_{(N)}^{ID}\left(t,{t}^{\prime}\right)}$, where ${\widehat{P}}_{(N)}^{ID}\left(t,{t}^{\prime}\right)$ is the collection of terms containing *N* indel operators each. Substituting this expansion into Eq. (R4.4) and performing some formal calculations, we get the final form of the *ab initio* solution we desire:

R4.6 $$ \left\langle {s}_0\right|{\widehat{P}}^{ID}\left({t}_I,{t}_F\right)={\displaystyle \sum_{N=0}^{\infty }{\displaystyle \sum_{\left[{\widehat{M}}_1,{\widehat{M}}_2,,,\cdots, {\widehat{M}}_N\right]\in {H}^{ID}\left(N;{s}_0\right)}P\left[\left(\left[{\widehat{M}}_1,{\widehat{M}}_2,,,\dots, {\widehat{M}}_N\right],,,\left[{t}_I,{t}_F\right]\right)\left|\left({s}_0,{t}_I\right)\right.\right]}\left\langle {s}_0\right|{\widehat{M}}_1}{\widehat{M}}_2\cdots {\widehat{M}}_N. $$

Here, Η^*ID*^(*N*; *s*_0_) denotes the space of all possible histories of *N* indels each beginning with the sequence state, *s*_0_. And

R4.7 $$ \begin{array}{l}P\left[\left(\left[{\widehat{M}}_1,{\widehat{M}}_2,\cdots, {\widehat{M}}_N\right],\left[{t}_I,{t}_F\right]\right)\left|\left({s}_0,{t}_I\right)\right.\right]\\ {}={\displaystyle \underset{t_I={\tau}_0<{\tau}_1<\cdots <{\tau}_N<{\tau}_{N+1}={t}_F}{{\displaystyle \int \cdots {\displaystyle \int }}}}d{\tau}_1\cdots d{\tau}_N\left({\displaystyle {\prod}_{\nu =1}^Nr\left({\widehat{M}}_{\nu };{s}_{\nu -1},{\tau}_{\nu}\right)}\right) \exp \left\{-{\displaystyle \sum_{\nu =0}^N{\displaystyle {\int}_{\tau_{\nu}}^{\tau_{\nu +1}}d\tau {R}_X^{ID}\left({s}_{\nu },\tau \right)}}\right\}\left|{}_{\left\{\left\langle {s}_{\nu}\right|=\left\langle {s}_{\nu -1}\right|{\widehat{M}}_{\nu}\left|\nu =1,\dots, N\right.\right\}}\right.\end{array} $$

is the probability that an *N* -event indel history, $\left[{\widehat{M}}_1,{\widehat{M}}_2,\cdots, {\widehat{M}}_N\right]$ (with ${\widehat{M}}_{\nu }$ (*ν* = 1, 2, …, *N*) being the *ν*-th event), occurred during time interval [*t*_*I*_, *t*_*F*_], given an initial sequence state (*s*_0_) at time *t*_*I*_. The rate, $r\left({\widehat{M}}_{\nu };{s}_{\nu -1},{\tau}_{\nu}\right)$, is *r*_*I*_(*x*, *l*; *s*_*ν* − 1_, *τ*_*ν*_) if ${\widehat{M}}_{\nu }={\widehat{M}}_I\left(x,l\right)$, and it is *r*_*D*_(*x*_*B*_, *x*_*E*_; *s*_*ν* − 1_, *τ*_*ν*_) if ${\widehat{M}}_{\nu }={\widehat{M}}_D\left({x}_B,{x}_E\right)$. It should be noted that *Η*^*ID*^(*N* = 0; *s*_0_)≡{(*s*_0_, [])} consists only of the history with zero indel, [ ], whose conditional probability is: $P\left[\left(\left[\right],\left[{t}_I,{t}_F\right]\right)\left|\left({s}_0,{t}_I\right)\right.\right]= \exp \left\{-{\displaystyle {\int}_{t_I}^{t_F}d\tau {R}_X^{ID}\left({s}_0,\tau \right)}\right\}$. Eq. (R4.6) supplemented with Eq. (R4.7) is also the solution of Eq. (R4.5). (Mathematically, Eq. (R4.7) is a multiple-time integral over all possible timing, whose integrand is the probability density of an evolutionary process of *N* indels with particular timing, (*τ*_1_, *τ*_2_, …, *τ*_*N*_)).

Equation (R4.6) states that the finite-time transition operator (acting on 〈*s*_0_|) is the collection of the effects of all possible indel histories starting with *s*_0_, each weighted by its probability (Eq. (R4.7)). Thus, it mathematically underpins Gillespie’s [34] famous stochastic simulation algorithm, which provides the basis of genuine molecular evolution simulators (*e.g*., [26–28]). Our derivation of Eq. (R4.6) and Eq. (R4.7) through the integral equation (Eq. (R4.4) or Eq. (R4.5)) bridges Gillespie’s own intuitive reasoning and Feller’s [35] mathematically rigorous proof of the solution.

Now, substitute an “ancestral” sequence state, *s*^*A*^(∈*S*^*II*^), for *s*_0_ in Eq. (R4.6), and take the inner product between it and the ket-vector, |*s*^*D*^〉, of a “descendant” sequence state, *s*^*D*^(∈*S*^*II*^). Comparing the two sequence states in *S*^*II*^ naturally gives a PWA, *α*(*s*^*A*^, *s*^*D*^) (*e.g*., Eq. (R2.1)).^10^ Hence, the summation of $\left\langle {s}^A\right|{\widehat{P}}^{ID}\left({t}_I,{t}_F\right)\left|{s}^D\right\rangle$’s over all “equivalent” *s*^*D*^’s providing the same *α*(*s*^*A*^, *s*^*D*^) must be *P*[(*α*(*s*^*A*^, *s*^*D*^), [*t*_*I*_, *t*_*F*_])|(*s*^*A*^, *t*_*I*_)], which is the probability that *α*(*s*^*A*^, *s*^*D*^) results from sequence evolution during [*t*_*I*_, *t*_*F*_], given *s*^*A*^ at *t*_*I*_. Similarly to the derivation of Eq. (R4.6), we obtain its formal expression as:

R4.8 $$ P\left[\left(\alpha \left({s}^A,{s}^D\right),\left[{t}_I,{t}_F\right]\right)\left|\left({s}^A,{t}_I\right)\right.\right]={\displaystyle \sum_{\begin{array}{l}\kern2em N=\\ {}{N}_{min}\left[\alpha \left({s}^A,{s}^D\right)\right]\end{array}}^{\infty }{\displaystyle \sum_{\begin{array}{l}\left[{\widehat{M}}_1,{\widehat{M}}_2,\cdots, {\widehat{M}}_N\right]\\ {}\kern1em \in {\mathrm{H}}^{ID}\left[N;\alpha \left({s}^A,{s}^D\right)\right]\end{array}}P\left[\left(\left[{\widehat{M}}_1,{\widehat{M}}_2,\cdots, {\widehat{M}}_N\right],\left[{t}_I,{t}_F\right]\right)\left|\left({s}^A,{t}_I\right)\right.\right]}}. $$

Here, *Η*^*ID*^[*N*; *α*(*s*^*A*^, *s*^*D*^)] denotes the set of all indel histories with *N* indels each that can result in *α*(*s*^*A*^, *s*^*D*^), and *N*_*min*_[*α*(*s*^*A*^, *s*^*D*^)] is the minimum number of indels required for creating the PWA.

Using the set of all PWA-consistent histories, ${\tilde{H}}^{ID}\left[\alpha \left({s}^A,{s}^D\right)\right]\equiv {\displaystyle {\cup}_{N={N}_{min}\left[\alpha \left({s}^A,{s}^D\right)\right]}^{\infty }{H}^{ID}\left[N;\alpha \left({s}^A,{s}^D\right)\right]},$ Eq. (R4.8) can be further simplified as:

R4.9 $$ P\left[\left(\alpha \left({s}^A,{s}^D\right),\left[{t}_I,{t}_F\right]\right)\left|\left({s}^A,{t}_I\right)\right.\right]={\displaystyle \sum_{\begin{array}{l}\left[{\widehat{M}}_1,{\widehat{M}}_2,\cdots, {\widehat{M}}_N\right]\\ {}\kern1em \in {\tilde{H}}^{ID}\left[\alpha \left({s}^A,, {s}^D\right)\right]\end{array}}P\left[\left(\left[{\widehat{M}}_1,{\widehat{M}}_2,\cdots, {\widehat{M}}_N\right],\left[{t}_I,{t}_F\right]\right)\left|\left({s}^A,{t}_I\right)\right.\right]}. $$

Equation (R4.8) and Eq. (R4.9) are the formal expressions of the occurrence probability of *α*(*s*^*A*^, *s*^*D*^) derived in effect from the defining equations, Eqs. (R3.19, R3.20, R3.21), of our evolutionary model. Thus, they are the “*ab initio* probability” of the PWA. In the following, we will examine its factorability.

### R5. Local history-set equivalence class of indel histories

**Fig. 5:**
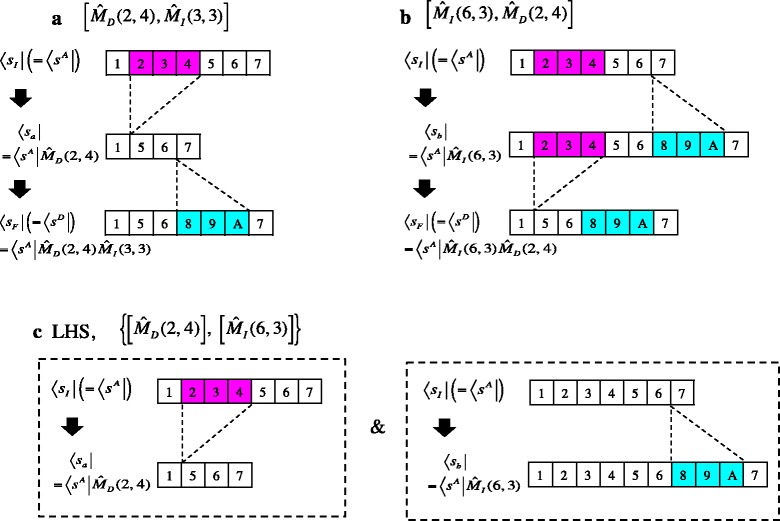
Binary equivalence relation and LHS equivalence class. **a** An indel history, $\left[{\widehat{M}}_D\left(2,\kern0.5em 4\right),\kern0.5em {\widehat{M}}_I\left(3,\kern0.5em 3\right)\right]$. **b** Another indel history, $\left[{\widehat{M}}_I\left(6,\kern0.0em 3\right),\kern0.5em {\widehat{M}}_D\left(2,\kern0.0em 4\right)\right]$. These histories result in the same final state (〈*s*_*F*_| (= 〈*s* ^*D*^|)). Thus, their total effects are equivalent. **c** Their equivalent local history set (LHS), $\left\{\left[{\widehat{M}}_D\left(2,\kern0.0em 4\right)\right],\kern1em \left[{\widehat{M}}_I\left(6,\kern0.0em 3\right)\right]\right\}$, is represented by the isolated actions of local indel histories on the initial state (each enclosed in a dashed box)

Before advancing to the factorability of general PWA probabilities, we will introduce an essential concept here. For this purpose, we first consider the very simple PWA, Eq. (R2.1), as an example. (Here we make the substitutions, ${s}^A={\overrightarrow{\upsilon}}_I$ and ${s}^D={\overrightarrow{\upsilon}}_F$). In this case, *N*_*min*_[*α*(*s*^*A*^, *s*^*D*^)] = 2, and there are two 2-indel histories that can yield this PWA: one is $\left[{\widehat{M}}_D\left(2,4\right),{\widehat{M}}_I\left(3,3\right)\right]$ (Fig. 5a) and the other is $\left[{\widehat{M}}_I\left(6,3\right),{\widehat{M}}_D\left(2,4\right)\right]$ (Fig. 5b). Thus, these two indel histories result in the same final state: $\left\langle {s}_F\right|=\left\langle {s}_I\right|{\widehat{M}}_D\left(2,4\right){\widehat{M}}_I\left(3,3\right)=\left\langle {s}_I\right|{\widehat{M}}_I\left(6,3\right){\widehat{M}}_D\left(2,4\right)$ (Fig. 5, panels a and b). In other words, the two different successive actions of two indel operators have the same effect on the sequence states (in space *S*^*II*^). This fact will be phrased as “the two products of the operators are *equivalent*”, and represented by the relationship:

R5.1 $$ {\widehat{M}}_I\left(6,3\right){\widehat{M}}_D\left(2,4\right)\sim {\widehat{M}}_D\left(2,4\right){\widehat{M}}_I\left(3,3\right). $$

This “binary equivalence” can be generalized to the following relationships between two indel events separated at least by a PAS:

R5.2a $$ {\widehat{M}}_I\left({x}_1,{l}_1\right){\widehat{M}}_I\left({x}_2,{l}_2\right)\sim {\widehat{M}}_I\left({x}_2,{l}_2\right){\widehat{M}}_I\left({x}_1+{l}_2,{l}_1\right)\;for\;{x}_1>{x}_2, $$

R5.2b $$ {\widehat{M}}_D\left({x}_B,{x}_E\right){\widehat{M}}_I\left(x,l\right)\sim {\widehat{M}}_I\left(x,l\right){\widehat{M}}_D\left({x}_B+l,{x}_E+l\right)\;for\;{x}_B>x+1, $$

R5.2c $$ {\widehat{M}}_I\left(x,l\right){\widehat{M}}_D\left({x}_B,{x}_E\right)\sim {\widehat{M}}_D\left({x}_B,{x}_E\right){\widehat{M}}_I\left(x-{l}^{\prime },l\right)\;for\;x>{x}_E, $$

R5.2d $$ {\widehat{M}}_D\left({x}_{B1},{x}_{E1}\right){\widehat{M}}_D\left({x}_{B2},{x}_{E2}\right)\sim {\widehat{M}}_D\left({x}_{B2},{x}_{E2}\right){\widehat{M}}_D\left({x}_{B1}-{l}_2^{\prime },{x}_{E1}-{l}_2^{\prime}\right)\;for\;{x}_{B1}>{x}_{E2}+1. $$

Here, *l*^′^≡*min*{*x*_*E*_ − *x*_*B*_ + 1, *x*_*E*_} in Eq. (R5.2c), and *l*_2_^′^≡*min*{*x*_*E*2_ − *x*_*B*2_ + 1, *x*_*E*2_} in Eq. (R5.2d). If you will, these equivalence relations could be phrased as follows. “The operator representing the event on the left along the sequence will not change whether it comes first or second. The operator representing the event on the right will shift its operational position to the left/right by the number of sites deleted/inserted before its operation, when it comes second”.^11^

Now, we will extend the binary equivalence relations, Eqs. (R5.2a-R5.2d), to the equivalence relations among more general complex indel histories, each consisting of more than two indel events. Let us consider a *global* history of *N* indel events, $\left[{\widehat{M}}_1,{\widehat{M}}_2,\dots, {\widehat{M}}_N\right]$, which begins with an “ancestral state”, *s*^*A*^(∈*S*^*II*^), and ends with a “descendant state”, *s*^*D*^(∈*S*^*II*^). Obviously, the two states must satisfy the equation:

R5.3 $$ \left\langle {s}^D\right|=\left\langle {s}^A\right|{\widehat{M}}_1{\widehat{M}}_2\cdots {\widehat{M}}_N. $$

Given an indel history, we can identify PASs unambiguously. Suppose that such PASs separate the indel events, ${\widehat{M}}_{\nu }$ (*ν* = 1, 2, …, *N*) in $\left[{\widehat{M}}_1,{\widehat{M}}_2,\dots, {\widehat{M}}_N\right]$, into *K local* subsets of indels, each of which is confined either between a pair of PASs or between a PAS and an end of the resulting PWA. Number the *K* local subsets as *k* = 1, 2, …, *K* from left to right, and let *N*_*k*_ be the number of indel events in the *k* th local subset. Naturally, ∑_*k* = 1_^*K*^*N*_*k*_ = *N*. And let ${\widehat{M}}^{\prime}\left[k,{i}_k\right]$ be the element of ${\left\{{\widehat{M}}_{\nu}\right\}}_{\nu =1,2,\dots, N}$ representing the *i*_*k*_ th event (in the temporal order) in the *k* th local subset (*i*_*k*_ = 1, 2, …, *N*_*k*_; *k* = 1, 2, …, *K*). (The prime here indicates that the operator is equivalent to the prime-less version). Then, repeatedly applying the binary equivalence relations, Eqs. (R5.2a-R5.2d), between the indel operators belonging to *different* local subsets, we can move the operators around in the product in Eq. (R5.3) and get the following equation:

R5.4 $$ \left\langle {s}^D\right|=\left\langle {s}^A\right|\left[\widehat{M}\left[K,1\right]\cdots \widehat{M}\left[K,{N}_K\right]\right]\cdots \left[\widehat{M}\left[1,1\right]\cdots \widehat{M}\left[K,{N}_1\right]\right]. $$

Here $\widehat{M}\left[k,{i}_k\right]$ is an operator that was obtained from ${\widehat{M}}^{\prime}\left[k,{i}_k\right]$ through the series of equivalence relations Eqs. (R5.2a-R5.2d) that brought Eq. (R5.3) into Eq. (R5.4). As in Eq. (R5.3), the operators in each pair of large square parentheses in Eq. (R5.4) are arranged in temporal order, so that the earliest event in each local subset will come leftmost. But it should be noted that the order among the pairs of large square parentheses is the opposite of the actual spatial order among the local subsets, so that the rightmost one along the sequence (the *K* th one here) will come leftmost. In this way, the operators in each local subset, *e.g*., $\left\{\widehat{M}\left[k,1\right],\dots, \widehat{M}\left[k,{N}_k\right]\right\}$, are exactly the same as those when the events in the subset alone struck 〈*s*^*A*^|. Thus the series of operators, $\left[\widehat{M}\left[k,1\right],\dots, \widehat{M}\left[k,{N}_k\right]\right]$, for the *k* th local subset defines the *k* th *local indel history* that was isolated from the global indel history, $\left[{\widehat{M}}_1,{\widehat{M}}_2,\dots, {\widehat{M}}_N\right]$, on *s*_*I*_ ∈ *S*.

Now, let us consider a history of *N* indel events other than $\left[{\widehat{M}}_1,{\widehat{M}}_2,\dots, {\widehat{M}}_N\right]$. If the temporal operator product of the history is shown to be equivalent to Eq. (R5.4) through a series of Eqs. (R5.2a-R5.2d), then it should also be connected to Eq. (R5.3) though another series of Eqs. (R5.2a-R5.2d). Therefore, it should be equivalent to $\left[{\widehat{M}}_1,{\widehat{M}}_2,\dots, {\widehat{M}}_N\right]$ in this sense. Hence, we can define a particular equivalence class, which is the set of all global indel histories that can be “decomposed” into the identical set of local indel histories, such as Eq. (R5.4), only through a series of Eqs. (R5.2a-R5.2d), between indel operators separated by at least a PAS. We will call it the “**local-history-set (LHS) equivalence class**”. In the equivalence class defined by a local history set (LHS), ${\left\{\left[\widehat{M}\left[k,1\right],\dots, \widehat{M}\left[k,{N}_k\right]\right]\right\}}_{k=1,\dots, K}$ (with ∑_*k* = 1_^*K*^*N*_*k*_ = *N*), on an initial sequence state *s*^*A*^ ∈ *S*^*II*^, there are $\frac{N!}{{\displaystyle {\prod}_{k=1}^K{N}_k}}$ LHS-equivalent global indel histories beginning with *s*^*A*^. Each of the global histories corresponds to a way of reordering *N* indel events while retaining the relative temporal order among *N*_*k*_ events within the *k* th local indel history (for every *k* = 1, …, *K*).

In the simplest example at the beginning of this section (Eq. (R5.1) and above), the corresponding LHS is: $\left\{\left[{\widehat{M}}_D\left(2,4\right)\right],\left[{\widehat{M}}_I\left(6,3\right)\right]\right\}$. The LHS consists of two local histories, each of which is a single-indel history (Fig. 5c). As a slightly more complex example, consider the history, $\left[{\widehat{M}}_D\left(3,3\right),{\widehat{M}}_I\left(5,2\right),{\widehat{M}}_D\left(2,3\right),{\widehat{M}}_I\left(5,1\right)\right]$, illustrated in Fig. 4. This history belongs to the LHS equivalence class represented by the LHS: $\left\{\left[{\widehat{M}}_D\left(3,3\right),{\widehat{M}}_D\left(2,3\right)\right],\left[{\widehat{M}}_I\left(6,2\right),{\widehat{M}}_I\left(8,1\right)\right]\right\}$, which consists of two 2-indel local histories. If this LHS is recast into the form in Eq. (R5.4), we have: $\widehat{M}\left[1,1\right]={\widehat{M}}_D\left(3,3\right)$, $\widehat{M}\left[1,2\right]={\widehat{M}}_D\left(2,3\right)$, $\widehat{M}\left[2,1\right]={\widehat{M}}_I\left(6,2\right)$, and $\widehat{M}\left[2,2\right]={\widehat{M}}_I\left(8,1\right)$.

### R6. Factorability of pairwise alignment probability: brief description

Now we are ready to examine the factorability of the *ab initio* probability of PWA *α*(*s*^*A*^, *s*^*D*^), *P*[(*α*(*s*^*A*^, *s*^*D*^), [*t*_*I*_, *t*_*F*_])|(*s*^*A*^, *t*_*I*_)] in Eq. (R4.9), given the ancestral state (*s*^*A*^) at the initial time (*t*_*I*_). Here the “factorability” means that the PWA probability can be re-expressed as the product of an overall factor and contributions from local regions. Natural candidates for the local regions would be those in between the PASs, because we know that indels never hit or pierced PASs (if the alignment is correct). We are not interested in trivial factorability. Thus, we only consider PWAs (or global histories) each of which requires at least two local indel histories. In the following, we will only briefly describe our demonstration of the PWA probability factorization. Its more detailed yet rather intuitive description is given in Supplementary methods SM-2 in Additional file 1. (It is complemented by mathematically rigorous arguments in Supplementary appendix SA-2 in Additional file 2).

We first notice that each component probability, $P\left[\left(\left[{\widehat{M}}_1,{\widehat{M}}_2,\cdots, {\widehat{M}}_N\right],\left[{t}_I,{t}_F\right]\right)\left|\left({s}^A,{t}_I\right)\right.\right]$ given by Eq. (R4.7), will not be factorable. This is because its domain of multiple-time integration is not a direct product. So, lets consider the total probability of a LHS equivalence class, ${\left[\overset{\rightharpoonup }{\overset{\rightharpoonup }{\widehat{M}}}\right]}_{LHS}$ (with $\overset{\rightharpoonup }{\overset{\rightharpoonup }{\widehat{M}}}$ abbreviating ${\left\{\left[\widehat{M}\left[k,1\right],\dots, \widehat{M}\left[k,{N}_k\right]\right]\right\}}_{k=1,\dots, K}$):

R6.1 $$ P\left[\left({\left[\overset{\rightharpoonup }{\overset{\rightharpoonup }{\widehat{M}}}\right]}_{LHS},\left[{t}_I,{t}_F\right]\right)\left|\left({s}^A,{t}_I\right)\right.\right]\equiv {\displaystyle \sum_{\left[{\widehat{M}}_1,{\widehat{M}}_2,\cdots, {\widehat{M}}_N\right]\in {\left[\overset{\rightharpoonup }{\overset{\rightharpoonup }{\widehat{M}}}\right]}_{LHS}}P\left[\left(\left[{\widehat{M}}_1,{\widehat{M}}_2,\cdots, {\widehat{M}}_N\right],\left[{t}_I,{t}_F\right]\right)\left|\left({s}^A,{t}_I\right)\right.\right]}. $$

We can show that this probability can be factorized as:

R6.2 $$ {\mu}_P\left[\left({\left[\overset{\rightharpoonup }{\overset{\rightharpoonup }{\widehat{M}}}\right]}_{LHS},\left[{t}_I,\;{t}_F\right]\right)\left|\left({s}^A,{t}_I\right)\right.\right]={\displaystyle \prod_{k=1}^K{\mu}_P\left[\left(\left[\widehat{M}\left[k,1\right],\dots, \widehat{M}\left[k,{N}_k\right]\right],\left[{t}_I,{t}_F\right]\right)\left|\left({s}^A,{t}_I\right)\right.\right]}, $$

where

R6.3 $$ \begin{array}{l}{\mu}_P\left[\left(\left[\widehat{M}\left[k,1\right],\dots, \widehat{M}\left[k,{N}_k\right]\right],\left[{t}_I,{t}_F\right]\right)\left|\left({s}^A,{t}_I\right)\right.\right]\\ {}\equiv P\left[\left(\left[\widehat{M}\left[k,1\right],\dots, \widehat{M}\left[k,{N}_k\right]\right],\left[{t}_I,{t}_F\right]\right)\left|\left({s}^A,{t}_I\right)\right.\right]/P\left[\left(\left[\right],\left[{t}_I,{t}_F\right]\right)\left|\left({s}^A,{t}_I\right)\right.\right],\end{array} $$

R6.4 $$ {\mu}_P\left[\left({\left[\overset{\rightharpoonup }{\overset{\rightharpoonup }{\widehat{M}}}\right]}_{LHS},\left[{t}_I,{t}_F\right]\right)\left|\left({s}^A,{t}_I\right)\right.\right]\equiv P\left[\left({\left[\overset{\rightharpoonup }{\overset{\rightharpoonup }{\widehat{M}}}\right]}_{LHS},\left[{t}_I,{t}_F\right]\right)\left|\left({s}^A,{t}_I\right)\right.\right]/P\left[\left[\left[\right],\left[{t}_I,{t}_F\right]\right]\left|\left({s}^A,{t}_I\right)\right.\right], $$

if the following two conditions are satisfied.

**Condition (i):** The rate of an indel event ($r\left({\widehat{M}}_{\nu };{s}_{\nu -1},{\tau}_{\nu}\right)$) is independent of the portion of the sequence state (*s*_*ν* − 1_) outside of the region of the local history the event (${\widehat{M}}_{\nu }$) belongs to.

**Condition (ii):** The increment of the exit rate due to an indel event (*δR*_*X*_^*ID*^(*s*_*ν*_, *s*_*ν* − 1_, *τ*)≡*R*_*X*_^*ID*^(*s*_*ν*_, *τ*) − *R*_*X*_^*ID*^(*s*_*ν* − 1_, *τ*), with $\left\langle {s}_{\nu}\right|=\left\langle {s}_{\nu -1}\right|{\widehat{M}}_{\nu }$) is independent of the portion of the sequence state (*s*_*ν* − 1_) outside of the region of the local history the event (${\widehat{M}}_{\nu }$) belongs to.

See Supplementary appendix SA-2 in Additional file 2 for the derivation of the mathematically rigorous version of this set of conditions. (For illustration, in Supplementary methods SM-3 in Additional file 1, the factorability of the probability was examined for the simplest concrete LHS equivalence class (in Fig. 5)). Condition (i) is somewhat similar to the “context-independence” condition imposed on the “long indel” model [21], though our condition is slightly less restrictive. Condition (ii) has never been found or even discussed thus far. In fact, the “long indel” model trivially satisfies this condition (see subsection R8-1), thus [21] did not need to pay attention to it. However, this condition is not always satisfied. Indeed, as exemplified in subsection R8-2, some models have non-factorable alignment probabilities due to the violation of this condition even though condition (i) is satisfied.

Each global indel history (in the set of all PWA-consistent indel histories, ${\tilde{\mathrm{H}}}^{ID}\left[\alpha \left({s}^A,{s}^D\right)\right]$) belongs to a single LHS equivalence class (say, ${\left[\overset{\rightharpoonup }{\overset{\rightharpoonup }{\widehat{M}}}\right]}_{LHS}$). And all elements of ${\left[\overset{\rightharpoonup }{\overset{\rightharpoonup }{\widehat{M}}}\right]}_{LHS}$ can result in the same PWA. Therefore, we get the direct sum structure:

R6.5 $$ {\tilde{\mathrm{H}}}^{ID}\left[\alpha \left({s}^A,{s}^D\right)\right]={\displaystyle \underset{\overset{\rightharpoonup }{\overset{\rightharpoonup }{\widehat{M}}}\in {\tilde{\Lambda}}^{ID}\left[\alpha \left({s}^A,\kern0.5em {s}^D\right)\right]}{\cup }{\left[\overset{\rightharpoonup }{\overset{\rightharpoonup }{\widehat{M}}}\right]}_{LHS}}, $$

where ${\tilde{\Lambda}}^{ID}\left[\alpha \left({s}^A,{s}^D\right)\right]$ is the set of all LHSs consistent with *α*(*s*^*A*^, *s*^*D*^). Hence, the PWA probability, Eq. (R4.9), can be rewritten as:

R6.6 $$ P\left[\left(\alpha \left({s}^A,{s}^D\right),\left[{t}_I,{t}_F\right]\right)\left|\left({s}^A,{t}_I\right)\right.\right]={\displaystyle \sum_{\overset{\rightharpoonup }{\overset{\rightharpoonup }{\widehat{M}}}\in {\tilde{\Lambda}}^{ID}\left[\alpha \left({s}^A,{s}^D\right)\right]}P\left[\left({\left[\overset{\rightharpoonup }{\overset{\rightharpoonup }{\widehat{M}}}\right]}_{LHS},\left[{t}_I,{t}_F\right]\right)\left({s}^A,{t}_I\right)\right]}. $$

We are considering *all* indel histories, including non-parsimonious ones, that can yield *α*(*s*^*A*^, *s*^*D*^). Thus, the LHSs belonging to ${\tilde{\Lambda}}^{ID}\left[\alpha \left({s}^A,{s}^D\right)\right]$ may consist of different numbers of local histories. We will choose the maximum possible set of PASs in the given PWA, which separates the PWA into the finest potentially local-history-accommodating regions, denoted as ${\gamma}_1,{\gamma}_2,\dots, {\gamma}_{\kappa_{max}}$. (*κ*_*max*_ is uniquely determined by the PWA and the evolutionary model).^12^ Then, we can represent any $\overset{\rightharpoonup }{\overset{\rightharpoonup }{\widehat{M}}}={\left\{\left[\widehat{M}\left[k,1\right],\dots, \widehat{M}\left[k,{N}_k\right]\right]\right\}}_{k=1,\dots, K}\in {\tilde{\Lambda}}^{ID}\left[\alpha \left({s}^A,{s}^D\right)\right]$ as a vector with *κ*_*max*_ components: $\overset{\rightharpoonup }{\overset{\rightharpoonup }{\widehat{M}}}=\left(\overset{\rightharpoonup }{\widehat{M}}\left[{\gamma}_1\right],\overset{\rightharpoonup }{\widehat{M}}\left[{\gamma}_2\right],\dots, \overset{\rightharpoonup }{\widehat{M}}\left[{\gamma}_{\kappa_{max}}\right]\right)$. (See Figure S1 in Additional file 1). Substituting Eqs. (R6.2,R6.4) into Eq. (R6.6), and exploiting the vector representation of the LHSs, we can reach the desired expression:

R6.7 $$ \begin{array}{l}P\left[\left(\alpha \left({s}^A,{s}^D\right),\left[{t}_I,{t}_F\right]\right)\left|\left({s}^A,{t}_I\right)\right.\right]\\ {}=P\left[\left(\left[\right],\left[{t}_I,{t}_F\right]\right)\left|\left({s}^A,{t}_I\right)\right.\right]\times {\displaystyle \prod_{\kappa =1}^{\kappa_{\max }}{\tilde{\mu}}_P\left[\left({\tilde{\Lambda}}^{ID}\left[{\gamma}_{\kappa };\alpha \left({s}^A,{s}^D\right)\right],\left[{t}_I,{t}_F\right]\right)\left|\left({s}^A,{t}_I\right)\right.\right]}.\end{array} $$

Here, ${\tilde{\Lambda}}^{ID}\left[{\gamma}_{\kappa };\alpha \left({s}^A,{s}^D\right)\right]$ denotes the set of local indel histories that can give rise to the sub-PWA of *α*(*s*^*A*^, *s*^*D*^) confined in *γ*_*κ*_, and the multiplication factor,

R6.8 $$ {\tilde{\mu}}_P\left[\left({\tilde{\Lambda}}^{ID}\left[{\gamma}_{\kappa };\alpha \left({s}^A,{s}^D\right)\right],\left[{t}_I,{t}_F\right]\right)\left|\left({s}^A,{t}_I\right)\right.\right]\equiv {\displaystyle \sum_{\overset{\rightharpoonup }{\widehat{M}}\left[{\gamma}_{\kappa}\right]\in {\tilde{\Lambda}}^{ID}\left[{\gamma}_{\kappa };\alpha \left({s}^A,{s}^D\right)\right]}{\mu}_P\left[\left(\overset{\rightharpoonup }{\widehat{M}}\left[{\gamma}_{\kappa}\right],\left[{t}_I,{t}_F\right]\right)\left|\left({s}^A,{t}_I\right)\right.\right]}, $$

represents the total contribution from ${\tilde{\Lambda}}^{ID}\left[{\gamma}_{\kappa };\alpha \left({s}^A,{s}^D\right)\right]$ to the PWA probability. (Here *μ*_*P*_[([], [*t*_*I*_, *t*_*F*_])|(*s*^*A*^, *t*_*I*_)] = 1 should be remembered).

Equation (R6.7) states that the PWA probability is factorized into the product of an overall factor (*P*[([], [*t*_*I*_, *t*_*F*_])|(*s*^*A*^, *t*_*I*_)]) and contributions from regions accommodating local indel histories (${\tilde{\mu}}_P\left[\left({\tilde{\Lambda}}^{ID}\left[{\gamma}_{\kappa };\alpha \left({s}^A,{s}^D\right)\right],\left[{t}_I,{t}_F\right]\right)\left|\left({s}^A,{t}_I\right)\right.\right]$’s). Therefore, the set of conditions, (i) and (ii), is sufficient for the factorability of the PWA probability. At present, we are not sure whether the set of conditions is also necessary or not. This may not be the case in the *rigorous* sense, and there may be some instances with factorable PWA probabilities despite the violation of condition (i) or (ii). Nevertheless, even if there are, we suspect that such cases should be isolated, requiring intricate cancellations of the terms. Thus, we will refer to the conditions (i) and (ii) as the “sufficient and nearly necessary set of conditions” for factorable PWA probabilities.

### R7. Factorability of multiple sequence alignment probability: brief description

Thus far, we only examined the probability of a given PWA, conditioned on an ancestral state at initial time. Actually, once we know how to calculate such conditional PWA probabilities, we can build them up along the phylogenetic tree to calculate the probability of a given MSA, as described in the introductions of [13] and [14]. (See also [36] for an essentially equivalent method that appears different.) Here, we basically follow their procedures. However, it should be stressed that the MSA probability here will be calculated *ab initio* under a genuine evolutionary stochastic model and not under a HMM or a transducer, which is not necessarily evolutionarily consistent. This section briefly explains the derivation of the factorization of an *ab initio* MSA probability. For details on the derivation, see Supplementary methods SM-4 in Additional file 1.

In this section, we formally calculate the *ab initio* probability of a MSA given a rooted phylogenetic tree, *T* = ({*n*}_*T*_, {*b*}_*T*_), where {*n*}_*T*_ is the set of all nodes of the tree, and {*b*}_*T*_ is the set of all branches of the tree. We decompose the set of all nodes as: {*n*}_*T*_ = *Ν*^*IN*^(*T*) + *Ν*^*X*^(*T*), where *Ν*^*IN*^(*T*) is the set of all internal nodes and ${N}^X(T)=\left\{{n}_1,\dots, {n}_{N^X}\right\}$ is the set of all external nodes. (The *N*^*X*^≡|*Ν*^*X*^(*T*)| is the number of external nodes.) The root node plays an important role and will be denoted as *n*^*Root*^. Because the tree is rooted, each branch *b* is directed. Thus, let *n*^*A*^(*b*) denote the “ancestral node” on the upstream end of *b*, and let *n*^*D*^(*b*) denote the “descendant node” on the downstream end of *b*. Let *s*(*n*) ∈ *S*^*II*^ be a sequence state at the node *n* ∈ {*n*}_*T*_. Especially, we use abbreviations: *s*^*A*^(*b*)≡*s*(*n*^*A*^(*b*)) ∈ *S*^*II*^ and *s*^*D*^(*b*)≡*s*(*n*^*D*^(*b*)) ∈ *S*^*II*^. Finally, as mentioned in Background, we suppose that the branch lengths, {|*b*||*b* ∈ {*b*}_*T*_}, and the indel model parameters, {*Θ*_*ID*_(*b*)}_*T*_≡{*Θ*_*ID*_(*b*)|*b* ∈ {*b*}_*T*_}, are all given. Note that the model parameters *Θ*_*ID*_(*b*) could depend on the branch, at least theoretically.

First, we extend the ideas proposed in [13, 14, 36] to each indel history along a tree, by regarding the indel history along a branch as a map (or a transformation) from the ancestral state to the descendant state, as follows. An indel history along a tree consists of indel histories along all branches of the tree that are interdependent, in the sense that the indel process of a branch *b* determines a sequence state *s*^*D*^(*b*) at its descendant node *n*^*D*^(*b*), on which the indel processes along its downstream branches depend. Thus, an indel history on a given root sequence state *s*^*Root*^ = *s*(*n*^*Root*^) ∈ *S*^*II*^ automatically determines the sequence states at all nodes, {*s*(*n*) ∈ *S*^*II*^*for*^∀^*n* ∈ {*n*}_*T*_}. Let ${\tilde{\mathrm{H}}}^{ID}\left({s}_0\right)\equiv {\displaystyle {\cup}_{N=0}^{\infty }{\mathrm{H}}^{ID}\left(N;{s}_0\right)}$ (with Η^*ID*^(*N*; *s*_0_) defined below Eq. (R4.6)) be the set of all indel histories along a time axis (or a branch) starting with state *s*_0_. Then, each indel history, ${\left\{\overset{\rightharpoonup }{\widehat{M}}(b)\right\}}_T$, along tree *T* and starting with *s*^*Root*^ can be specifically expressed as:

R7.1 $$ \left\{{}_{\left\langle {s}^D(b)\right|=\left\langle {s}^A(b)\right|\widehat{M}{}_1(b)\cdots \widehat{M}{}_{N(b)}(b)\;for\;{}^{\forall }b\in {\left\{b\right\}}_T}^{\overset{\rightharpoonup }{\widehat{M}}(b)=\left[\widehat{M}{}_1(b),\dots, \widehat{M}{}_{N(b)}(b)\right]\in {\tilde{\mathrm{H}}}^{ID}\left({s}^A(b)\right)\; and}\left|{}^{s\left({n}^{Root}\right)={s}^{Root}}\right.\right\}. $$

Here, the symbol, ${\widehat{M}}_{\nu }(b)$, denotes the *ν* th event in the indel history along branch *b* ∈ {*b*}_*T*_. The probability of the indel history, Eq. (R7.1), can be calculated as:

R7.2 $$ P\left[{\left\{\overset{\rightharpoonup }{\widehat{M}}(b)\right\}}_T\left|\left({s}^{Root},{n}^{Root}\right)\right.\right]=\left({\displaystyle \prod_{b\in {\left\{b\right\}}_T}P\left[\left(\overset{\rightharpoonup }{\widehat{M}}(b),b\right)\left|\left({s}^A(b),{n}^A(b)\right)\right.\right]}\right)\left|{}_{\begin{array}{l}s\left({n}^{Root}\right)={s}^{Root},\\ {}\left\langle {s}^D(b)\right|=\left\langle {s}^A(b)\right|{\widehat{M}}_1(b)\cdots {\widehat{M}}_{N(b)}(b)\\ {}for\;{}^{\forall }b\in {\left\{b\right\}}_T\end{array}}\right.. $$

Here, the probability of an indel history, $\overset{\rightharpoonup }{\widehat{M}}(b)=\left[\widehat{M}{}_1(b),\dots, \widehat{M}{}_{N(b)}(b)\right]\in {\tilde{\mathrm{H}}}^{ID}\left({s}^A(b)\right)$, along branch *b* ∈ {*b*}_*T*_ is given by the probability during the corresponding time interval, [*t*(*n*^*A*^(*b*)), *t*(*n*^*D*^(*b*))]:

R7.3 $$ \begin{array}{l}P\left[\left(\overset{\rightharpoonup }{\widehat{M}}(b),b\right)\left|\left({s}^A(b),{n}^A(b)\right)\right.\right]\\ {}\equiv P\left[\left(\left[{\widehat{M}}_1(b),\cdots, {\widehat{M}}_{N(b)}(b)\right],\left[t\left({n}^A(b)\right),t\left({n}^D(b)\right)\right]\right)\left|\left({s}^A(b),t\left({n}^A(b)\right)\right)\right.\right]\left|{\varTheta}_{ID}(b)\right..\end{array} $$

Here we explicitly showed the branch-dependence of the model parameters.

Now, consider a MSA, $\alpha \left[{s}_1,{s}_2,\dots, {s}_{N^X}\right]$, among the sequences at the external nodes, *s*_*i*_ = *s*(*n*_*i*_) ∈ *S*^*II*^ (*n*_*i*_ ∈ *Ν*^*X*^(*T*)). (Remember that the term “MSA” here means its homology structure, as noted in endnote (10)). Let $\left({s}^{Root},{\left\{\overset{\rightharpoonup }{\widehat{M}}(b)\right\}}_T\right)$ be a pair of a root state and an indel history along *T* starting with the state. And let ${\tilde{\Psi}}^{ID}\left[\alpha \left[{s}_1,{s}_2,\dots, {s}_{N^X}\right];T\right]$ be the set of all such pairs defined on *T* consistent with $\alpha \left[{s}_1,{s}_2,\dots, {s}_{N^X}\right]$. Then, analogously to Eq. (R4.9) supplemented with Eq. (R4.7) for a PWA, the probability of a given MSA under a given model setting (including *T*) should be expressed as:

R7.4 $$ P\left[\alpha \left[{s}_1,{s}_2,\dots, {s}_{N^X}\right]\Big|T\right]={\displaystyle \sum_{\begin{array}{l}\kern2em \left({s}^{Root},\kern0.5em {\left\{\overset{\rightharpoonup }{\widehat{M}}(b)\right\}}_T\right)\\ {}\in {\tilde{\Psi}}^{ID}\left[\alpha \left[{s}_1,{s}_2,\dots, {s}_{N^X}\right];\kern0.5em T\right]\end{array}}P\left[\left({s}^{Root},{n}^{Root}\right)\right]P\left[{\left\{\overset{\rightharpoonup }{\widehat{M}}(b)\right\}}_T\left|\left({s}^{Root},{n}^{Root}\right)\right.\right]}, $$

which is supplemented with Eq. (R7.2). Here, *P*[(*s*^*Root*^, *n*^*Root*^)] is the probability of state *s*^*Root*^ at the root node. (It may be interpreted as the prior in a Bayesian formalism.) If you will, Eqs. (R7.4) and (R7.2) could be considered as the “perturbation expansion” of an *ab initio* MSA probability. To make this formal expansion more tractable, let ${\left\{s(n)\right\}}_{N^{IN}}\equiv \left\{s(n)\in S\left|n\in {N}^{IN}(T)\right.\right\}$ denote a set of ancestral states at all internal nodes (, or, more precisely, its equivalence class in the sense of endnote (8)). To be consistent with a given MSA, the ancestral states must satisfy the “phylogenetic correctness” condition in each MSA column (*e.g*., [37, 38]).^13^ Let $\Sigma \left[\alpha \left[{s}_1,{s}_2,\dots, {s}_{N^X}\right];\left\{n\in {N}^{IN}(T)\right\};T\right]$ be the set of all ${\left\{s(n)\right\}}_{N^{IN}}$’s consistent with $\alpha \left[{s}_1,{s}_2,\dots, {s}_{N^X}\right]$ (and tree *T*). Then, Eq. (R7.4) supplemented with Eq. (R7.2) can be rewritten as:

R7.5 $$ P\left[\alpha \left[{s}_1,{s}_2,\dots, {s}_{N^X}\right]\left|T\right.\right]={\displaystyle \sum_{\begin{array}{l}\kern6em {\left\{s(n)\right\}}_{N^{IN}}\\ {}\in \Sigma \left[\alpha \left[{s}_1,{s}_2,\dots, {s}_{N^X}\right];\left\{n\in {\mathrm{N}}^{IN}(T)\right\};T\right]\end{array}}P\left[\alpha \left[{s}_1,{s}_2,\dots, {s}_{N^X}\right];{\left\{s(n)\right\}}_{N^{IN}}\left|T\right.\right]}. $$

Here, $P\left[\alpha \left[{s}_1,{s}_2,\dots, {s}_{N^X}\right];{\left\{s(n)\right\}}_{N^{IN}}\left|T\right.\right]$ is the probability of simultaneously getting $\alpha \left[{s}_1,{s}_2,\dots, {s}_{N^X}\right]$ and ${\left\{s(n)\right\}}_{N^{IN}}$. This probability is the sum of contributions from all indel histories sharing the same homology structure among sequence states at all nodes. Especially, the sequence states at internal nodes have homology structures (with the states at other nodes) fixed for respective nodes. Thus, through some reasoning (explained in SM-4), we get:

R7.6 $$ \begin{array}{l}P\left[\alpha \left[{s}_1,{s}_2,\dots, {s}_{N^X}\right];{\left\{s(n)\right\}}_{{\mathrm{N}}^{IN}}\left|T\right.\right]\\ {}=P\left[\left({s}^{Root},{n}^{Root}\right)\right]{\displaystyle \prod_{b\in {\left\{b\right\}}_T}P\left[\left(\alpha \left({s}^A(b),{s}^D(b)\right),b\right)\left|\left({s}^A(b),{n}^A(b)\right)\right.\right]}.\end{array} $$

Here,

R7.7 $$ \begin{array}{l}P\left[\left(\alpha \left({s}^A(b),{s}^D(b)\right),b\right)\left|\left({s}^A(b),{n}^A(b)\right)\right.\right]\\ {}\equiv P\left[\left(\alpha \left({s}^A(b),{s}^D(b)\right),\left[t\left({n}^A(b)\right),t\left({n}^D(b)\right)\right]\right)\left|\left({s}^A(b),t\left({n}^A(b)\right)\right)\right.\right]\left|{\varTheta}_{ID}(b)\right.\end{array} $$

is the probability of the ancestor-descendant PWA along branch *b*. This Eq. (R7.6) is basically the expression proposed in [13, 14], and we demonstrated in effect that their proposal also holds even with a genuine stochastic evolutionary model. Usually, Eq. (R7.5) supplemented with Eq. (R7.6) is much more tractable than Eq. (R7.4) supplemented with Eq. (R7.2), because of the two reasons. (1) Usually, it is not the indel history along the tree but (the homology structure of) the set of ancestral sequence states that is inferred from a given MSA. (2) The probability of each indel history along the tree (Eq. (R7.2)) is not factorable in general, whereas Eq. (R7.6) is a product of PWA probabilities, each of which should be factorable if the conditions (i) and (ii) in section R6 are satisfied.

Now, we can show that, if the “condition (iii)” given below in addition to conditions (i) and (ii) is satisfied, we can factorize the MSA probability into a form somewhat similar to Eq. (R6.7) for the PWA probability. In subsection 4.2 of [32], we demonstrated it using the history-based expansion of the MSA probability (*i.e*., Eq. (R7.4) supplemented with Eq. (R7.2)). In Supplementary methods SM-4, we will use the ancestral-state-based expansion (*i.e.*, Eq. (R7.5) supplemented with Eq. (R7.6)), as was only briefly sketched at the bottom of subsection 4.2 of *(ibid*.). In a MSA, gapless columns play almost the same role as PASs in a PWA. Because of the aforementioned “phylogenetic correctness” condition, a gapless column indicates that no indel event hit or pierced the site. Therefore, gapless columns will partition a MSA into regions each of which accommodates a local subset of every global history. Analogously to the argument above Eq. (R6.7), let ${C}_1,{C}_2,\dots, {C}_{K_{max}}$ be the maximum possible set of such regions determined by a given MSA and a model setting (including the tree) (Figure S2 in Additional file 1). (As argued in subsection R8-3, all gapless columns are not necessarily needed to delimit the regions.) Because the summation in Eq. (R7.5) involves the summation over all MSA-consistent root states, it would be convenient to specify a “reference” root state, *s*_0_^*Root*^. It can be anything, as long as it is the state at the root consistent with $\alpha \left[{s}_1,{s}_2,\dots, {s}_{N^X}\right]$. Technically, one good candidate for *s*_0_^*Root*^ would be a root state obtained by applying the Dollo parsimony principle [39] to each column of the MSA, because it is arguably the most readily available state that satisfies the phylogenetic correctness condition along the entire MSA. Then, we will impose the following condition.

**Condition (iii):**

R7.8 $$ P\left[\left({s}^{Root},{n}^{Root}\right)\right]=P\left[\left({s}_0^{Root},{n}^{Root}\right)\right]{\displaystyle \prod_{\mathrm{K}=1}^{{\mathrm{K}}_{\max }}{\mu}_P\left[{s}^{Root},{s}_0^{Root},{n}^{Root};{C}_K\right]}. $$

Here the multiplication factor, *μ*_*P*_[*s*^*Root*^, *s*_0_^*Root*^, *n*^*Root*^; *C*_*Κ*_], represents the change in the state probability at the root due to the difference between *s*^*Root*^ and *s*_0_^*Root*^ within *C*_*Κ*_. This equation holds, *e.g*., when *P*[(*s*^*Root*^, *n*^*Root*^)] is a geometric distribution or a uniform distribution of the root sequence length.^14^

Under the conditions (i), (ii) and (iii), through a series of formal calculations and reasoning, Eq. (R7.5) supplemented with Eq. (R7.6) can be re-expressed into the final factorized form:

R7.9 $$ P\left[\alpha \left[{s}_1,{s}_2,\dots, {s}_{N^X}\right]\left|T\right.\right]={P}_0\left[{s}_0^{Root}\left|T\right.\right]{\displaystyle \prod_{\mathrm{K}=1}^{{\mathrm{K}}_{\max }}{\tilde{\overset{\smile }{M}}}_P\left[\alpha \left[{s}_1,{s}_2,\dots, {s}_{N^X}\right];{s}_0^{Root};{C}_K\left|T\right.\right]}. $$

Here,

R7.10 $$ {P}_0\left[{s}_0^{Root}\left|T\right.\right]\equiv P\left[\left({s}_0^{Root},{n}^{Root}\right)\right]P\left[{\left\{\left[\right]\right\}}_T\left|\left({s}_0^{Root},{n}^{Root}\right)\right.\right] $$

is the probability of having state *s*_0_^*Root*^ that has been intact all across tree *T*, and ${\tilde{\overset{\smile }{M}}}_P\left[\alpha \left[{s}_1,{s}_2,\dots, {s}_{N^X}\right];{s}_0^{Root};{C}_K\left|T\right.\right]$ is the multiplication factor contributed from all local indel histories (along *T*) confined in *C*_*Κ*_.^15^ Briefly, the multiplication factor is the summation of terms over all possible sets of the MSA-consistent ancestral states in *C*_*Κ*_. And each of the terms is the product of the local PWA multiplication factors (Eq. R6.8) confined in *C*_*Κ*_ (Figure S2 in Additional file 1), the exponential of minus the summation over all *b*’s of the time-integrated exit rate differences between *s*^*A*^(*b*) and *s*_0_^*Root*^ coming from *C*_*Κ*_, and *μ*_*P*_[*s*^*Root*^, *s*_0_^*Root*^, *n*^*Root*^; *C*_*Κ*_] for the root state probability. (For the factor’s exact expression and the detailed derivation of Eq. (R7.9), see Supplementary methods SM-4 in Additional file 1).

### R8. Examples: Models with factorable/non-factorable alignment probabilities

A merit of conditions (i) and (ii) given in section R6 is that they can draw the line between evolutionary models with factorable PWA probabilities and those with non-factorable ones. To illustrate the use of these conditions, we here give three examples: (1) a simple model with factorable probabilities, (2) a simple model with non-factorable probabilities, and (3) a non-trivial model with factorable probabilities. (For more examples, see section 5 of [32]).

#### R8-1. Totally space-homogeneous model

The simplest conceivable indel models would be those whose indel rate parameters are space-homogeneous, *i.e.*, independent of the positions where the indels occur:

R8-1.1 $$ {r}_I\left(x,{l}_1;s,t\right)={g}_I\left({l}_1,t\right), $$

R8-1.2 $$ {r}_D\left({x}_B,{x}_B+{l}_2-1;s,t\right)={g}_D\left({l}_2,t\right). $$

In fully space-homogeneous models, these equations hold for 1 ≤ *x* ≤ *L*(*s*) − 1, 1 ≤ *l*_1_ ≤ *L*_*I*_^*CO*^, 1 ≤ *l*_2_ ≤ *L*_*D*_^*CO*^, and 2 − *l*_2_ ≤ *x*_*B*_ ≤ *L*(*s*), where *L*_*I*_^*CO*^ and *L*_*D*_^*CO*^ are the “cut-off lengths” for insertions and deletions, respectively. (Depending on the model, *r*_*I*_(0, *l*; *s*, *t*) = *g*_*I*;*L*_(*l*, *t*) and *r*_*I*_(*L*(*s*), *l*; *s*, *t*) = *g*_*I*;*R*_(*l*, *t*) could differ from *g*_*I*_(*l*, *t*) in Eq. (R8-1.1)). In fact, these conditions were imposed in nearly all continuous-time Markov models of indels that were studied in the past. Note that the rate parameters in Eqs. (R8-1.1,R8-1.2) could depend on time, although most studies thus far assumed that the rates are time-independent as well. Eqs. (R8-1.1,R8-1.2) automatically guarantees condition (i). Thus, all we have to do is check whether or not condition (ii) is also satisfied. Indeed, we can show it is. The exit rate of this model is calculated by substituting Eqs. (R8-1.1,R8-1.2) into Eq. (R4.3) (supplemented with Eqs. (R3.14,R3.15)), and we find that it is an affine function of the sequence length (*L*(*s*)):

R8-1.3 $$ {R}_X^{ID}\left(s,t\right)=A(t)L(s)+B(t), $$

with $A(t)={\displaystyle {\sum}_{l=1}^{L_I^{CO}}{g}_I\left(l,t\right)}+{\displaystyle {\sum}_{l=1}^{L_D^{CO}}{g}_D\left(l,t\right)}$ and $B(t)={\displaystyle {\sum}_{l=1}^{L_D^{CO}}\left(l-1\right){g}_D\left(l,t\right)}-{\displaystyle {\sum}_{l=1}^{L_I^{CO}}{g}_I\left(l,t\right)}+{\displaystyle {\sum}_{l=1}^{L_I^{CO}}\left({g}_{I;L}\left(l,t\right)+{g}_{I;R}\left(l,t\right)\right)}$. If the exit rate is affine, we have, for $\left\langle s\left(\nu \right)\right|=\left\langle s\left(\nu -1\right)\right|{\widehat{M}}_{\nu }$:

R8-1.4 $$ \begin{array}{l}\delta {R}_X^{ID}\left(s\left(\nu \right),s\left(\nu -1\right),t\right)\equiv {R}_X^{ID}\left(s\left(\nu \right),t\right)-{R}_X^{ID}\left(s\left(\nu -1\right),t\right)\\ {}=A(t)\left[L\left(s\left(\nu \right)\right)-L\left(s\left(\nu -1\right)\right)\right]=A(t)\delta L\left({\widehat{M}}_{\nu}\right).\end{array} $$

Here $\delta L\left({\widehat{M}}_{\nu}\right)$ is the length change caused by the event, ${\widehat{M}}_{\nu }$. The rightmost hand side of this equation depends only on ${\widehat{M}}_{\nu }$ and the time it occurred, but not on the sequence state (*s*(*ν* − 1)). Thus, condition (ii) is always satisfied under fully space-homogenous models, which means that alignment probabilities calculated *ab initio* (as in section R4) under such models are factorable, as shown in section R6.

An important special case of the space-homogeneous model is the model used by Dawg [26], whose indel rate parameters are given as: *g*_*I*_(*l*, *t*) = *g*_*I*;*L*_(*l*, *t*) = *g*_*I*;*R*_(*l*, *t*) = *λ*_*I*_*f*_*I*_(*l*) and *g*_*D*_(*l*, *t*) = *λ*_*D*_*f*_*D*_(*l*)*.* Because this is a special case of Eqs. (R8-1.1,R8-1.2), it naturally provides factorable alignment probabilities. This model is probably among the most flexible indel evolutionary models used thus far. The model accommodates any distributions of indel lengths (*f*_*I*_(*l*) and *f*_*D*_(*l*)) that are independent of each other, and independent total rates for insertions and deletions (*λ*_*I*_ and *λ*_*D*_). Some of our studies [40, 41] are mostly based on this model.

Another important special case is the “long indel” model [21], whose (time-independent) rate parameters are given by: *g*_*I*_(*l*, *t*) = *λ*_*l*_, ${g}_{I;L}\left(l,t\right)={g}_{I;R}\left(l,t\right)={\tilde{\lambda}}_l^{(end)}$ (if *L*(*s*) > 0), ${g}_{I;L}\left(l,t\right)={\tilde{\tilde{\lambda}}}_l^{(whole)}$ (if *L*(*s*) = 0), and *g*_*D*_(*l*, *t*) = *μ*_*l*_. This model is less flexible than Dawg’s model, because its indel rates are subject to the detailed-balance conditions: *λ*_*l*_ = (*λ*_1_/*μ*_1_)^*l*^*μ*_*l*_, ${\tilde{\lambda}}_l^{(end)}={\left({\lambda}_1/{\mu}_1\right)}^l{\displaystyle {\sum}_{l^{\prime }=l}^{L_D^{CO}}{\mu}_{l^{\prime }}}$, and ${\tilde{\tilde{\lambda}}}_l^{(whole)}={\left({\lambda}_1/{\mu}_1\right)}^l{\displaystyle {\sum}_{l^{\prime }=l}^{L_D^{CO}}\left({l}^{\prime }-1+1\right){\mu}_{l^{\prime }}}$. Like Dawg’s model, this model is a special case of the model defined by Eqs. (R8-1.1,R8-1.2). Thus, the alignment probabilities calculated under it are indeed factorable, as verbally justified in [21]. Indeed, we can explicitly show that, as far as each LHS equivalence class is concerned, the indel component of its probability calculated according to the recipe of [21] equals the product of *P*[([], [*t*_*I*_, *t*_*F*_])|(*s*^*A*^, *t*_*I*_)] and Eq. (R6.2), *i.e.*, the “total probability” of the LHS equivalence class via our *ab initio* formulation, calculated with the aforementioned indel rate parameters. The proof is given in Supplementary appendix SA-3 in Additional file 2. It should be stressed that, although [21] ignored condition (ii), this caused no problem thanks to Eq. (R8-1.4) satisfied by any fully space-homogeneous models. Actually, it is this condition (ii) that guarantees the equivalence of the probabilities calculated via the two methods, because it equates each increment of the exit rate of a chop-zone with that of an entire sequence. The equivalence can be extended to between PWA probabilities, *provided that the contributing local indel histories are correctly enumerated.* (We are uncertain about whether this extended equivalence indeed holds, because [21] did not explicitly describe how the local indel histories were enumerated).

Regarding the insertion rates, we could somewhat relax the space-homogeneity without compromising the factorability of alignment probabilities. For example, the insertion rates could depend on the ancestries, *υ*(*s*, *x*) and *υ*(*s*, *x* + 1), of sites flanking the event:

R8-1.5 $$ {r}_I\left(x,l;s,t\right)={g}_I\left(\upsilon \left(s,x\right),\upsilon \left(s,x+1\right),l,t\right). $$

These rates satisfy condition (i). Eq. (R8-1.5) combined with the space-homogeneous deletion rates, Eq. (R8-1.2), still gives an exit rate whose increment due to an indel event depends only on the inserted/deleted sub-sequence (and flanking sites) but not on the regions separated from it by at least a PAS. Hence the model also satisfies condition (ii), thus providing factorable alignment probabilities. Relaxing the space-homogeneity of deletion rates, however, is somewhat difficult, particularly because of condition (ii). In subsection R8-3, we will attempt it.

#### R8-2. Space-homogeneous model flanked by biologically essential sites/regions

The space-homogeneous models discussed above may decently approximate the evolution of a sequence region under no selective pressure. A real genome, however, is scattered with regions and sites under strong or weak purifying selection. Here, we consider one of the simplest such cases, in which biologically essential sites or regions flank a neutrally evolving region from both sides.^16^ The insertion rates of this model are given by Eq. (R8-1.1) with the same domain, and the deletion rates are:

R8-2.1 $$ {r}_D\left({x}_B,{x}_E;s,t\right)=\left\{\begin{array}{l}{g}_D\left({x}_E-{x}_B+1,\kern0.5em t\right)\kern1em for\kern1em 1\le {x}_B\le {x}_E\le L(s)\kern1em  and\kern1em 1\le {x}_E-{x}_B+1\le {L}_D^{CO},\\ {}0\kern3em for\kern1em {x}_B\le 0,\kern1em {x}_E>L(s)\kern1em  or\kern1em {x}_E-{x}_B+1>{L}_D^{CO}.\end{array}\right. $$

The exit rate for this model is calculated as:

R8-2.2 $$ {R}_X^{ID}\left(s,t\right)=\left(L(s)-1\right){\displaystyle \sum_{l=1}^{L_I^{CO}}{g}_I\left(l,t\right)}+{\displaystyle \sum_{l=1}^{L_I^{CO}}\left({g}_{I;L}\left(l,t\right)+{g}_{I;R}\left(l,t\right)\right)}+{\displaystyle \sum_{l=1}^{min\left\{L(s),\kern0.5em {L}_D^{CO}\right\}}\left(L(s)-l+1\left){g}_D\right(l,t\right)}. $$

For *L*(*s*) ≥ *L*_*D*_^*CO*^, this is affine, and given by Eq. (R8-1.3) with exactly the same *A*(*t*) as before and $B(t)=-{\displaystyle {\sum}_{l=1}^{L_D^{CO}}\left(l-1\right){g}_D\left(l,t\right)}-{\displaystyle {\sum}_{l=1}^{L_I^{CO}}{g}_I\left(l,t\right)}+{\displaystyle {\sum}_{l=1}^{L_I^{CO}}\left({g}_{I;L}\left(l,t\right)+{g}_{I;R}\left(l,t\right)\right)}$. Therefore, with such a sequence length, the alignment probability is still factorable even under this model. For *L*(*s*) < *L*_*D*_^*CO*^, in contrast, it exhibits a *non-affine* form:

R8-2.3 $$ {R}_X^{ID}\left(s,t\right)=\left(L(s)-1\right){\displaystyle \sum_{l=1}^{L_I^{CO}}{g}_I\left(l,t\right)}+{\displaystyle \sum_{l=1}^{L_I^{CO}}\left({g}_{I;L}\left(l,t\right)+{g}_{I;R}\left(l,t\right)\right)}+{\displaystyle \sum_{l=1}^{L(s)}\left(L(s)-l+1\left){g}_D\right(l,t\right)}. $$

Thus, in this case, condition (ii) will not be satisfied in general, whereas condition (i) is satisfied. This case gives the simplest example of a model with non-factorable PWA probabilities despite space-homogeneous rates of indels (as long as they are allowed). In a model with non-factorable alignment probabilities, the “difference of exit rate differences”:

R8-2.4 $$ \delta \delta {R}_X^{ID}\left({s}^{\prime \prime \prime },{s}^{\prime \prime },{s}^{\prime },,,s,t\right)\equiv \delta {R}_X^{ID}\left({s}^{\prime \prime \prime },{s}^{\prime \prime },,,t\right)-\delta {R}_X^{ID}\left({s}^{\prime },s,t\right), $$

where $\left\langle {s}^{\prime}\right|=\left\langle s\right|{\widehat{M}}_{\nu_1}$, $\left\langle {s}^{\prime \prime}\right|=\left\langle s\right|{\widehat{M}}_{\nu_2}$ and $\left\langle {s}^{\prime \prime \prime}\right|=\left\langle s\right|{\widehat{M}}_{\nu_2}{\widehat{M}}_{\nu_1}$, indicates the “degree of non-factorability” due to the pair of events, ${\widehat{M}}_{\nu_1}$ and ${\widehat{M}}_{\nu_2}$, that belong to different local histories. (See the argument around Eq. (5.2.6) of [32] for more details.)

#### R8-3. Model with rate-heterogeneity across regions

It is not only space-homogenous models but also some space-heterogeneous models that satisfy both conditions (i) and (ii), albeit partially. Here we give an example. We first define a set of non-overlapping regions, Ε_*y*_(*s*_*I*_)≡[*x*_*B*;*y*_^0^, *x*_*E*;*y*_^0^] (with *y* = 1, …, *Y*), that existed in the initial state, *s*_*I*_ ∈ *S*^*II*^. We define the “descendant region”, Ε_*y*_(*s*), of Ε_*y*_(*s*_*I*_) in a descendant state (*s*) by the closed interval, Ε_*y*_(*s*)≡[*x*_*B*;*y*_(*s*), *x*_*E*;*y*_(*s*)], where *x*_*B*;*y*_(*s*) and *x*_*E*;*y*_(*s*) are the leftmost and the rightmost sites, respectively, among those descended from the sites in Ε_*y*_(*s*_*I*_). Then, based on them, we define an indel model whose rate parameters are given by:

R8-3.1 $$ {r}_I\left(x,l;s,t\right)={r}_{I; Base}\left(x,l;s,t\right)+{\displaystyle {\sum}_{y=1}^Y\Delta {r}_I\left[{E}_y\right]\left(x,l;s,t\right)}. $$

R8-3.2 $$ {r}_D\left({x}_B,{x}_E;s,t\right)={r}_{D; Base}\left({x}_B,{x}_E;s,t\right)+{\displaystyle {\sum}_{y=1}^Y\Delta {r}_D\left[{E}_y\right]\left({x}_B,{x}_E;s,t\right)}. $$

Here, the “baseline” indel rates, {*r*_*I*;*Base*_(*x*, *l*; *s*, *t*)}_*x*,*l*_ and ${\left\{{r}_{D; Base}\left({x}_B,{x}_E;s,t\right)\right\}}_{x_B,{x}_E}$, are given by Eq. (R8-1.5) and Eq. (R8-1.2), respectively. The region-specific increments, {Δ*r*_*I*_[Ε_*y*_](*x*, *l*; *s*, *t*)}_*x*,*l*_ and ${\left\{\Delta {r}_D\left[{\mathrm{E}}_y\right]\left({x}_B,{x}_E;s,t\right)\right\}}_{x_B,{x}_E}$, can be non-zero *only within* Ε_*y*_(*s*)≡[*x*_*B*;*y*_(*s*), *x*_*E*;*y*_(*s*)] defined above (panel a of Figure S3 in Additional file 1). Moreover, the increments can depend only on the portion of the sequence state within Ε_*y*_(*s*). The increments can be negative, as long as the entire rates, Eqs. (R8-3.1,R8-3.2), are non-negative. From Eqs. (R8-3.1,R8-3.2), the exit rates can be decomposed as:

R8-3.3 $$ {R}_X^{ID}\left(s,t\right)={R}_{X; Base}^{ID}\left(s,t\right)+{\displaystyle \sum_{y=1}^Y\Delta {R}_X^{ID}\left[{E}_y\right]\left(s,t\right)}. $$

Here,

R8-3.4 $$ {R}_{X; Base}^{ID}\left(s,t\right)={\displaystyle {\sum}_{x=0}^{L(s)}{\displaystyle {\sum}_{l=1}^{L_I^{CO}}{r}_{I; Base}\left(x,l;s,t\right)}}+{\displaystyle {\sum}_{x_B=-{L}_D^{CO}+2}^{L(s)}{\displaystyle {\sum}_{x_E= \max \left\{{x}_B,1\right\}}^{x_B+{L}_D^{CO}-1}{r}_{D; Base}\left({x}_B,{x}_E;s,t\right)}} $$

is the baseline exit rate. And

R8-3.5 $$ \Delta {R}_X^{ID}\left[{\mathrm{E}}_y\right]\left(s,t\right)\equiv {\displaystyle {\sum}_{x={x}_{B;y}(s)}^{x_{E;y}(s)-1}{\displaystyle {\sum}_{l=1}^{L_I^{CO}}\Delta {r}_I\left[{\mathrm{E}}_y\right]\left(x,l;s,t\right)}}+{\displaystyle {\sum}_{x_B={x}_{B;y}(s)}^{x_{E;y}(s)}{\displaystyle {\sum}_{x_E={x}_B}^{x_{E;y}(s)}\Delta {r}_D\left[{\mathrm{E}}_y\right]\left({x}_B,{x}_E;s,t\right)}} $$

is the increment of the exit rate confined in, and dependent only on, Ε_*y*_(*s*) (*y* = 1, …, *Y*). As explained at the bottom of subsection R8-1, *R*_*X*;*Base*_^*ID*^(*s*, *t*) alone gives factorable alignment probabilities. And the increments, {Δ*R*_*X*_^*ID*^[Ε_*y*_](*s*, *t*)}_*y* = 1,…,*Y*_, behave independently of the portions of sequence states outside Ε_*y*_(*s*). Thus, if each indel event is *completely* confined in any of the Ε_*y*_(*s*)’s or in any spacer regions between neighboring Ε_*y*_(*s*)’s (Figure S3, panel a), the alignment probability can be expressed as the product of the overall factor and the contributions from Ε_*y*_(*s*)’s and within spacer regions. Even if some events within a Ε_*y*_(*s*) are separated from the others by at least a PAS, they must be put together into a single local indel history (panel a). A complexity arises because deletions may stick out of a Ε_*y*_(*s*), or even bridge two or more regions (panels b and c). The rates of such deletions and indels that are completely outside of the regions are given by the baseline rates. When a deletion sticks out of a region, the region will be extended to encompass the deletion, and all events within the extended region are lumped into a single local indel history (panel b). When a deletion bridges two or more regions, a “meta-region” encompassing all bridged regions is defined, and all events within the meta-region will form a single local indel history (panel c). In contrast, the indels completely outside of the regions should be independent of each other as long as they are separated by at least a PAS. Hence, under this model, the PWA probabilities are “factorable” in this somewhat non-trivial sense.

In Supplementary appendix SA-3 in Additional file 2, we explicitly showed that the probability of a LHS equivalence class via the recipe of [21] is identical to that calculated via our *ab initio* formulation. Although we assumed the space-homogeneity there, the proof can probably be extended to the model in this subsection as well, by slightly modifying the definition of the “chop-zone”.

### R9. Merits, possible extensions & applications, and outstanding issues

In this paper, we presented a theoretical formulation built up by tools that help mathematically precise dissection of the *ab initio* calculation of alignment probabilities under genuine stochastic evolutionary models. Another merit of this formulation is that it gives intuitively clear pictures. For example, the insertion and deletion operators simply mathematically represent the intuitive pictures of the indels naturally acting on sequences (Fig. 3). Thus, the action of the rate operator, given by Eqs. (R3.6-R3.10) (or Eqs. (R3.11-R3.15)), is intuitively understandable as merely the collection of all possible single-mutational channels from a given sequence state (and some compensating terms). Then, the expansion formula for the action of the finite-time transition operator, Eqs. (R4.6,R4.7), can also be intuitively interpreted as the collection of contributions from all possible mutational processes starting with an initial sequence state. Importantly, this expansion was not posed via a hand-waving argument but rigorously derived as the solution of the defining equations of the model (Eqs. (R3.19-R3.21)), which justifies its *ab initio* status. And the integral equations, Eqs. (R4.4,R4.5), bridged the expansion’s mathematically rigorous and intuitive aspects. Finally, the binary equivalence relations, Eqs. (R5.2a-R5.2d) (*e.g*., Fig. 5), and their resulting LHS equivalence classes also allow intuitive interpretations as the invariance of the local effects of indels under their relative orders with spatially separate events. Therefore, the conditions for the factorability of PWA probabilities are also intuitively understandable. Although their mathematically rigorous derivations (in Supplementary appendix SA-2 in Additional file 2) might appear somewhat formidable, they are actually not so difficult once the aforementioned intuitive pictures are understood. Hence, by coupling the mathematical preciseness with the intuitive clarity, our theoretical formulation is expected to facilitate further advances of the study of *ab initio* alignment probabilities under genuine stochastic evolutionary models with some biological realism.

For clarity, this study focused only on indels among various mutational types, because indels are essential for creating a sequence alignment. If desired, however, our theoretical formulation could also incorporate substitutions ([31]; see also [42]). Moreover, the formulation could also be extended to incorporate other genome rearrangements, such as duplications and inversions. (See [31] for an initial, rudimentary attempt.) Such an extended formulation will provide tools to enable concrete analyses of “rate grammars” extended to incorporate genome rearrangements (briefly mentioned in [21]).

The practical use of our formulation depends on how efficiently it can calculate quite accurate alignment probabilities. Although the factorability of alignment probabilities will help greatly speed up the computation, the contribution from each local region (*e.g*., Eq. (R6.8) or Eq. (SM-4.22) in Additional file 1) is still composed of infinitely many terms. Good news is that the first approximation of each local contribution, which is the summation of the terms from parsimonious local indel histories alone, is quite accurate, as long as the gap lengths and the branch lengths are at most moderate (Ezawa, unpublished; draft manuscripts: [40, 41]). Thus, considerably efficient computation of considerably accurate alignment probabilities may be possible based on our formulation. Especially, at least when the model is spatiotemporally homogeneous, our *ab initio* calculation was shown to be equivalent to the calculation of [21], with a caveat (see subsection R8-1). Thus, their dynamic programming (DP) may be applicable, possibly with some modifications, to a wider class of models with factorable probabilities.

Despite these favorable aspects, our theoretical formulation still has some limitations and outstanding issues. First, we did not examine whether our “sufficient and nearly necessary” set of conditions for the alignment probability factorization is exactly necessary and sufficient or not. Nor did we provide any counterexamples that violate our set of conditions and still have factorable alignment probabilities. Solving these problems may be interesting at least mathematically.

Second, although in this study we *tentatively* used the set of positive integers to represent the space of ancestry indices (*ϒ*), it is obviously not the best choice. Finding, or establishing, a mathematical entity (either a set or a space) that is more suitable for representing *ϒ* should be another mathematically interesting issue.

Third, in section R8 (and in section 5 of [32]), we only considered simple boundary conditions. Each sequence end was either freely mutable or flanked by a biologically essential region that allows no indels. These boundary conditions may remain good approximations if the subject sequences were extracted from well-characterized genomic regions. In real sequence analyses, however, the ends of the aligned sequences are often determined by artificial factors, such as the methods to sequence the genome, detect sequence homology, and annotate the sequences. Moreover, the constant cutoff lengths (*L*_*I*_^*CO*^ and *L*_*D*_^*CO*^) were introduced just for the sake of simplicity, to broadly take account of the effects of various factors that suppress very long indels (such as selection, chromosome size, genome stability, *etc*.). In reality, it is much more likely that the cutoff lengths will vary across regions. Then, the alignment probabilities would be only approximately factorable, as in subsection R8-2. In order to pursue further biological realism and to enable more accurate sequence analyses, it should be inevitable to address these issues seriously. Eq. (R8-2.4) may be useful for such studies.

Fourth, we strongly caution the readers that, at this point, a naïve application of our formulation or its algorithmic implementation [41] to a *reconstructed* MSA is fraught with high risks of incorrect predictions of indel histories, *etc*. This is because reconstructed MSAs, *even if they were reconstructed via state-of-the-art aligners* (reviewed, *e.g*., in [43]), are known to be considerably erroneous (*e.g*., [42, 44, 45]). Thus, it should be preferable to first develop a method or a program that accurately assesses and rectifies alignment errors, preferably by estimating the distribution of fairly likely alternative MSAs (as, *e.g*., in [16, 46, 47]), before using our formulation to make some evolutionary or biological predictions.

Fifth, in this study, the phylogenetic tree was regarded as given. In many cases, however, the phylogenetic trees must also be inferred from the input sequence data. A theoretically ideal way would be to infer the joint distribution of MSAs and phylogenetic trees, as it is expected to minimize possible prediction biases (*e.g*., [13, 48–50]). A major problem is that such an analysis would be tremendously time-consuming in general. At present, it is a totally open question whether our formulation can be adapted to infer a quite accurate joint distribution efficiently enough.

## Conclusions

To the best of our knowledge, this is the first study to theoretically dissect the *ab initio* calculation of alignment probabilities under a *genuine* stochastic evolutionary model, which describes the evolution of an *entire* sequence via insertions and deletions (indels) along the time axis. The model handled here extends the previously most general evolutionary model, *i.e.*, the general form of the “substitution/inserton/deletion models” [21]. It should be noted that we did not impose any unnatural restrictions such as the prohibition of overlapping indels. Nor did we make the pre-proof assumption that the probability is factorable into the product of column-to-column transition probabilities or block-wise contributions. The essential tool introduced in this study was the operator representation of indels. This enabled us to shift the focus from the trajectory of sequence states (as in [21]) to the series of indel events, and to define local-history-set (LHS) equivalence classes of indel histories. Moreover, the operator representation also facilitated the adaptation of the time-dependent perturbation expansion (*e.g.*, [29, 30, 33]), which enabled us to express the probability of an alignment as a summation of probabilities over all alignment-consistent indel histories. Then, under a most general set of indel rate parameters, we exploited the LHS equivalence classes and found a “sufficient and nearly necessary” set of conditions on the indel rate parameters and exit rates under which the *ab initio* alignment probabilities can be factorized to provide a sort of generalized HMMs. We also showed that quite a wide variety of indel models could satisfy this set of conditions. Such models include not only the “long indel” model [21] and the indel model of a genuine molecular evolution simulator, Dawg [26], but also some sorts of models with rate variation across regions. Moreover, we explicitly showed (in Supplementary Appendix SA-3 in Additional file 2) that, as far as each LHS equivalence class is concerned, the probability calculated via the method of [21] is equivalent to that calculated via our *ab initio* formulation, at least under their spatiotemporally homogeneous indel model.

To summarize, by depending purely on the first principle and by providing intuitively clear pictures, this study established firm theoretical grounds that will help further advance the *ab initio* calculation of alignment probabilities under genuine stochastic evolutionary models with some biological realism. And our theoretical formulation will also provide other indel probabilistic models with a sound reference point, *provided that* there exist approximate methods that can quite accurately estimate the *ab initio* alignment probabilities fairly efficiently. Such approximate methods will be the subject of a related study (Ezawa, unpublished; draft manuscripts available [40, 41]).

## Methods

Methodological details in this study are described in Supplementary methods in Additional file 1, or in Supplementary appendix in Additional file 2.

## Abbreviations

HMM, hidden Markov model; indel(s), insertion(s)/deletion(s); LHS, local history set; MSA, multiple sequence alignment; PAS, preserved ancestral site; PWA, pairwise (sequence) alignment; SID model, substitution/insertion/deletion model

## Additional files

Additional file 1: A PDF file that consists of Supplementary methods (sections SM-1 through SM-4), Figures S1, S2, S3, and Table S1. The sections of Supplementary methods provide detailed derivations of some essential results. Figures S1-S3 graphically illustrate via MSAs some important concepts and ideas introduced in the main text. Table S1 summarizes mathematical symbols commonly used in this paper. (PDF 11751 kb) Additional file 2: A PDF file that consists of Supplementary appendix (sections SA-1, SA-2 and SA-3). The sections of Supplementary appendix provide mathematical derivations of some important results. (PDF 9581 kb)

## Footnotes

1 In a sense, the models implemented in the genuine sequence evolution simulators (*e.g*., [26–28]) could also be considered as special cases of this evolutionary model.

2 This poses no problem here because the summation is always bounded from above (by a number less than or equal to unity) and all terms in the summation are non-negative, which guarantees the convergence of the expansion.

3 However, the two manuscripts differ in some aspects, *e.g*., their starting points. In [32], we described our model from scratch, as a continuous-time Markov model, and only briefly discussed its relationship with the general SID model [21]. In contrast, this manuscript explicitly presents our model as an extension of the general SID model, because that would put our model into the historical context of studies on indel evolutionary models.

4 It may be worth mentioning, though, that the operator notation allows flexible representations of general state changes (by mutations). In the vector-matrix notation, this is possible only via abstract mutation matrices (and state vectors); concrete matrices (and vectors) can at most describe some fixed specific indel histories.

5 In Eq.(R1.1), *ω*(∈*Ω*) and *ω*^′^(∈*Ω*), respectively, are the residues before and after the substitution. *s*_*I*_ in Eq.(R1.2) denotes the inserted subsequence, and *s*_*D*_ in Eq.(R1.3) denotes the deleted subsequence. And the equation, *s* = *s*_*L*_*ωs*_*R*_, for example, states that the sequence *s* is formed by concatenating the three factors, *s*_*L*_, *ω* and *s*_*R*_.

6 Besides, as claimed in [21], the rate grammar could be extended to formally describe a wide variety of evolutionary processes, including duplication, inversion and translocation.

7 This should be understood as a brief representation. The precise representation is: $\breve {s}={\left({\overrightarrow{\upsilon}}^t,{\overrightarrow{\omega}}^t\right)}^t$, where *A*^*t*^ denotes the transposition (*i.e.*, swapping the roles of rows and columns) of matrix *A*. (See Figure 2b.)

8 Inspired by profile HMMs (*e.g*., [51]) and the idea of position-specific evolutionary rates [25], we originally devised ancestry indices in order to distinguish columns of a given alignment from one another. Their values themselves are not considered so important. Thus, it would be convenient to *re-assign* the ancestry indices as follows, after an indel process (or history) created an alignment, *α*, whether it is among only extant sequences or among sequence states at all nodes of the phylogenetic tree. (1) Re-assign 1, 2, …, |*α*| (= the number of columns in *α*) to the sites corresponding to the columns of *α*, from left to right. (2) Re-assign |*α*| + 1, |*α*| + 2, …. to the “evanescent” sites with no corresponding alignment columns, again from left to right. This re-assignment can be considered as replacing a set of aligned sequence states created by an indel process (or history) with a “representative” set “equivalent” to the former set in the sense that both give the same homology structure [48]. The re-assignment may facilitate the understanding of the arguments in sections R5 through R8. (The figures in this paper do not undergo this re-assignment, though.)

9 As far as the sequence (with state $\breve {s}$) is concerned, ${\widehat{M}}_D\left({x}_B,{x}_E\right)$ with *x*_*B*_ < 1, is indistinguishable from ${\widehat{M}}_D\left(1,{x}_E\right)$, and ${\widehat{M}}_D\left({x}_B,{x}_E\right)$ with ${x}_E>L\left(\breve {s}\right)$ is indistinguishable from ${\widehat{M}}_D\left({x}_B,L\left(\breve {s}\right)\right)$.

10 In this paper, *α*(…) (or *α*[…]) is intended to denote the *homology structure* [48] of an alignment, and thus the symbol doesn’t care about details on the ancestry indices or residue states filling in the alignment other than the distinction of different columns in the alignment. And, hereafter, the terms “alignment”, “PWA”, and “MSA” mean their homology structures.

11 Actually, we could also define the equivalence relationships between products of non-separated indel operators (non-exhaustively listed in Appendix A1 of [32]), and they may assist further theoretical developments. However, discussing these extra equivalences is beyond the scope of this manuscript.

12 Such a maximum set does not necessarily consist of *all* PASs in the PWA. An example is given in subsection R8-3.

13 The “phylogenetic correctness” condition guarantees that the sites aligned in a MSA column should share an ancestry. The condition could be rephrased as: “if a site corresponding to the column is present at two points in the phylogenetic tree, the site must also be present all along the shortest path connecting the two points”.

14 HMMs commonly use geometric distributions of sequence lengths. The uniform distribution may be a good approximation if we can assume that the ancestral sequence was sampled randomly from a chromosome of length *L*_*C*_. In this case, the distribution of the sequence length, *L*(*s*)(< < *L*_*C*_), would be proportional to (1 − (*L*(*s*) − 1)/*L*_*C*_) ≈ 1.

15 ${\tilde{\overset{\smile }{\mathrm{M}}}}_P\left[\alpha \left[{s}_1,{s}_2,\dots, {s}_{N^X}\right];{s}_0^{Root};{C}_K\left|T\right.\right]$ should be equivalent to ${\tilde{\overset{\smile }{\mathrm{M}}}}_P\left[{\tilde{\Lambda}}_{\Psi}^{ID}\left[{C}_{\mathrm{K}};\alpha \left[{s}_1,{s}_2,\dots, {s}_{N^X}\right];T\right]\left|T\right.\right]$ given in Eq.(4.2.9c) of [32], although the two expressions may appear quite different at first glance.

16 Of course, we can also consider a model where only a single essential site/region flanks a rate-homogeneous neutral region. Alignment probabilities of this model can be shown to be factorable because the exit rate is an affine function of the sequence length (subsection 5.2 of [32]).
