# Erratum to: General continuous-time Markov model of sequence evolution via insertions/deletions: are alignment probabilities factorable?

**DOI:** 10.1186/s12859-016-1282-4

**Published:** 2016-11-10

**Authors:** Kiyoshi Ezawa

**Affiliations:** 1 Department of Bioscience and Bioinformatics, Kyushu Institute of Technology, Iizuka, 820-8502 Japan; 2 Department of Biology and Biochemistry, University of Houston, Houston, TX 77204-5001 USA

## Erratum

Unfortunately, [1] was published with errors in the equations and text. Please find further details below.

In the following sentences ‘*š* ^*II*^’ should be capitalised to be ‘*Š* ^II^’: ‘Because of the rules imposed above, the space of the extended sequence states (denoted as *š* ^*II*^ in [31]) is included in but never equal to ’ (the last sentence of the first paragraph of section R2) and ‘Because the state space we are working in, *š* ^*II*^, is essentially infinite, we cannot give the explicit matrix expression of the rate operator on the entire state space. Nevertheless, the rate operator can be defined if we give its action on every state in *š* ^*II*^ ’ (the two sentences above Eq.(R3.1)).

Therefore the correct sentences are: ‘Because of the rules imposed above, the space of the extended sequence states (denoted as *Š* ^*II*^ in [31]) is included in but never equal to ’ and ‘Because the state space we are working in, *Š* ^*II*^, is essentially infinite, we cannot give the explicit matrix expression of the rate operator on the entire state space. Nevertheless, the rate operator can be defined if we give its action on every state in *Š* ^*II*^.’

In Equation (R4.6) below, there should only be one comma after ‘$\left[{\widehat{M}}_1,{\widehat{M}}_2,,,\dots, {\widehat{M}}_N\right]$ *’*.

$$ {\left\langle s\right.}_0\left|{\widehat{P}}^{I D}\right.\left({t}_I,{t}_F\right)=\begin{array}{cc}\hfill \sum_{N=0}^{\infty}\hfill & \hfill {\displaystyle \sum_{{}_{\left[{\widehat{M}}_1,{\widehat{M}}_2,,,\dots, {\widehat{M}}_N\right]\in {H}^{I D}\left( N;{8}_0\right)}}}\hfill \end{array} P\left[\left(\left[{\widehat{M}}_1,{\widehat{M}}_2,,,\dots, {\widehat{M}}_N\right],,,\left[{t}_I,{t}_F\right]\right)\left|\left({s}_0,{t}_I\right)\right.\right]\left\langle {s}_0\left|{\widehat{M}}_1{\widehat{M}}_2\dots {\widehat{M}}_N.\right.\right. $$

Besides, the triple commas in the expression, ‘$\left[{\widehat{M}}_1,{\widehat{M}}_2,,,\dots, {\widehat{M}}_N\right]$ *’*, should also be a single comma.

Therefore the correct equation is as below:

$$ {\left\langle s\right.}_0\left|{\widehat{P}}^{I D}\right.\left({t}_I,{t}_F\right)=\begin{array}{cc}\hfill \sum_{N=0}^{\infty}\hfill & \hfill {\displaystyle \sum_{{}_{\left[{\widehat{M}}_1,{\widehat{M}}_2,\dots, {\widehat{M}}_N\right]\in {H}^{I D}\left( N;{8}_0\right)}}}\hfill \end{array} P\left[\left(\left[{\widehat{M}}_1,{\widehat{M}}_2,\dots, {\widehat{M}}_N\right],\left[{t}_I,{t}_F\right]\right)\left|\left({s}_0,{t}_I\right)\right.\right]\left\langle {s}_0\left|{\widehat{M}}_1{\widehat{M}}_2\dots {\widehat{M}}_N.\right.\right. $$

In Equation (R5.4) below, the ‘K’ in front of the ‘N_1_’ should be a ‘1’

$$ \left\langle {s}^D\right.\left|=\right.\left\langle {s}^A\right.\left|\left[\widehat{M}\left[ K,1\right]\dots \widehat{M}\left[ K,{N}_K\right]\right]\dots \left[\widehat{M}\left[1,1\right]\dots \widehat{M}\left[ K,{N}_1\right]\right]\right.. $$

Therefore the correct equation is as below:

$$ \left\langle {s}^D\right.\left|=\right.\left\langle {s}^A\right.\left|\left[\widehat{M}\left[ K,1\right]\dots \widehat{M}\left[ K,{N}_K\right]\right]\right.\dots \left[\widehat{M}\left[1,1\right]\dots \widehat{M}\left[1,{N}_1\right]\right]. $$

In Equation (R6.4) below, two of the square brackets should be round parentheses:

$$ {\mu}_P\left[\left({\left[\overset{\rightharpoonup }{\overset{\rightharpoonup }{\widehat{M}}}\right]}_{LHS},\left[{t}_I,{t}_F\right]\right)\Big|\left({s}^A,{t}_I\right)\right]\equiv P\left[\left({\left[\overset{\rightharpoonup }{\overset{\rightharpoonup }{\widehat{M}}}\right]}_{LHS},\left[{t}_I,{t}_F\right]\right)\Big|\left({s}^A,{t}_I\right)\right]/ P\left[\left[\left[\right],\left[{t}_I,{t}_F\right]\right]\Big|\left({s}^A,{t}_I\right)\right], $$

Therefore the correct equation is as below:

$$ {\mu}_P\left[\left({\left[\overset{\rightharpoonup }{\overset{\rightharpoonup }{\widehat{M}}}\right]}_{LHS},\left[{t}_I,{t}_F\right]\right)\Big|\left({s}^A,{t}_I\right)\right]\equiv P\left[\left({\left[\overset{\rightharpoonup }{\overset{\rightharpoonup }{\widehat{M}}}\right]}_{LHS},\left[{t}_I,{t}_F\right]\right)\Big|\left({s}^A,{t}_I\right)\right]/ P\left[\left(\left[\right],\left[{t}_I,{t}_F\right]\right)\Big|\left({s}^A,{t}_I\right)\right], $$

In Equation (R7.7) below, the part of the equation starting with theta (i.e.,‘${\varTheta}_{\mathrm{ID}}$(b)’) should have been subscript text:

$$ \begin{array}{l} P\left[\left(\alpha \left({s}^A(b),{s}^D(b)\right), b\right)\left|\left({s}^A(b),{n}^A(b)\right)\right.\right]\\ {}\equiv P\left[\left(\alpha \left({s}^A(b),{s}^D(b)\right),\left[ t\left({n}^A(b)\right), t\left({n}^D(b)\right)\right]\right)\right.\left|\left({s}^A(b), t\left({n}^A(b)\right)\right)\right]\left|{\varTheta}_{ID}(b)\right)\end{array} $$

Therefore the correct equation is as below:

$$ \begin{array}{l} P\left[\left(\alpha \left({s}^A(b),{s}^D(b)\right), b\right)\left|\left({s}^A(b),{n}^A(b)\right)\right.\right]\\ {}\equiv P\left[\left(\alpha \left({s}^A(b),{s}^D(b)\right),\left[ t\left({n}^A(b)\right), t\left({n}^D(b)\right)\right]\right)\right.\left|\left({s}^A(b), t\left({n}^A(b)\right)\right)\right]\Big|{}_{\varTheta_{ID}(b)}\end{array} $$

In Equation (R8-2.4) below, the triple commas should be single commas:

$$ \delta \delta {R}_X^{ID}\left( s^{\prime \prime\prime }, s^{\prime \prime }, s^{\prime },,, s, t\right)\equiv \delta {R}_X^{ID}\left( s^{\prime \prime\prime }, s^{\prime \prime },,, t\right)-\delta {R}_X^{ID}\left( s^{\prime }, s, t\right), $$

Therefore correct equation is as below

$$ \delta \delta {R}_X^{ID}\left( s^{\prime \prime\prime }, s^{\prime \prime }, s^{\prime }, s, t\right)\equiv \delta {R}_X^{ID}\left( s^{\prime \prime\prime }, s^{\prime \prime }, t\right)-\delta {R}_X^{ID}\left( s^{\prime }, s, t\right), $$

Finally, the manuscript referred to as "Ezawa, unpublished" in [1] has already been published [2].

The author is grateful to Aradhana Mistry, Senior Production Editor of BioMed Central, for kindly drafting this erratum. The author also appreciates the assistance of all staff members of BioMed Central who were involved in the publication of the erratum and/or the original article [1]
