# Supplementary material for: General continuous-time Markov model of sequence evolution via insertions/deletions: are alignment probabilities factorable?
Source: BMC Bioinformatics. 2016 Aug 11;17:304. doi: 10.1186/s12859-016-1105-7 (PMC5026781; doi:10.1186/s12859-016-1105-7)
Supplement: Additional file 1: — A PDF file that consists of Supplementary methods (sections SM-1 through SM-4), Figures S1, S2, S3, and Table S1. The sections of Supplementary methods provide detailed derivations of some essential results. Figures S1-S3 graphically illustrate via MSAs some important concepts and ideas introduced in the main text. Table S1 summarizes mathematical symbols commonly used in this paper. (PDF 11751 kb) [file 12859_2016_1105_MOESM1_ESM.pdf]

Additional file 1 of
"General continuous-time Markov model of sequence evolution via insertions/deletions: Are alignment probabilities factorable?"

by Kiyoshi Ezawa

Table of contents

Supplementary methods **pp. 2-22**

SM-1. Perturbation expansion of finite-time transition operator and pairwise alignment probability: details pp. 2-6

SM-2. Factorability of pairwise alignment probability: details pp. 6-12

SM-3. Factorability of probability of simplest LHS equivalence class pp. 12-13

SM-4. Factorability of multiple sequence alignment probability: details pp. 14-22

Supplementary figures (with legends) **pp. 23-28**

Supplementary table (S1) **pp. 29-36**

Supplementary methods

SM-1. Perturbation expansion of finite-time transition operator and pairwise alignment probability: details

Here, we apply the technique of time-dependent perturbation expansion (*e.g.*, [29,30]) to our evolutionary model. We first re-express our rate operator as:

$$\hat{Q}^{ID}(t) = \hat{Q}_0^{ID}(t) + \hat{Q}_M^{ID}(t)\,. \quad \text{--- Eq.(SM-1.1)}$$

(It corresponds to Eq.(R4.1).) Here $\hat{Q}_0^{ID}(t) \equiv \hat{Q}_X^{I}(t) + \hat{Q}_X^{D}(t)$ describes the mutation-free evolution, and $\hat{Q}_M^{ID}(t) \equiv \hat{Q}_M^{I}(t) + \hat{Q}_M^{D}(t)$ describes the single-mutation transition between states. From the reduced form of Eq.(R3.6), we get:

$$\langle s|\hat{Q}_0^{ID}(t) \;=\; -R_X^{ID}(s,t)\langle s|\,, \quad \text{--- Eq.(SM-1.2)}$$

with $R_X^{ID}(s,t) \equiv R_X^{I}(s,t) + R_X^{D}(s,t)$. --- Eq.(SM-1.3)

(Eq.(SM-1.2) and Eq.(SM-1.3) correspond to Eq.(R4.2) and Eq.(R4.3), respectively.) Using the decomposition, Eq.(SM-1.1), the forward equation, Eq.(R3.19), can be rewritten as:

$$\frac{\partial}{\partial t'}\hat{P}^{ID}(t,t') - \hat{P}^{ID}(t,t')\,\hat{Q}_0^{ID}(t') = \hat{P}^{ID}(t,t')\,\hat{Q}_M^{ID}(t')\,. \quad \text{--- Eq.(SM-1.4)}$$

Now, let $\hat{P}_0^{ID}(t',t'') \equiv T\left\{\exp\left(\int_{t'}^{t''} d\tau\, \hat{Q}_0^{ID}(\tau)\right)\right\}$, and multiply it from the right of each side of Eq.(SM-1.4). Then, exploiting the equation, $\frac{\partial}{\partial t'}\hat{P}_0^{ID}(t',t'') = -\hat{Q}_0^{ID}(t')\,\hat{P}_0^{ID}(t',t'')$, we get:

$$\frac{\partial}{\partial t'}\left\{\hat{P}^{ID}(t,t')\,\hat{P}_0^{ID}(t',t'')\right\} \;=\; \hat{P}^{ID}(t,t')\,\hat{Q}_M^{ID}(t')\,\hat{P}_0^{ID}(t',t'')\,. \quad \text{--- Eq.(SM-1.5)}$$

Integrating the both sides over time $t' \in [t, t'']$, using $\hat{P}^{ID}(t,t) = \hat{P}_0^{ID}(t'',t'') = \hat{I}$, and replacing t'' with t', we finally obtain a crucial integral equation:

$$\hat{P}^{ID}(t,t') \;=\; \hat{P}_0^{ID}(t,t') + \int_t^{t'} d\tau\, \hat{P}^{ID}(t,\tau)\,\hat{Q}_M^{ID}(\tau)\,\hat{P}_0^{ID}(\tau,t')\,. \quad \text{--- Eq.(SM-1.6)}$$

(It corresponds to Eq.(R4.4).) Similarly, starting from the backward equation, Eq.(R3.20), we can obtain another crucial integral equation:

$$\hat{P}^{ID}(t,t') \;=\; \hat{P}_0^{ID}(t,t') + \int_t^{t'} d\tau\, \hat{P}_0^{ID}(t,\tau)\,\hat{Q}_M^{ID}(\tau)\,\hat{P}^{ID}(\tau,t')\,. \quad \text{--- Eq.(SM-1.7)}$$

(It corresponds to Eq.(R4.5).) These equations are equivalent to the defining differential equations, Eqs.(R3.19-21), because the former were directly derived from the latter. (And the latter can also be derived from the former.)

Now, to formally solve Eq.(SM-1.6), we assume that the solution can be expanded as: $\hat{P}^{ID}(t,t') = \sum_{N=0}^{\infty} \hat{P}^{ID}_{(N)}(t,t')$, where $\hat{P}^{ID}_{(N)}(t,t')$ is the collection of terms containing N indel operators each. Substituting this expansion into Eq.(SM-1.6) and comparing the terms with the same number of indel operators, we find the equations:

$$\hat{P}^{ID}_{(0)}(t,t') = \hat{P}^{ID}_{0}(t,t'), \quad \hat{P}^{ID}_{(N+1)}(t,t') = \int_t^{t'} d\tau \, \hat{P}^{ID}_{(N)}(t,\tau)\, \hat{Q}^{ID}_{M}(\tau)\, \hat{P}^{ID}_{0}(\tau,t') \text{. --- Eqs.(SM-1.8,9)}$$

Using Eq.(SM-1.8) as an initial condition, Eq.(SM-1.9) can be recursively solved to give:

$$\hat{P}^{ID}_{(N)}(t,t') = \int\cdots\int_{t<\tau_1<\cdots<\tau_N<\tau_{N+1}=t'} d\tau_1 \cdots d\tau_N \; \hat{P}^{ID}_{0}(t,\tau_1)\, T\left\{ \prod_{\nu=1}^{N} \hat{Q}^{ID}_{M}(\tau_\nu)\, \hat{P}^{ID}_{0}(\tau_\nu,\tau_{\nu+1}) \right\} \text{ --- Eq.(SM-1.10)}$$

for $N \geq 1$. Substituting this back into the above expansion, we finally get the formal perturbation expansion of the finite-time transition operator:

$$\hat{P}^{ID}(t,t') = \hat{P}^{ID}_{0}(t,t') + \sum_{N=1}^{\infty} \int\cdots\int_{t<\tau_1<\cdots<\tau_N<\tau_{N+1}=t'} d\tau_1 \cdots d\tau_N \; \hat{P}^{ID}_{0}(t,\tau_1)\, T\left\{ \prod_{\nu=1}^{N} \hat{Q}^{ID}_{M}(\tau_\nu) \hat{P}^{ID}_{0}(\tau_\nu,\tau_{\nu+1}) \right\}$$

$$= \hat{P}^{ID}_{0}(t_I,t_F) + \int_t^{t'} d\tau \; \hat{P}^{ID}_{0}(t,\tau)\, \hat{Q}^{ID}_{M}(\tau) \hat{P}^{ID}_{0}(\tau,t')$$

$$+ \iint_{t<\tau_1<\tau_2<t'} d\tau_1 d\tau_2 \; \hat{P}^{ID}_{0}(t,\tau_1)\, \hat{Q}^{ID}_{M}(\tau_1) \hat{P}^{ID}_{0}(\tau_1,\tau_2) \hat{Q}^{ID}_{M}(\tau_2) \hat{P}^{ID}_{0}(\tau_2,t')$$

$$+ \iiint_{t<\tau_1<\tau_2<\tau_3<t'} d\tau_1 d\tau_2 d\tau_3 \; \hat{P}^{ID}_{0}(t,\tau_1)\, \hat{Q}^{ID}_{M}(\tau_1) \hat{P}^{ID}_{0}(\tau_1,\tau_2) \hat{Q}^{ID}_{M}(\tau_2) \hat{P}^{ID}_{0}(\tau_2,\tau_3) \hat{Q}^{ID}_{M}(\tau_3) \hat{P}^{ID}_{0}(\tau_3,t') + \cdots .$$

--- Eq.(SM-1.11)

Note that Eq.(SM-1.11) can be derived also from Eq.(SM-1.7). Because of Eq.(SM-1.2), the equation:

$$\langle s | \hat{P}^{ID}_{0}(t,t') = \exp\left(-\int_t^{t'} d\tau \; R^{ID}_{X}(s,\tau) \right) \langle s | \quad \text{--- Eq.(SM-1.12)}$$

always holds for every state $s \in S^{II}$ and any time points $(t,t') \in [t_I,t_F]^2$ (with $t < t'$). Thus, $\hat{P}^{ID}_{0}(t,t')$ describes the state retention during the time interval, $[t,t']$, with the retention probability exponentially decreasing at the exit rate ($R^{ID}_{X}(s,\tau)$). Therefore, the N-th term in the solution, Eq.(SM-1.11), literally describes the evolutionary processes where the sequence underwent exactly N mutations. In his theorems 1 and 2, Feller [35] mathematically proved that the conditional probability, Eq.(R3.17), obtained by substituting Eq.(SM-1.11) for $\hat{P}^{ID}(t,t')$ is the solution of the defining time-differential equations of a continuous-time Markov model (the probability versions of Eqs.(R3.19-21)). In his paper presenting a widely used method for stochastic simulations, Gillespie [34] in effect gave a more intuitive derivation of the solution. Gillespie's method is crucial for molecular evolutionists, because it gives the basis of the *genuine* molecular evolution simulators (*e.g.*, [26,27,28]). Our

derivation of the solution, Eq.(SM-1.11), serves as a bridge between Feller's mathematically rigorous proof and Gillespie's intuitive derivation. Ours also helps understand the situation underlying Feller's theorems and gives an intuitively clearer view via the neat operator representation of the solution. [NOTE: Besides, our derivation via perturbation expansion is more flexible than theirs, because our method can go beyond the separation of exit rate terms from transition terms (see, *e.g.*, [31]).]

Now, examine the action of Eq.(SM-1.11) (with (t, t') replaced by (t_I, t_F)) on every basic state $s_0 \in S^{II}$. To simplify the argument, we symbolically rewrite the action of $\hat{Q}_M^{ID}(t) \equiv \hat{Q}_M^{I}(t) + \hat{Q}_M^{D}(t)$ on a bra-vector $\langle s|$ as:

$$\langle s|\hat{Q}_M^{ID}(t) \;=\; \sum_{\hat{M}\in \mathrm{M}^{ID}[L(s)]} r(\hat{M}; s,t)\langle s|\hat{M} \,. \quad \text{--- Eq.(SM-1.13)}$$

Here, $\mathrm{M}^{ID}[L] \equiv \left\{\hat{M}_I(x,l)\right\}_{0\le x\le L,\, 1\le l} \cup \left\{\hat{M}_D(x_B,x_E)\right\}_{x_B\le x_E,\, x_B\le L,\, 1\le x_E}$ denotes the set of insertion and deletion operators that can act on the sequence of length L, and $r(\hat{M}; s,t)$ denotes the (generally time- and basic-state-dependent) rate parameter of the indel operator $\hat{M}$. Now, operating each term of Eq.(SM-1.13) on $\langle s_0|$, replacing (t,t') by (t_I, t_F), and applying Eq.(SM-1.12) and Eq.(SM-1.13) alternately, we finally get:

$$\langle s_0|\hat{P}^{ID}(t_I,t_F) = \exp\left\{-\int_{t_I}^{t_F} d\tau\, R_X^{ID}(s_0,\tau)\right\}\langle s_0|$$
$$+\sum_{N=1}^{\infty}\ \sum_{[\hat{M}_1,\hat{M}_2,\cdots,\hat{M}_N]\in \mathrm{H}^{ID}(N;s_0)} P\Big[\big([\hat{M}_1,\hat{M}_2,...,\hat{M}_N],[t_I,t_F]\big)\,|\,(s_0,t_I)\Big]\langle s_0|\hat{M}_1\hat{M}_2\cdots\hat{M}_N \quad .$$

--- Eq.(SM-1.14)

Here, $\mathrm{H}^{ID}(N;s_0)$ denotes the space of all possible histories of N indels each that begin with the sequence state s_0. And

$$P\Big[\big([\hat{M}_1,\hat{M}_2,\cdots,\hat{M}_N],[t_I,t_F]\big)\,|\,(s_0,t_I)\Big]$$
$$=\int\!\cdots\!\int_{t_I=\tau_0<\tau_1<\cdots<\tau_N<\tau_{N+1}=t_F} d\tau_1\cdots d\tau_N\left(\prod_{\nu=1}^{N} r(\hat{M}_\nu; s_{\nu-1},\tau_\nu)\right)\exp\left\{-\sum_{\nu=0}^{N}\int_{\tau_\nu}^{\tau_{\nu+1}} d\tau\, R_X^{ID}(s_\nu,\tau)\right\}\Bigg|_{\left\{\langle s_\nu| = \langle s_{\nu-1}|\hat{M}_\nu \,|\, \nu=1,...,N\right\}}$$

--- Eq.(SM-1.15)

(, which corresponds to Eq.(R4.7),) is the probability that an indel history $[\hat{M}_1,\hat{M}_2,\cdots,\hat{M}_N]$ occurred during the time interval $[t_I,t_F]$, given an initial sequence state s_0 at time t_I. Eqs.(SM-1.14) supplemented by Eq.(SM-1.15) gives a considerably concrete expression of the solution of the defining equations, Eqs.(R3.19-21), of our genuine stochastic evolutionary model. (See subsection 3.1 of [32] for a more detailed explanation of Eqs.(SM-1.14,15).)

Now, let $\mathrm{H}^{ID}(N=0; s_0) \equiv \{(s_0, [\,])\}$ be the set consisting only of the history with zero indel, $[\,]$, starting with the state s_0. We can interpret $\exp\left\{-\int_{t_I}^{t_F} d\tau\, R_X^{ID}(s_0, \tau)\right\}$ as the conditional probability of this zero-indel history, $P\left[([\,],[t_I,t_F]) \mid (s_0, t_I)\right]$. Thus, Eq.(SM-1.14) can be rewritten more neatly as:

$$\langle s_0 | \hat{P}^{ID}(t_I, t_F) = \sum_{N=0}^{\infty} \sum_{[\hat{M}_1, \hat{M}_2, \cdots, \hat{M}_N] \in \mathrm{H}^{ID}(N; s_0)} P\left[([\hat{M}_1, \hat{M}_2, ..., \hat{M}_N],[t_I,t_F]) \mid (s_0, t_I)\right] \langle s_0 | \hat{M}_1 \hat{M}_2 \cdots \hat{M}_N .$$

--- Eq.(SM-1.14')

(It corresponds to Eq.(R4.6).)

Now, substitute an "ancestral" sequence state, $s^A \left(\in S^{II}\right)$, for s_0 in Eq.(SM-1.14'), and take the inner product between it and the ket-vector, $|s^D\rangle$, of a "descendant" sequence state, $s^D \left(\in S^{II}\right)$. This procedure gives the finite-time transition probability, $\langle s^A | \hat{P}^{ID}(t_I, t_F) | s^D \rangle = P\left[(s^D, t_F) \mid (s^A, t_I)\right]$, as the summation of probabilities over all possible indel histories consistent with the ancestral and descendant sequence states. As exemplified by Eq.(R2.1), the comparison of s^D with s^A uniquely determines the pairwise sequence alignment (PWA) between them, with a definite homology structure [48]. Let $\alpha(s^A, s^D)$ denote (the homology structure of) such a PWA. Then, summing the above transition probability, $\langle s^A | \hat{P}^{ID}(t_I, t_F) | s^D \rangle$, over all "equivalent" s^D's providing $\alpha(s^A, s^D)$ gives $P\left[(\alpha(s^A, s^D), [t_I, t_F]) \mid (s^A, t_I)\right]$, which is the probability that $\alpha(s^A, s^D)$ resulted from the evolution during the interval $[t_I, t_F]$, given s^A at t_I. By analogy to the derivation of Eq.(SM-1.14'), we obtain the formal expression of this probability as:

$$P\left[(\alpha(s^A, s^D), [t_I, t_F]) \mid (s^A, t_I)\right] = \sum_{N = N_{\min}\left[\alpha(s^A, s^D)\right]}^{\infty} \sum_{\substack{[\hat{M}_1, \hat{M}_2, \cdots, \hat{M}_N] \\ \in \mathrm{H}^{ID}\left[N; \alpha(s^A, s^D)\right]}} P\left[([\hat{M}_1, \hat{M}_2, \cdots, \hat{M}_N],[t_I,t_F]) \mid (s^A, t_I)\right] .$$

--- Eq.(SM-1.16)

(It corresponds to Eq.(R4.8).) Here, $\mathrm{H}^{ID}\left[N; \alpha(s^A, s^D)\right]$ denotes the set of all histories with

N indels each that can result in $\alpha(s^A, s^D)$, and $N_{\min}\left[\alpha(s^A, s^D)\right]$ is the minimum number of indels required for creating the PWA. Now, introduce the symbol that represents the set of all global indel histories consistent with $\alpha(s^A, s^D)$:

$$\tilde{\mathrm{H}}^{ID}\left[\alpha(s^A, s^D)\right] \equiv \bigcup_{N=N_{\min}[\alpha(s^A,s^D)]}^{\infty} \mathrm{H}^{ID}\left[N; \alpha(s^A, s^D)\right]. \quad \text{--- Eq.(SM-1.17)}$$

Then, Eq.(SM-1.16) can be further simplified as:

$$P\left[\left(\alpha(s^A,s^D),[t_I,t_F]\right) \middle| (s^A,t_I)\right] = \sum_{\substack{[\hat{M}_1,\hat{M}_2,\cdots,\hat{M}_N] \\ \in \tilde{\mathrm{H}}^{ID}\left[\alpha(s^A,s^D)\right]}} P\left[\left([\hat{M}_1,\hat{M}_2,\cdots,\hat{M}_N],[t_I,t_F]\right) \middle| (s^A,t_I)\right].$$

--- Eq.(SM-1.16')

(It corresponds to Eq.(R4.9).) Eq.(SM-1.16) and Eq.(SM-1.16') are the formal expressions of the occurrence probability of PWA $\alpha(s^A, s^D)$ derived purely from the defining equations, Eqs.(R3.19-21), of our evolutionary model. Thus, they are the "*ab initio* probability" of the PWA. In section SM-2, we will examine its factorability.

SM-2. Factorability of pairwise alignment probability: details

Here we examine the factorability of the *ab initio* probability of PWA $\alpha(s^A, s^D)$, $P\left[\left(\alpha(s^A,s^D),[t_I,t_F]\right) \middle| (s^A,t_I)\right]$ in Eq. (R4.9), given the ancestral state (s^A) at the initial time (t_I).

As mentioned in section R6 of Results and discussion, each component probability, $P\left[\left([\hat{M}_1,\hat{M}_2,\cdots,\hat{M}_N],[t_I,t_F]\right) \middle| (s^A,t_I)\right]$ given by Eq.(R4.7), will not be factorable. This is because its domain of multiple-time integration is not a direct product. Thus, we will need to combine the probabilities of a number of indel histories. How can we do this? As mentioned in Section R5, each indel history, $[\hat{M}_1, \hat{M}_2, \cdots, \hat{M}_N]$, belongs to a LHS equivalence class represented, *e.g.*, by a LHS, $\left\{\left[\hat{M}[k,1], ..., \hat{M}[k,N_k]\right]\right\}_{k=1,...,K}$, which will be abbreviated as $\vec{\vec{\hat{M}}}$ hereafter. Let $\left[\vec{\vec{\hat{M}}}\right]_{LHS}$ denote this LHS equivalence class. If $[\hat{M}_1, \hat{M}_2, \cdots, \hat{M}_N]$ can yield $\alpha(s^A, s^D)$, so can every element of the LHS that $[\hat{M}_1, \hat{M}_2, \cdots, \hat{M}_N]$ belongs to. Thus, obviously, we have $\left[\vec{\vec{\hat{M}}}\right]_{LHS} \subset \tilde{\mathrm{H}}^{ID}\left[\alpha(s^A, s^D)\right]$ for every $\left[\vec{\vec{\hat{M}}}\right]_{LHS}$ containing an indel history that can yield $\alpha(s^A, s^D)$. Next, if the two indel histories connect with each other through a

series of binary equivalence relations, Eqs.(R5.2a-d), the two histories belong to the same LHS equivalence class. These facts mean that the set $\tilde{\mathrm{H}}^{ID}\left[\alpha(s^A, s^D)\right]$ of all histories consistent with $\alpha(s^A, s^D)$ can be decomposed into a direct sum:

$$\tilde{\mathrm{H}}^{ID}\left[\alpha(s^A, s^D)\right] = \bigcup_{\vec{\vec{\hat{M}}}\in\tilde{\Lambda}^{ID}\left[\alpha(s^A, s^D)\right]} \left[\vec{\vec{\hat{M}}}\right]_{LHS} \quad . \text{--- Eq.(SM-2.1)}$$

(It corresponds to Eq.(R6.5).) Here, $\tilde{\Lambda}^{ID}\left[\alpha(s^A, s^D)\right]$ is the set of all LHSs consistent with $\alpha(s^A, s^D)$. This enables us to further rewrite the PWA probability, Eq.(R4.9), as:

$$P\left[\left(\alpha(s^A,s^D),[t_I,t_F]\right)\middle|\,(s^A,t_I)\right] = \sum_{\vec{\vec{\hat{M}}}\in\tilde{\Lambda}^{ID}\left[\alpha(s^A, s^D)\right]} P\left[\left(\left[\vec{\vec{\hat{M}}}\right]_{LHS},[t_I,t_F]\right)\middle|\,(s^A,t_I)\right] \quad .$$

--- Eq.(SM-2.2)

(It corresponds to Eq.(R6.6).) Here,

$$P\left[\left(\left[\vec{\vec{\hat{M}}}\right]_{LHS},[t_I,t_F]\right)\middle|\,(s^A,t_I)\right] \equiv \sum_{[\hat{M}_1,\hat{M}_2,\cdots,\hat{M}_N]\in\left[\vec{\vec{\hat{M}}}\right]_{LHS}} P\left[\left([\hat{M}_1,\hat{M}_2,\cdots,\hat{M}_N],[t_I,t_F]\right)\middle|\,(s^A,t_I)\right]$$

--- Eq.(SM-2.3)

(, which corresponds to Eq.(R6.1),) is the "total probability" of $\left[\vec{\vec{\hat{M}}}\right]_{LHS}$. Therefore, if Eq.(SM-2.3) can be factorized for every LHS $\vec{\vec{\hat{M}}}\in\tilde{\Lambda}^{ID}\left[\alpha(s^A, s^D)\right]$, the PWA probability, Eq.(SM-2.2), may also become factorable.

To examine the factorability of Eq.(SM-2.3), it is convenient to consider the quotients:

$$\begin{aligned}&\mu_P\left[\left([\hat{M}_1,\hat{M}_2,\cdots,\hat{M}_N],[t_I,t_F]\right)\middle|\,(s^A,t_I)\right]\\ \equiv\ & P\left[\left([\hat{M}_1,\hat{M}_2,\cdots,\hat{M}_N],[t_I,t_F]\right)\middle|\,(s^A,t_I)\right]\Big/P\left[\left([\,],[t_I,t_F]\right)\middle|\,(s^A,t_I)\right]\end{aligned} \quad , \text{--- Eq.(SM-2.4)}$$

$$\begin{aligned}&\mu_P\left[\left(\left[\hat{M}[k,1],...,\hat{M}[k,N_k]\right],[t_I,t_F]\right)\middle|\,(s^A,t_I)\right]\\ \equiv\ & P\left[\left(\left[\hat{M}[k,1],...,\hat{M}[k,N_k]\right],[t_I,t_F]\right)\middle|\,(s^A,t_I)\right]\Big/ P\left[\left([\,],[t_I,t_F]\right)\middle|\,(s^A,t_I)\right]\end{aligned} \quad , \text{--- Eq.(SM-2.5)}$$

and

$$\mu_P\left[\left(\left[\vec{\vec{\hat{M}}}\right]_{LHS},[t_I,t_F]\right)\middle|\,(s^A,t_I)\right] \equiv P\left[\left(\left[\vec{\vec{\hat{M}}}\right]_{LHS},[t_I,t_F]\right)\middle|\,(s^A,t_I)\right]\Big/P\left[\left([\,],[t_I,t_F]\right)\middle|\,(s^A,t_I)\right] \ ,$$

--- Eq.(SM-2.6)

and focus on the relationships between Eqs.(SM-2.4-6). (Eq.(SM-2.5) and Eq.(SM-2.6) correspond to Eq.(R6.3) and Eq.(R6.4), respectively.) This is because Eq.(SM-2.4), for example, can be expressed as:

$$\mu_P\Big[\big([\hat{M}_1, \hat{M}_2, \cdots, \hat{M}_N], [t_I, t_F]\big) \,\big|\, (s^A, t_I)\Big]$$

$$= \int\cdots\int_{t_I=\tau_0<\tau_1<\cdots<\tau_N<\tau_{N+1}=t_F} d\tau_1 \cdots d\tau_N \left(\prod_{\nu=1}^{N} r(\hat{M}_\nu; s_{\nu-1}, \tau_\nu)\right) \exp\left\{-\sum_{\nu=0}^{N}\int_{\tau_\nu}^{\tau_{\nu+1}} d\tau\, \delta R_X^{ID}(s_\nu, s^A, \tau)\right\}\Bigg|_{\left\{\substack{s_0=s^A,\\ \langle s_\nu|=\langle s_{\nu-1}|\hat{M}_\nu \,|\, \nu=1,\ldots,N}\right\}}$$

, --- Eq.(SM-2.7)

where $\delta R_X^{ID}(s, s', \tau) \equiv R_X^{ID}(s, \tau) - R_X^{ID}(s', \tau)$ is an increment of the exit rate. A similar expression applies also to Eq.(SM-2.5). Compared with Eq.(R4.7) (or Eq.(SM-1.15)), the merit of Eq.(SM-2.7) is that it enables us to focus on the regions of the sequence where the indels took place, if the evolutionary model has desirable properties (revealed below). Thus, for a LHS, $\bar{\bar{\hat{M}}} = \left\{\left[\hat{M}[k,1], \ldots, \hat{M}[k, N_k]\right]\right\}_{k=1,\ldots,K}$, we will set the following ansatz:

$$\mu_P\left[\left(\left[\bar{\bar{\hat{M}}}\right]_{LHS}, [t_I, t_F]\right) \,\Big|\, (s^A, t_I)\right] = \prod_{k=1}^{K} \mu_P\Big[\big(\big[\hat{M}[k,1], \ldots, \hat{M}[k, N_k]\big], [t_I, t_F]\big) \,\big|\, (s^A, t_I)\Big],$$

--- Eq.(SM-2.8)

(, which corresponds to Eq.(R6.2),) and seek to find a set of conditions under which it indeed holds. To get a hint on the conditions, we will look at the both sides of Eq.(SM-2.8) more closely. Using Eq.(SM-2.3) and Eq.(SM-2.7), the left hand side of Eq.(SM-2.8) can be rewritten as:

$$\mu_P\left[\left(\left[\bar{\bar{\hat{M}}}\right]_{LHS}, [t_I, t_F]\right) \,\Big|\, (s^A, t_I)\right] = \sum_{[\hat{M}_1, \hat{M}_2, \cdots, \hat{M}_N] \in \left[\bar{\bar{\hat{M}}}\right]_{LHS}} \mu_P\Big[\big([\hat{M}_1, \hat{M}_2, \cdots, \hat{M}_N], [t_I, t_F]\big) \,\big|\, (s^A, t_I)\Big]$$

$$= \sum_{[\hat{M}_1, \hat{M}_2, \cdots, \hat{M}_N] \in \left[\bar{\bar{\hat{M}}}\right]_{LHS}} \int\cdots\int_{t_I=\tau_0<\tau_1<\cdots<\tau_N<\tau_{N+1}=t_F} d\tau_1 \cdots d\tau_N \left[\begin{array}{l} \left(\prod_{\nu=1}^{N} r(\hat{M}_\nu; s_{\nu-1}, \tau_\nu)\right) \\ \times \exp\left\{-\sum_{\nu=0}^{N}\int_{\tau_\nu}^{\tau_{\nu+1}} d\tau\, \delta R_X^{ID}(s_\nu, s^A, \tau)\right\}\Bigg|_{\substack{s_0=s^A,\\ \left\{\substack{\langle s_\nu|=\langle s_{\nu-1}|\hat{M}_\nu \\ for\ \nu=1,\ldots,N}\right\}}} \end{array}\right].$$

--- Eq.(SM-2.9)

Meanwhile, the right hand side of Eq.(SM-2.8) can be rewritten as:

$$\prod_{k=1}^{K}\mu_P\left[\left(\left[\hat{M}[k,1],...,\hat{M}[k,N_k]\right],[t_I,t_F]\right)\Big|\,(s^A,t_I)\right]$$

$$=\prod_{k=1}^{K}\left[\begin{array}{l}\displaystyle\int\cdots\int_{t_I=\tau(k,0)<\tau(k,1)<\cdots<\tau(k,N_k)<\tau(k,N_k+1)=t_F} d\tau(k,1)\cdots d\tau(k,N_k)\;\left(\prod_{i_k=1}^{N_k} r\left(\hat{M}[k,i_k];s_{i_k-1},\tau(k,i_k)\right)\right)\\ \displaystyle\times\exp\left\{-\sum_{i_k=0}^{N_k}\int_{\tau(k,i_k)}^{\tau(k,i_k+1)}d\tau\;\delta R_X^{ID}(s_{i_k},s^A,\tau)\right\}\Bigg|_{\langle s_0|=\langle s^A|,\;\left\{\begin{array}{l}\langle s_{i_k}|=\langle s_{i_k-1}|\hat{M}[k,i_k]\\ for\;\; i_k=1,...,N_k\end{array}\right\}}\end{array}\right].$$

--- Eq.(SM-2.10)

As we can see, Eq.(SM-2.9) and Eq.(SM-2.10) are quite similar. Each term in either expression is integration over $N\left(=\sum_{k=1}^{K}N_k\right)$ time variables. And each history, $[\hat{M}_1,\hat{M}_2,\cdots,\hat{M}_N]$, in Eq.(SM-2.9) is nothing other than a rearrangement of the equivalents of the events in the LHS, $\vec{\vec{\hat{M}}}=\left\{\left[\hat{M}[k,1],...,\hat{M}[k,N_k]\right]\right\}_{k=1,...,K}$. Therefore, if the following two equations hold, the ansatz Eq.(SM-2.8), will also hold.

(A) The equation between the domains of integration:

$$\sum_{[\hat{M}_1,\hat{M}_2,\cdots,\hat{M}_N]\in\left[\vec{\vec{\hat{M}}}\right]_{LHS}}\;\int\cdots\int_{t_I=\tau_0<\tau_1<\cdots<\tau_N<\tau_{N+1}=t_F} d\tau_1\cdots d\tau_N\,(...)$$

$$=\prod_{k=1}^{K}\left[\int\cdots\int_{t_I=\tau(k,0)<\tau(k,1)<\cdots<\tau(k,N_k)<\tau(k,N_k+1)=t_F} d\tau(k,1)\cdots d\tau(k,N_k)\right](...) .$$

(B) The equation between the integrands (*i.e.*, the probability densities):

$$\left(\prod_{i=1}^{N} r(\hat{M}_v;s_{v-1},\tau_v)\right)\exp\left\{-\sum_{v=0}^{N}\int_{\tau_v}^{\tau_{v+1}}d\tau\;\delta R_X^{ID}(s_v,s^A,\tau)\right\}\Bigg|_{s_0=s^A,\;\left\{\begin{array}{l}\langle s_v|=\langle s_{v-1}|\hat{M}_v\\ for\;\; v=1,...,N\end{array}\right\}}$$

$$=\prod_{k=1}^{K}\left[\begin{array}{l}\left(\prod_{i_k=1}^{N_k} r\left(\hat{M}[k,i_k];s_{i_k-1},\tau(k,i_k)\right)\right)\\ \displaystyle\times\exp\left\{-\sum_{i_k=0}^{N_k}\int_{\tau(k,i_k)}^{\tau(k,i_k+1)}dt\;\delta R_X^{ID}(s_{i_k},s^A,\tau)\right\}\Bigg|_{\langle s_0|=\langle s^A|,\;\left\{\begin{array}{l}\langle s_{i_k}|=\langle s_{i_k-1}|\hat{M}[k,i_k]\\ for\;\; i_k=1,...,N_k\end{array}\right\}}\end{array}\right].$$

(NOTE: Here, the equations were deliberately given in a rough manner, to aid the reader's intuitive understanding. Supplementary appendix SA-2.1 in Additional file 2 gives their mathematically rigorous forms.) Considering that a LHS equivalence class contains all possible local-order-conserving rearrangements of events in the representative LHS, equation (A) is intuitively very plausible. However, its mathematically rigorous proof is not so

straightforward, and is given in Supplementary appendix SA-2.2 in Additional file 2. Equation (B) might be intuitively less plausible, because of the differences in $\delta R_X^{ID}(s, s', \tau)$ on both sides. Nevertheless, we can prove that it also holds, provided that the following set of conditions is satisfied.

Condition (i): The rate of an indel event ($r(\hat{M}_v; s_{v-1}, \tau_v)$) is independent of the portion of the sequence state (s_{v-1}) outside of the region of the local history the event ($\hat{M}_v$) belongs to.
Condition (ii): The increment of the exit rate due to an indel event ($\delta R_X^{ID}(s_v, s_{v-1}, \tau)$, with $\langle s_v | = \langle s_{v-1} | \hat{M}_v$) is independent of the portion of the sequence state (s_{v-1}) outside of the region of the local history the event ($\hat{M}_v$) belongs to.

See Supplementary appendix SA-2.1 and SA-2.3 in Additional file 2 for the derivation of the mathematically rigorous version of this set of conditions. (For illustration, in Supplementary methods SM-3, the factorability of the probability will be examined for the simplest concrete LHS equivalence class (given in Figure 5).)

Once the factorability, Eq.(SM-2.8) (or Eq.(R6.2)), is established for each LHS equivalence class, it is relatively easy to prove the factorability for the total quotient for the PWA:

$$\tilde{\mu}_P\left[\left(\alpha(s^A,s^D),[t_I,t_F]\right)\middle|\,(s^A,t_I)\right] \equiv P\left[\left(\alpha(s^A,s^D),[t_I,t_F]\right)\middle|\,(s^A,t_I)\right]\Big/P\left[\left([\,],[t_I,t_F]\right)\middle|\,(s^A,t_I)\right]$$
$$= \sum_{\bar{\bar{\hat{M}}}\in\tilde{\Lambda}^{ID}\left[\alpha(s^A,s^D)\right]} \mu_P\left[\left(\left[\bar{\bar{\hat{M}}}\right]_{LHS},[t_I,t_F]\right)\middle|\,(s^A,t_I)\right],$$

--- Eq.(SM-2.11)
(which is equivalent to Eq.(R6.6) (or Eq.(SM-2.2)). Thanks to Eq.(SM-2.8) (or Eq.(R6.2)), each summand on the rightmost side is already factorized. One caveat, however, is that the set of local-history-accommodating regions could vary depending on the LHS, even if the resulting PWA is the same. This is because we are considering *all* indel histories, including non-parsimonious ones, that can yield the PWA, $\alpha(s^A,s^D)$. [NOTE: Some non-parsimonious indel histories contain local histories in between contiguous PASs, such as $\left[\hat{M}_I(x,l), \hat{M}_D(x+1, x+l)\right]$, which leave no traces of their own occurrences. They vary the set of regions accommodating local histories.] We will choose the maximum possible set of PASs in the given PWA, which separates the PWA into the finest potentially local-history-accommodating regions. [NOTE: Such a maximum set does not necessarily

consist of *all* PASs in the PWA. An example is given in subsection R8-3.] Let $\gamma_1, \gamma_2, ..., \gamma_{\kappa_{\max}}$ be such regions, where the number of regions, $\kappa_{\max}$, is uniquely determined by the PWA and the evolutionary model. Then, we can represent any

$\vec{\hat{\vec{M}}} = \left\{ \left[\hat{M}[k,1], ..., \hat{M}[k,N_k] \right] \right\}_{k=1,...,K} \in \tilde{\Lambda}^{ID}\left[\alpha(s^A, s^D) \right]$ as a vector with $\kappa_{\max}$ components:

$\vec{\hat{\vec{M}}} = \left(\vec{\hat{M}}[\gamma_1], \vec{\hat{M}}[\gamma_2], ..., \vec{\hat{M}}[\gamma_{\kappa_{\max}}] \right)$. Here $\vec{\hat{M}}[\gamma_\kappa] = \left[\hat{M}[k,1], ..., \hat{M}[k,N_k] \right]$ if the k th local history is confined in region γ_κ, or $\vec{\hat{M}}[\gamma_\kappa] = [\ ]$ (empty) if no events in the LHS occurred in γ_κ (Figure S1). Then, keeping $\mu_P\left[([\ ], [t_I, t_F]) \,|\, (s^A, t_I) \right] = 1$ in mind, the factorability, Eq.(R6.8), can be re-expressed as:

$$\mu_P\left[\left(\left[\vec{\hat{\vec{M}}} \right]_{LHS}, [t_I, t_F] \right) \middle| \, (s^A, t_I) \right] \;=\; \prod_{\kappa=1}^{\kappa_{\max}} \mu_P\left[\left(\vec{\hat{M}}[\gamma_\kappa], [t_I, t_F] \right) \middle| \, (s^A, t_I) \right] \;. \text{--- Eq.(SM-2.12)}$$

Now, consider the space $\tilde{\Lambda}^{ID}\left[\alpha(s^A, s^D) \right]$ itself. Any two different LHSs in this space differ at least by a local history in some γ_κ. Conversely, any given vector, $\left(\vec{\hat{M}}[\gamma_1], \vec{\hat{M}}[\gamma_2], ..., \vec{\hat{M}}[\gamma_{\kappa_{\max}}] \right)$, each of whose component ($\vec{\hat{M}}[\gamma_\kappa]$) is consistent with the PWA restricted in the region (γ_κ), defines a LHS in $\tilde{\Lambda}^{ID}\left[\alpha(s^A, s^D) \right]$. Thus, the set $\tilde{\Lambda}^{ID}\left[\alpha(s^A, s^D) \right]$ should be represented as a "direct product": $\tilde{\Lambda}^{ID}\left[\alpha(s^A, s^D) \right] = \mathop{\times}_{\kappa=1}^{\kappa_{\max}} \tilde{\Lambda}^{ID}\left[\gamma_\kappa; \alpha(s^A, s^D) \right]$, where $\tilde{\Lambda}^{ID}\left[\gamma_\kappa; \alpha(s^A, s^D) \right]$ denotes the set of local indel histories in γ_κ that can give rise to the sub-PWA of $\alpha(s^A, s^D)$ confined in γ_κ. Using this structure of $\tilde{\Lambda}^{ID}\left[\alpha(s^A, s^D) \right]$ and substituting Eq.(SM-2.12) for each $\vec{\hat{\vec{M}}} \in \tilde{\Lambda}^{ID}\left[\alpha(s^A, s^D) \right]$ into Eq.(SM-2.11), we finally get the desired factorization of the PWA probability quotient:

$$\tilde{\mu}_P\left[\left(\alpha(s^A, s^D), [t_I, t_F] \right) \middle| \, (s^A, t_I) \right] \;=\; \prod_{\kappa=1}^{\kappa_{\max}} \tilde{\mu}_P\left[\left(\tilde{\Lambda}^{ID}\left[\gamma_\kappa; \alpha(s^A, s^D) \right], [t_I, t_F] \right) \middle| \, (s^A, t_I) \right] \;.$$

--- Eq.(SM-2.13)

Here the multiplication factor,

$$\tilde{\mu}_P\left[\left(\tilde{\Lambda}^{ID}\left[\gamma_\kappa;\alpha(s^A,s^D)\right],[t_I,t_F]\right)\middle|\,(s^A,t_I)\right]\equiv\sum_{\bar{\hat{M}}[\gamma_\kappa]\in\tilde{\Lambda}^{ID}\left[\gamma_\kappa;\alpha(s^A,s^D)\right]}\mu_P\left[\left(\bar{\hat{M}}[\gamma_\kappa],[t_I,t_F]\right)\middle|\,(s^A,t_I)\right],$$

--- Eq.(SM-2.14)

(, which corresponds to Eq.(R6.8),) represents the total contribution to the PWA probability by *all* PWA-consistent local indel histories that can take place in γ_κ. Finally, the definition of the PWA probability quotient, Eq.(SM-2.11), transforms Eq.(SM-2.13) into the following key equation for the factorable *ab initio* PWA probability:

$$\begin{aligned}&P\left[\left(\alpha(s^A,s^D),[t_I,t_F]\right)\middle|\,(s^A,t_I)\right]\\&=P\left[\left([],[t_I,t_F]\right)\middle|\,(s^A,t_I)\right]\prod_{\kappa=1}^{\kappa_{\max}}\tilde{\mu}_P\left[\left(\tilde{\Lambda}^{ID}\left[\gamma_\kappa;\alpha(s^A,s^D)\right],[t_I,t_F]\right)\middle|\,(s^A,t_I)\right].\end{aligned}$$ --- Eq.(SM-2.15)

(It corresponds to Eq.(R6.7).)

SM-3. Factorability of probability of simplest LHS equivalence class

To illustrate how the factorization, Eq.(R6.2) (or Eq.(SM-2.8)), can be satisfied, here we will examine the probability of the simplest concrete LHS equivalence class, $\left[\left\{\left[\hat{M}_D(2,4)\right],\left[\hat{M}_I(6,3)\right]\right\}\right]_{LHS}$ (Figure 5). In this example, the two constituent indel histories, $\left[\hat{M}_D(2,4),\hat{M}_I(3,3)\right]$ and $\left[\hat{M}_I(6,3),\hat{M}_D(2,4)\right]$, share the ancestral state, $s^A=\left[1,2,3,4,5,6,7\right]$, and the descendant state, $s^D=\left[1,5,6,8,9,A,7\right]$. In addition, the histories have their own intermediate states, $\left\langle s_a\right|\equiv\left\langle s^A\right|\hat{M}_D(2,4)\ \left(=\left\langle[1,5,6,7]\right|\right)$ and $\left\langle s_b\right|\equiv\left\langle s^A\right|\hat{M}_I(6,3)\ \left(=\left\langle[1,2,3,4,5,6,8,9,A,7]\right|\right)$, respectively (Figure 5, panels a and b).

Using Eq.(SM-2.7), the probability quotient of the first indel history is given by:

$$\begin{aligned}&\mu_P\left[\left([\hat{M}_D(2,4),\hat{M}_I(3,3)],[t_I,t_F]\right)\middle|\,(s^A,t_I)\right]\\&=\iint_{t_I<\tau_1<\tau_2<t_F}d\tau_1\,d\tau_2\left[\begin{array}{l}r_D(2,4;s^A,\tau_1)\,r_I(3,3;s_a,\tau_2)\\\times\exp\left\{-\int_{\tau_1}^{\tau_2}d\tau\,\delta R_X^{ID}(s_a,s^A,\tau)-\int_{\tau_2}^{t_F}d\tau\,\delta R_X^{ID}(s^D,s^A,\tau)\right\}\end{array}\right]\\&=\iint_{t_I<\tau_1<\tau_2<t_F}d\tau_1\,d\tau_2\left[\begin{array}{l}r_D(2,4;s^A,\tau_1)\,r_I(3,3;s_a,\tau_2)\\\times\exp\left\{-\int_{\tau_2}^{t_F}d\tau\,\delta R_X^{ID}(s^D,s_a,\tau)-\int_{\tau_1}^{t_F}d\tau\,\delta R_X^{ID}(s_a,s^A,\tau)\right\}\end{array}\right].\end{aligned}$$ --- Eq.(SM-3.1)

To get the rightmost side, we used the identity: $\delta R_X^{ID}(s^D,s^A,\tau)=\delta R_X^{ID}(s^D,s_a,\tau)+\delta R_X^{ID}(s_a,s^A,\tau)$. Similarly, the quotient of the second indel history is:

$$\mu_P\left[\left([\hat{M}_I(6,3),\hat{M}_D(2,4)],[t_I,t_F]\right)\middle|\,(s^A,t_I)\right]$$

$$= \iint_{t_I<\tau_2<\tau_1<t_F} d\tau_2\, d\tau_1 \begin{bmatrix} r_I(6,3;s^A,\tau_2)\,r_D(2,4;s_b,\tau_1) \\ \times \exp\left\{-\int_{\tau_2}^{\tau_1} d\tau\ \delta R_X^{ID}(s_b,s^A,\tau) - \int_{\tau_1}^{t_F} d\tau\ \delta R_X^{ID}(s^D,s^A,\tau)\right\} \end{bmatrix}$$

$$= \iint_{t_I<\tau_2<\tau_1<t_F} d\tau_2\, d\tau_1 \begin{bmatrix} r_I(6,3;s^A,\tau_2)\,r_D(2,4;s_b,\tau_1) \\ \times \exp\left\{-\int_{\tau_2}^{t_F} d\tau\ \delta R_X^{ID}(s_b,s^A,\tau) - \int_{\tau_1}^{t_F} d\tau\ \delta R_X^{ID}(s^D,s_b,\tau)\right\} \end{bmatrix} \quad \text{. --- Eq.(SM-3.2)}$$

The total quotient of the subject LHS equivalence class is the summation of Eqs.(SM-3.1,2). We first notice that, *modulo differences of measure zero*, the union of the two domains of integration is a direct product:

$$\begin{aligned} &\left\{(\tau_1,\tau_2)\,\middle|\, t_I<\tau_1<\tau_2<t_F\right\} \cup \left\{(\tau_1,\tau_2)\,\middle|\, t_I<\tau_2<\tau_1<t_F\right\} \\ &= \left\{\tau_1\,\middle|\, t_I<\tau_1<t_F\right\} \times \left\{\tau_2\,\middle|\, t_I<\tau_2<t_F\right\} \end{aligned} \quad \text{. --- Eq.(SM-3.3)}$$

Thus, the total quotient can be factorized as:

$$\begin{aligned} &\mu_P\left[\left(\left[\{[\hat{M}_D(2,4)],[\hat{M}_I(6,3)]\}\right]_{LHS},[t_I,t_F]\right)\middle|\,(s^A,t_I)\right] \\ &= \left[\int_{t_I}^{t_F} d\tau_1\ r_D(2,4;s^A,\tau_1)\ \exp\left\{-\int_{\tau_1}^{t_F} d\tau\ \delta R_X^{ID}(s_a,s^A,\tau)\right\}\right] \\ &\quad\times \left[\int_{t_I}^{t_F} d\tau_2\ r_I(6,3;s^A,\tau_2)\exp\left\{-\int_{\tau_2}^{t_F} d\tau\ \delta R_X^{ID}(s_b,s^A,\tau)\right\}\right] \\ &= \mu_P\left[\left([\hat{M}_D(2,4)],[t_I,t_F]\right)\middle|\,(s^A,t_I)\right]\ \mu_P\left[\left([\hat{M}_I(6,3)],[t_I,t_F]\right)\middle|\,(s^A,t_I)\right] \end{aligned} ,$$

--- Eq.(SM-3.4)

provided that the following equations are satisfied:

$$r_D(2,4;s_b,\tau_1) = r_D(2,4;s^A,\tau_1) \quad , \qquad \text{--- Eq.(SM-3.5a)}$$

$$r_I(3,3;s_a,\tau_2) = r_I(6,3;s^A,\tau_2) \quad , \qquad \text{--- Eq.(SM-3.5b)}$$

$$\delta R_X^{ID}(s^D,s_b,\tau) = \delta R_X^{ID}(s_a,s^A,\tau) \quad , \qquad \text{--- Eq.(SM-3.5c)}$$

$$\delta R_X^{ID}(s^D,s_a,\tau) = \delta R_X^{ID}(s_b,s^A,\tau) \quad . \qquad \text{--- Eq.(SM-3.5d)}$$

Eq.(SM-3.5a) and Eq.(SM-3.5b) correspond to condition (i) in section R6 of Results and discussion. And, owing to the above definitions of s_a and s_b, and to the equations $\langle s^D| = \langle s_a|\hat{M}_I(3,3) = \langle s_b|\hat{M}_D(2,4)$, we see that Eq.(SM-3.5c) and Eq.(SM-3.5d) correspond to condition (ii) in section R6. Eq.(SM-3.4) is a concrete instance of the factorability, Eq.(R6.2) (or Eq.(SM-2.8)), when $\vec{\vec{\hat{M}}} = \left\{\left[\hat{M}_D(2,4)\right],\ \left[\hat{M}_I(6,3)\right]\right\}$. If you will, the factorability for more complex LHS equivalence classes could also be demonstrated concretely, although the

procedure becomes more cumbersome and lengthy. In any case, the proof can be generalized, as is fully described in Supplementary appendix SA-2 in Additional file 2.

SM-4. Factorability of multiple sequence alignment probability: details

As in section R7 of Results and discussion, here we formally calculate the *ab initio* probability of a MSA given a *rooted* phylogenetic tree, $T = (\{n\}_T, \{b\}_T)$, where $\{n\}_T$ is the set of all nodes of the tree, and $\{b\}_T$ is the set of all branches of the tree. We decompose the set of all nodes as: $\{n\}_T = \mathrm{N}^{IN}(T) + \mathrm{N}^X(T)$, where $\mathrm{N}^{IN}(T)$ is the set of all internal nodes and $\mathrm{N}^X(T) = \{n_1, ..., n_{N^X}\}$ is the set of all external nodes. (The $N^X \equiv |\mathrm{N}^X(T)|$ is the number of external nodes.) The root node plays an important role and will be denoted as $n^{Root}(T)$, or simply n^{Root}. Because the tree is rooted, each branch b is directed. Thus, let $n^A(b)$ denote the "ancestral node" on the upstream end of b, and let $n^D(b)$ denote the "descendant node" on the downstream end of b. Let $s(n) \in S^{II}$ be a sequence state at the node $n \in \{n\}_T$. Especially, let $s^A(b) \equiv s(n^A(b)) \in S^{II}$ denote a sequence state at $n^A(b)$ and let $s^D(b) \equiv s(n^D(b)) \in S^{II}$ denote a sequence state at $n^D(b)$. Finally, as mentioned in Background, we suppose that the branch lengths, $\{|b| \mid b \in \{b\}_T\}$, and the indel model parameters, $\{\Theta_{ID}(b)\}_T \equiv \{\Theta_{ID}(b) \mid b \in \{b\}_T\}$, are all given. Note that the model parameters $\Theta_{ID}(b)$ could vary depending on the branch, at least theoretically.

First, we extend the ideas proposed by [13,14,36] to each indel history along a tree, by regarding the indel history along a branch as a map (or a transformation) from the ancestral sequence state to the descendant sequence state, as follows. An indel history along a tree consists of indel histories along all branches of the tree that are interdependent, in the sense that the indel process of a branch b determines a sequence state $s^D(b)$ at its descendant node $n^D(b)$, on which the indel processes along its downstream branches depend. Thus, an indel history on a given root sequence state $s^{Root} = s(n^{Root}) \in S^{II}$ automatically determines the sequence states at all nodes, $\{s(n) \in S^{II} \ for\ {}^\forall n \in \{n\}_T\}$. Let $\tilde{\mathrm{H}}^{ID}(s_0) \equiv \bigcup_{N=0}^{\infty} \mathrm{H}^{ID}(N; s_0)$ (with $\mathrm{H}^{ID}(N; s_0)$ defined below Eq.(R4.6)) be the set of all indel histories along a time axis (or a branch) starting with state s_0. Then, each indel history, $\{\bar{\tilde{M}}(b)\}_T$, along tree T and

starting with s^{Root} can be specifically expressed as:

$$\left\{ \begin{array}{l} \vec{\hat{M}}(b) = \left[\hat{M}_1(b), ..., \hat{M}_{N(b)}(b) \right] \in \tilde{\mathrm{H}}^{ID}\left(s^A(b) \right) \quad and \\ \left\langle s^D(b) \right| = \left\langle s^A(b) \right| \hat{M}_1(b) \cdots \hat{M}_{N(b)}(b) \quad for \quad {}^\forall b \in \{b\}_T \end{array} \right| \left. s\left(n^{Root}(T) \right) = s^{Root} \right\} . \text{--- Eq.(SM-4.1)}$$

(It corresponds to Eq.(R7.1).) Here, the symbol, $\hat{M}_\nu(b)$, denotes the ν th event in the indel history along branch $b \in \{b\}_T$. The probability of the indel history, Eq.(SM-4.1), can be easily calculated. First, we already gave the conditional probability of an indel history during the time interval $[t_I, t_F]$, by Eq.(R4.7). Because we can correspond each branch $b \in \{b\}_T$ to a time interval $\left[t(n^A(b)), t(n^D(b)) \right]$ (with $t(n^D(b)) - t(n^A(b)) = |b|$), the probability of an indel history, $\vec{\hat{M}}(b) = \left[\hat{M}_1(b), ..., \hat{M}_{N(b)}(b) \right] \in \tilde{\mathrm{H}}^{ID}\left(s^A(b) \right)$, along a branch $b \in \{b\}_T$ is given by:

$$\begin{array}{l} P\left[\left(\vec{\hat{M}}(b), b \right) \middle| \, (s^A(b), n^A(b)) \right] \\ \equiv P\left[\left(\left[\hat{M}_1(b), \cdots, \hat{M}_{N(b)}(b) \right], \left[t(n^A(b)),\ t(n^D(b)) \right] \right) \middle| \left(s^A(b), t(n^A(b)) \right) \right] \Bigg|_{\Theta_{ID}(b)} \end{array} . \text{---Eq.(SM-4.2)}$$

(It corresponds to Eq.(R7.3).) Here we explicitly showed the branch-dependence of the model parameters. Using Eq.(SM-4.2) as a building block, the probability of the indel history along T, $\left\{ \vec{\hat{M}}(b) \right\}_T$, specified by Eq.(SM-4.1) (or Eq.(R7.1)), is given as:

$$P\left[\left\{ \vec{\hat{M}}(b) \right\}_T \middle| \left(s^{Root}, n^{Root} \right) \right] = \left(\prod_{b \in \{b\}_T} P\left[\left(\vec{\hat{M}}(b), b \right) \middle| \, (s^A(b), n^A(b)) \right] \right) \Bigg|_{\substack{s(n^{Root}) = s^{Root}, \\ \left\langle s^D(b) \right| = \left\langle s^A(b) \right| \hat{M}_1(b) \cdots \hat{M}_{N(b)}(b) \\ for \ {}^\forall b \in \{b\}_T}} .$$

--- Eq.(SM-4.3)

(It corresponds to Eq.(R7.2).)

In this way, we can calculate the probability of any indel history $\left\{ \vec{\hat{M}}(b) \right\}_T$ along tree T starting with a given root state, $s^{Root} \in S^{II}$.

Now, an important fact is that an indel history, along a tree starting with a root sequence state, uniquely yields a MSA, $\alpha[s_1, s_2, ..., s_{N^X}]$, among the sequences at the external nodes, $s_i = s(n_i) \in S^{II}$ ($n_i \in \mathrm{N}^X(T)$). [NOTE: Remember that the term "MSA" here means its homology structure.] However, the converse is not true. That is, a given MSA, $\alpha[s_1, s_2, ..., s_{N^X}]$, could result from a large number of alternative indel histories along a tree, even when starting with a given sequence state at the root. Moreover, there could be infinitely

many root states consistent with a given MSA. Here, let $\left(s^{Root}, \left\{\bar{\hat{M}}(b)\right\}_T\right)$ be a pair of a root state and an indel history along T starting with the state. And let $\tilde{\Psi}^{ID}\left[\alpha[s_1, s_2, ..., s_{N^X}]; T\right]$ be the set of all such pairs defined on T consistent with $\alpha[s_1, s_2, ..., s_{N^X}]$. Then, as the probability of a given PWA is expressed as Eq.(R4.9) supplemented with Eq.(R4.7), the probability of a given MSA under a given model setting (including T) should be expressed as:

$$P\left[\alpha[s_1, s_2, ..., s_{N^X}] \,|\, T\right] = \sum_{\substack{\left(s^{Root}, \left\{\bar{\hat{M}}(b)\right\}_T\right) \\ \in \tilde{\Psi}^{ID}\left[\alpha[s_1, s_2, ..., s_{N^X}]; T\right]}} P\left[\left(s^{Root}, n^{Root}\right)\right] P\left[\left\{\bar{\hat{M}}(b)\right\}_T \,\middle|\, \left(s^{Root}, n^{Root}\right)\right],$$

--- Eq.(SM-4.4)

which (, corresponding to Eq.(R7.4),) is supplemented with Eq.(SM-4.3) (or Eq.(R7.2)). Here, $P\left[\left(s^{Root}, n^{Root}\right)\right]$ is the probability of state s^{Root} at the root node (n^{Root}). (It may be interpreted as the prior in a Bayesian formalism.) If you will, Eq.(SM-4.4) supplemented with Eq.(SM-4.3) could be interpreted as the "perturbation expansion" of an *ab initio* MSA probability. To make this formal expansion formula more tractable, we consider the ancestral sequence states at all internal nodes, and let $\left\{s(n)\right\}_{\mathrm{N}^{IN}} \equiv \left\{s(n) \in S \,\middle|\, n \in \mathrm{N}^{IN}(T)\right\}$ denote a set of such ancestral states (or, more precisely, its equivalence class in the sense of endnote (h) (or 8)). To be consistent with a given MSA, the ancestral states must satisfy the "phylogenetic correctness" condition in each MSA column [37,38]. [NOTE: The "phylogenetic correctness" condition guarantees that the sites aligned in a MSA column should share an ancestry. The condition could be rephrased as: "if a site corresponding to the column is present at two points in the phylogenetic tree, the site must also be present all along the shortest path connecting the two points."] As long as the condition is fulfilled in all MSA columns, however, any set of states must be allowed. So, let $\Sigma\left[\alpha[s_1, s_2, ..., s_{N^X}]; \left\{n \in \mathrm{N}^{IN}(T)\right\}; T\right]$ be the set of all $\left\{s(n)\right\}_{\mathrm{N}^{IN}}$'s consistent with $\alpha[s_1, s_2, ..., s_{N^X}]$ (and tree T). Then, the aforementioned set, $\tilde{\Psi}^{ID}\left[\alpha[s_1, s_2, ..., s_{N^X}]; T\right]$, can be uniquely decomposed into the following direct sum:

$$\tilde{\Psi}^{ID}\left[\alpha[s_1,s_2,...,s_{N^X}];T\right] = \bigcup_{\substack{\{s(n)\}_{\mathrm{N}^{IN}} \\ \in\ \Sigma\left[\alpha[s_1,s_2,...,s_{N^X}];\left\{n\in\mathrm{N}^{IN}(T)\right\};T\right]}} \Psi^{ID}\left[\alpha[s_1,s_2,...,s_{N^X}];\left\{s(n)\right\}_{\mathrm{N}^{IN}};T\right] \ .$$

--- Eq.(SM-4.5)

Here, $\Psi^{ID}\left[\alpha[s_1,s_2,...,s_{N^X}];\left\{s(n)\right\}_{\mathrm{N}^{IN}};T\right]$ denotes the set of indel histories along T consistent with both the MSA ($\alpha[s_1,s_2,...,s_{N^X}]$) and the ancestral sequence states ($\left\{s(n)\right\}_{\mathrm{N}^{IN}}$). Substituting Eq.(SM-4.5) into Eq.(SM-4.4), we have:

$$P\left[\alpha[s_1,s_2,...,s_{N^X}]\,|\,T\right] = \sum_{\substack{\{s(n)\}_{\mathrm{N}^{IN}} \\ \in\ \Sigma\left[\alpha[s_1,s_2,...,s_{N^X}];\left\{n\in\mathrm{N}^{IN}(T)\right\};T\right]}} P\left[\alpha[s_1,s_2,...,s_{N^X}];\left\{s(n)\right\}_{\mathrm{N}^{IN}}\,|\,T\right] \ .$$

--- Eq.(SM-4.6)

(It corresponds to Eq.(R7.5).) Here,

$$P\left[\alpha[s_1,s_2,...,s_{N^X}];\left\{s(n)\right\}_{\mathrm{N}^{IN}}\,|\,T\right]$$

$$\equiv \sum_{\substack{\left(s^{Root},\left\{\vec{\hat{M}}(b)\right\}_T\right) \\ \in\Psi^{ID}\left[\alpha[s_1,s_2,...,s_{N^X}];\left\{s(n)\right\}_{\mathrm{N}^{IN}};T\right]}} P\left[\left(s^{Root},n^{Root}\right)\right]P\left[\left\{\vec{\hat{M}}(b)\right\}_T\,\middle|\,\left(s^{Root},n^{Root}\right)\right]$$

--- Eq.(SM-4.7)

is the probability of simultaneously getting $\alpha[s_1,s_2,...,s_{N^X}]$ and $\left\{s(n)\right\}_{\mathrm{N}^{IN}}$. Thus, all terms in Eq.(SM-4.7) share the same homology structure among sequence states at all nodes. Especially, the sequence states at internal nodes have homology structures (with states at other nodes) fixed for respective nodes. And each history consists of indel histories along branches consistent with each other (as in Eq.(SM-4.1) (or Eq.(R7.1))). This, in conjunction with the fact that the states at the internal nodes having node-fixed homology structures could be used as "anchors," the history component of $\Psi^{ID}\left[\alpha[s_1,s_2,...,s_{N^X}];\left\{s(n)\right\}_{\mathrm{N}^{IN}};T\right]$ could be *vertically* decomposed into a direct product:

$$\Psi^{ID}\left[\alpha[s_1,s_2,...,s_{N^X}];\left\{s(n)\right\}_{\mathrm{N}^{IN}};T\right] = \left(s^{Root}, \mathop{\times}_{b\in\{b\}_T} \tilde{\mathrm{H}}^{ID}\left[\alpha\left(s^A(b),s^D(b)\right)\right]\right). \quad \text{--- Eq.(SM-4.8)}$$

Here, $s^A(b)$ and $s^D(b)$ for each branch are proper elements in the set of (the equivalence classes of) states, $\left\{s_i\right\}_{i=1,...,N^X} \cup \left\{s(n)\right\}_{\mathrm{N}^{IN}}$. (All pairs, $\left(s^{Root},\left\{\vec{\hat{M}}(b)\right\}_T\right)$'s, share the root state.)

Substituting Eq.(SM-4.3) and Eq.(SM-4.8) into Eq.(SM-4.7), and lumping together the terms along each branch using Eq.(R4.9), we finally get:

$$P\left[\alpha[s_1, s_2, ..., s_{N^X}]; \{s(n)\}_{\mathrm{N}^{IN}} \mid T\right]$$
$$= P\left[\left(s^{Root}, n^{Root}\right)\right] \prod_{b \in \{b\}_T} P\left[(\alpha(s^A(b), s^D(b)), b) \,\middle|\, (s^A(b), n^A(b))\right] \cdot \quad \text{--- Eq.(SM-4.9)}$$

(It corresponds to Eq.(R7.6).) Here,

$$P\left[(\alpha(s^A(b), s^D(b)), b) \,\middle|\, (s^A(b), n^A(b))\right]$$
$$\equiv P\left[\left(\alpha(s^A(b), s^D(b)), \left[t\left(n^A(b)\right), t\left(n^D(b)\right)\right]\right) \,\middle|\, \left(s^A(b), t\left(n^A(b)\right)\right)\right]\Big|_{\Theta_{ID}(b)} \quad \text{--- Eq.(SM-4.10)}$$

(, which corresponds to Eq.(R7.7),) is the probability of the ancestor-descendant PWA along branch b. This Eq.(SM-4.9) is basically the expression proposed in [13,14], and we demonstrated in effect that their proposal also holds even with a genuine stochastic evolutionary model. Usually, Eq.(SM-4.6) supplemented with Eq.(SM-4.9) is much more tractable than Eq.(SM-4.4) supplemented with Eq.(SM-4.3), because of the two reasons. (1) Usually, it is not the indel history (along the tree) but (the homology structure of) the set of ancestral sequence states that is inferred from a given MSA. (2) The probability of each indel history along the tree (Eq.(SM-4.3)) is not factorable in general, whereas Eq.(SM-4.9) is a product of PWA probabilities, each of which should be factorable if the conditions (i) and (ii) in section R6 are satisfied.

Now, we seek to factorize the *ab initio* MSA probability into a form somewhat similar to Eq.(R6.7) for the *ab initio* PWA probability. In subsection 4.2 of [32], we did so using the history-based expansion of the MSA probability (*i.e.*, Eq.(SM-4.4) supplemented with Eq.(SM-4.3)). Here, we will use the ancestral-state-based expansion (*i.e.*, Eq.(SM-4.6) supplemented with Eq.(SM-4.9)), as was only briefly sketched at the bottom of subsection 4.2 of [32]. In a MSA, gapless columns play almost the same role as PASs in a PWA. Because of the aforementioned "phylogenetic correctness" condition, a gapless column indicates that the site in question existed all across the phylogenetic tree, and thus that no indel event hit or pierce the site. Therefore, gapless columns will partition a MSA into regions each of which accommodates a local subset of every global history. Analogously to the argument above Eq.(SM-2.12), let $C_1, C_2, ..., C_{\mathrm{K}_{\max}}$ be the maximum possible set of such regions determined by a given MSA ($\alpha[s_1, s_2, ..., s_{N^X}]$) and a model setting (including tree T). (As argued there, all gapless columns are not necessarily needed to delimit the regions.) Meanwhile, if the conditions (i) and (ii) in section R6 are satisfied, each factor in the product in Eq.(SM-4.9) can be factorized as in Eq.(R6.7):

$$P\left[(\alpha(s^A(b),s^D(b)),b)\,\middle|\,(s^A(b),n^A(b))\right]$$

$$=P\left[([],b)\,\middle|\,(s^A(b),n^A(b))\right]\prod_{\kappa_b=1}^{\kappa_{\max}(b)}\tilde{\mu}_P\left[\left(\tilde{\Lambda}^{ID}\left[\gamma_{\kappa_b}(b);\alpha(s^A(b),s^D(b))\right],b\right)\,\middle|\,(s^A(b),n^A(b))\right].$$

--- Eq.(SM-4.11)

Here we used the notation that helps easily remind the dependence on the branch (b). Especially, $\left\{\gamma_{\kappa_b}(b)\right\}_{\kappa_b=1,\ldots,\kappa_{\max}(b)}$ denotes the maximum set of regions accommodating local indel histories along b consistent with the PWA, $\alpha(s^A(b),s^D(b))$ (Figure S2). Because the set of gapless columns delimiting $\left\{C_{\mathrm{K}}\right\}_{\mathrm{K}=1,\ldots,\mathrm{K}_{\max}}$ defines a subset of PASs in $\alpha(s^A(b),s^D(b))$ delimiting $\left\{\gamma_{\kappa_b}(b)\right\}_{\kappa_b=1,\ldots,\kappa_{\max}(b)}$, each C_{K} should encompass at least one $\gamma_{\kappa_b}(b)$ (Figure S2).

Thus, Eq.(SM-4.9) supplemented with Eq.(SM-4.11) could be rearranged as:

$$P\left[\alpha[s_1,s_2,\ldots,s_{N^X}];\left\{s(n)\right\}_{\mathrm{N}^{IN}}\,\middle|\,T\right]$$

$$=P\left[\left(s^{Root},n^{Root}\right)\right]\left(\prod_{b\in\{b\}_T}P\left[([],b)\,\middle|\,(s^A(b),n^A(b))\right]\right)\left(\prod_{\mathrm{K}=1}^{\mathrm{K}_{\max}}\mathrm{M}_P\left[\alpha[s_1,s_2,\ldots,s_{N^X}];\left\{s(n)\right\}_{\mathrm{N}^{IN}};C_{\mathrm{K}}\,\middle|\,T\right]\right).$$

--- Eq.(SM-4.12)

Here, the "raw" multiplication factor contributed from the region, C_{K}, is given by:

$$\mathrm{M}_P\left[\alpha[s_1,s_2,\ldots,s_{N^X}];\left\{s(n)\right\}_{\mathrm{N}^{IN}};C_{\mathrm{K}}\,\middle|\,T\right]$$

$$\equiv\prod_{b\in\{b\}_T}\left\{\prod_{\gamma_{\kappa_b}(b)\subseteq C_{\mathrm{K}}}\tilde{\mu}_P\left[\left(\tilde{\Lambda}^{ID}\left[\gamma_{\kappa_b}(b);\alpha(s^A(b),s^D(b))\right],b\right)\,\middle|\,(s^A(b),n^A(b))\right]\right\}.\text{ --- Eq.(SM-4.13)}$$

To factorize the total probability of $\alpha[s_1,s_2,\ldots,s_{N^X}]$, Eq.(SM-4.6) (or Eq.(R7.5)), we need to consider multiple sets of ancestral states. For this purpose, we introduce a "reference" root sequence state, s_0^{Root}. It can be anything, as long as it is the state at the root consistent with $\alpha[s_1,s_2,\ldots,s_{N^X}]$. Technically, one good candidate for s_0^{Root} would be a root state obtained by applying the Dollo parsimony principle [39] to each column of the MSA, because it is arguably the most readily available state that satisfies the phylogenetic correctness condition along the entire MSA. Given a reference, s_0^{Root}, each ancestral state $s^A(b)$ should differ from s_0^{Root} only within some C_{K}'s. Moreover, the condition (ii) in section R6 guarantees that the impacts of their differences within separate C_{K}'s on the exit rate should be independent of each other. Thus, we have:

$R_X^{ID}(s^A(b),\ t) = R_X^{ID}(s_0^{Root},\ t) + \sum_{\mathrm{K}=1}^{\mathrm{K}_{\max}} \delta R_X^{ID}(s^A(b),\ s_0^{Root},\ t)[C_{\mathrm{K}}]$, ---Eq.(SM-4.14)

where $\delta R_X^{ID}(s^A(b),\ s_0^{Root},\ t)[C_{\mathrm{K}}]$ is the increment of the exit rate due to the difference between $s^A(b)$ and s_0^{Root} within the region C_{K}. Remembering that $P\left[([\,], b) \,\middle|\, (s^A(b), n^A(b))\right] = \exp\left(-\int_{t(n^A(b))}^{t(n^D(b))} d\tau\ R_X^{ID}(s^A(b), \tau)\right)$, the product in the middle of the right hand side of Eq.(SM-4.12) can be rewritten as:

$$\prod_{b \in \{b\}_T} P\left[([\,], b) \,\middle|\, (s^A(b), n^A(b))\right]$$

$$= P\left[\{[\,]\}_T \,\middle|\, \left(s_0^{Root}, n^{Root}\right)\right] \prod_{\mathrm{K}=1}^{\mathrm{K}_{\max}} \left\{ \exp\left(- \sum_{b \in \{b\}_T} \int_{t(n^A(b))}^{t(n^D(b))} d\tau\ \delta R_X^{ID}(s^A(b),\ s_0^{Root},\ \tau)[C_{\mathrm{K}}] \right) \right\} .$$

--- Eq.(SM-4.15)

Here, $P\left[\{[\,]\}_T \,\middle|\, \left(s_0^{Root}, n^{Root}\right)\right] = \exp\left(-\sum_{b \in \{b\}_T} \int_{t(n^A(b))}^{t(n^D(b))} d\tau\ R_X^{ID}(s_0^{Root}, \tau)\right)$ is the probability that the sequence underwent no indel all across the tree (T), conditioned on that the state was s_0^{Root} at the root. The remaining factor is the (prior) probability of the state at the root, $P\left[\left(s^{Root}, n^{Root}\right)\right]$. We will impose a third condition:

Condition (iii):

$$P\left[\left(s^{Root}, n^{Root}\right)\right] = P\left[\left(s_0^{Root}, n^{Root}\right)\right] \prod_{\mathrm{K}=1}^{\mathrm{K}_{\max}} \mu_P\left[s^{Root}, s_0^{Root}, n^{Root}; C_{\mathrm{K}}\right] . \text{--- Eq.(SM-4.16)}$$

(It corresponds to Eq.(R7.8).) Here the multiplication factor, $\mu_P\left[s^{Root}, s_0^{Root}, n^{Root}; C_{\mathrm{K}}\right]$, represents the change in the state probability at the root due to the difference between s^{Root} and s_0^{Root} within C_{K}. This equation holds, *e.g.*, when $P\left[\left(s^{Root}, n^{Root}\right)\right]$ is a geometric distribution or a uniform distribution of the root sequence length, $L(s^{Root})$. [NOTE: HMMs commonly use geometric distributions of sequence lengths. The uniform distribution may be a good approximation if we can assume that the ancestral sequence was sampled randomly from a chromosome of length L_C. In this case, the distribution of the sequence length $L(s)\ (<< L_C)$ would be proportional to $\left(1 - (L(s) - 1)/L_C\right) \approx 1$.] Using Eqs.(SM-4.15,16), Eq.(SM-4.12) can be rewritten as:

$$P\left[\alpha[s_1, s_2, ..., s_{N^X}]; \{s(n)\}_{\mathrm{N}^{IN}} \mid T\right]$$
$$= P\left[\left(s_0^{Root}, n^{Root}\right)\right] P\left[\{[\,]\}_T \mid \left(s_0^{Root}, n^{Root}\right)\right] \left(\prod_{\mathrm{K}=1}^{\mathrm{K}_{\max}} \breve{\mathrm{M}}_P \left[\alpha[s_1, s_2, ..., s_{N^X}]; \{s(n)\}_{\mathrm{N}^{IN}}; s_0^{Root}; C_{\mathrm{K}} \mid T\right] \right).$$

--- Eq.(SM-4.17)

Here, the "augmented" multiplication factor contributed from C_{K} is defined as:

$$\breve{\mathrm{M}}_P \left[\alpha[s_1, s_2, ..., s_{N^X}]; \{s(n)\}_{\mathrm{N}^{IN}}; s_0^{Root}; C_{\mathrm{K}} \mid T\right]$$
$$\equiv \mathrm{M}_P \left[\alpha[s_1, s_2, ..., s_{N^X}]; \{s(n)\}_{\mathrm{N}^{IN}}; C_{\mathrm{K}} \mid T\right] \mu_P \left[s\left(n^{Root}\right), s_0^{Root}, n^{Root}; C_{\mathrm{K}}\right] \quad \text{. --- Eq.(SM-4.18)}$$
$$\times \exp\left(- \sum_{b \in \{b\}_T} \int_{t\left(n^A(b)\right)}^{t\left(n^D(b)\right)} d\tau \, \delta R_X^{ID}(s^A(b), \; s_0^{Root}, \; \tau)[C_{\mathrm{K}}] \right)$$

Substituting Eq.(SM-4.17) into Eq.(SM-4.6) (or Eq.(R7.5)), we are just a step short of the complete factorization. The final step is the "decomposition" of the space, $\Sigma\left[\alpha[s_1,s_2,...,s_{N^X}]; \{n \in \mathrm{N}^{IN}(T)\}; T\right]$, each of whose elements is a set of MSA-consistent ancestral states at all internal nodes. For this purpose, we use s_0^{Root} once again, and define $\Delta_\Sigma\left[s_0^{Root}; \alpha[s_1,s_2,...,s_{N^X}]; \{n \in \mathrm{N}^{IN}(T)\}; T\right]$ as the space of *deviations* of MSA-consistent internal states from s_0^{Root}. As argued above, the deviations of ancestral states from s_0^{Root} come only from C_{K}'s (with $\mathrm{K} = 1,...,\mathrm{K}_{\max}$), and deviations from different C_{K}'s behave independently from each other (thanks to the delimiting gapless columns and conditions (i) and (ii)). Thus, we get the direct-product structure:

$$\Delta_\Sigma\left[s_0^{Root}; \alpha[s_1,s_2,...,s_{N^X}]; \{n \in \mathrm{N}^{IN}(T)\}; T\right]$$
$$= \bigtimes_{\mathrm{K}=1}^{\mathrm{K}_{\max}} \Delta_\Sigma\left[C_{\mathrm{K}}; s_0^{Root}; \alpha[s_1,s_2,...,s_{N^X}]; \{n \in \mathrm{N}^{IN}(T)\}; T\right] \quad \text{. --- Eq.(SM-4.19)}$$

Here, $\Delta_\Sigma\left[C_{\mathrm{K}}; s_0^{Root}; \alpha[s_1,s_2,...,s_{N^X}]; \{n \in \mathrm{N}^{IN}(T)\}; T\right]$ is the space of deviations within C_{K}.

In Eq.(SM-4.17), all the *absolute* dependences on s_0^{Root} were factored out of the product over K. Thus, in Eq.(SM-4.6) (or Eq.(R7.5)), the summation over $\Sigma\left[\alpha[s_1,s_2,...,s_{N^X}]; \{n \in \mathrm{N}^{IN}(T)\}; T\right]$ is reduced to the summation over

$\Delta_\Sigma\left[s_0^{Root}; \alpha[s_1,s_2,...,s_{N^X}]; \{n \in \mathrm{N}^{IN}(T)\}; T\right]$. Exploiting Eq.(SM-4.17) and Eq.(SM-4.19), Eq.(SM-4.6) can be re-expressed into the final factorized form:

$$P\left[\alpha[s_1, s_2,..., s_{N^X}] \mid T\right] = P_0\left[s_0^{Root} \mid T\right] \prod_{\mathrm{K}=1}^{\mathrm{K}_{\max}} \tilde{\breve{\mathrm{M}}}_P\left[\alpha[s_1, s_2,..., s_{N^X}]; s_0^{Root}; C_{\mathrm{K}} \mid T\right] \text{ . --- Eq.(SM-4.20)}$$

(It corresponds to Eq.(R7.9).) Here,

$$P_0\left[s_0^{Root} \mid T\right] \equiv P\left[\left(s_0^{Root}, n^{Root}\right)\right] P\left[\{[\,]\}_T \,\middle|\, \left(s_0^{Root}, n^{Root}\right)\right]. \text{ --- Eq.(SM-4.21)}$$

(, which corresponds to Eq.(R7.10),) is the probability of having a sequence state s_0^{Root} that has been intact all across tree T, and

$$\tilde{\breve{\mathrm{M}}}_P\left[\alpha[s_1, s_2,..., s_{N^X}]; s_0^{Root}; C_{\mathrm{K}} \mid T\right]$$
$$\equiv \sum_{\substack{\left\{s(n)-s_0^{Root}\right\}_{\mathrm{N}^{IN}}[C_{\mathrm{K}}] \\ \in \Lambda_\Sigma\left[C_{\mathrm{K}};\, s_0^{Root};\, \alpha[s_1,s_2,...,s_{N^X}];\, \left\{n \in \mathrm{N}^{IN}(T)\right\};\, T\right]}} \breve{\mathrm{M}}_P\left[\alpha[s_1, s_2,..., s_{N^X}]; \{s(n)\}_{\mathrm{N}^{IN}}; s_0^{Root}; C_{\mathrm{K}} \mid T\right]. \text{ ---Eq.(SM-4.22)}$$

is the multiplication factor contributed from all MSA-consistent local indel histories (along T) confined in C_{K}. [NOTE: $\tilde{\breve{\mathrm{M}}}_P\left[\alpha[s_1, s_2,..., s_{N^X}]; s_0^{Root}; C_{\mathrm{K}} \mid T\right]$ given in Eq.(SM-4.22) should be equivalent to $\tilde{\breve{\mathrm{M}}}_P\left[\tilde{\Lambda}_\Psi^{ID}\left[C_{\mathrm{K}}; \alpha[s_1,s_2,...,s_{N^X}]; T\right] \mid T\right]$ given in Eq.(4.2.9c) of [32], although the two expressions may appear quite different at first glance.] In Eq.(SM-4.22), we let $\left\{s(n)-s_0^{Root}\right\}_{\mathrm{N}^{IN}}[C_{\mathrm{K}}]$ denote the portion of the deviation of $\{s(n)\}_{\mathrm{N}^{IN}}$ from s_0^{Root} confined in C_{K}.

Supplementary figures (with legends)

a Global indel history

$$\langle s_I |$$
$$\Downarrow$$
$$\langle s_1 | = \langle s_I | \hat{M}_D(4,4)$$
$$\Downarrow$$
$$\langle s_2 | = \langle s_1 | \hat{M}_I(7,2)$$
$$\Downarrow$$
$$\langle s_3 | = \langle s_2 | \hat{M}_D(3,4)$$
$$\Downarrow$$
$$\langle s_4 | = \langle s_3 | \hat{M}_I(4,1)$$
$$\Downarrow$$
$$\langle s_5 | = \langle s_4 | \hat{M}_I(8,1)$$
$$\Downarrow$$
$$\langle s_F | = \langle s_5 | \hat{M}_D(5,5)$$

b Resulting MSA (in S^{II}) and local regions

I		1	2	3	4	5	6	7		8	-	-	-	9
1		1	2	3	-	5	6	7		8	-	-	-	9
2		1	2	3	-	5	6	7		8	A	B	-	9
3		1	2	-	-	-	6	7		8	A	B	-	9
4		1	2	-	-	-	6	7	C	8	A	B	-	9
5		1	2	-	-	-	6	7	C	8	A	B	D	9
F		1	2	-	-	-	6	7		8	A	B	D	9

γ_1 γ_2 γ_3 γ_4 γ_5 γ_6 γ_7

c LHS(original representation):

$$\vec{\vec{\hat{M}}} = \left\{ \vec{\hat{M}}[k] \equiv \left[\vec{\hat{M}}[k,1], \ldots, \vec{\hat{M}}[k,N_k] \right] \right\}_{k=1,2,3}$$

with

$$\vec{\hat{M}}[1] = \left[\hat{M}_D(4,4), \hat{M}_D(3,4) \right] = \left[\hat{M}'_D(4,4), \hat{M}'_D(3,4) \right],$$
$$\vec{\hat{M}}[2] = \left[\hat{M}_I(7,1), \hat{M}_D(8,8) \right] = \left[\hat{M}'_I(4,1), \hat{M}'_D(5,5) \right],$$
$$\vec{\hat{M}}[3] = \left[\hat{M}_I(8,2), \hat{M}_I(10,1) \right] = \left[\hat{M}'_I(7,2), \hat{M}'_I(8,1) \right].$$

d LHS (vector representation):

$$\vec{\vec{\hat{M}}} = \left(\vec{\hat{M}}[\gamma_1], \vec{\hat{M}}[\gamma_2], \ldots, \vec{\hat{M}}[\gamma_7] \right)$$

with

$$\vec{\hat{M}}[\gamma_1] = \vec{\hat{M}}[\gamma_2] = \vec{\hat{M}}[\gamma_4] = \vec{\hat{M}}[\gamma_7] = [\,],$$
$$\vec{\hat{M}}[\gamma_3] = \vec{\hat{M}}[1], \; \vec{\hat{M}}[\gamma_5] = \vec{\hat{M}}[2], \; \vec{\hat{M}}[\gamma_6] = \vec{\hat{M}}[3].$$

Figure S1. "Vector" representation of example LHS along time interval.
a An example global indel history, consisting of six indel events and seven resulting sequence states (including the initial state s_I). **b** The resulting MSA among the sequence states that the indel history went through. The boldface letters in the leftmost column indicate the sequence states in the global history (panel **a**). The 1-9,A-D in the cells are the ancestry indices of the sites. The cells shaded in magenta and red represent the sites to be deleted. Those shaded in cyan and blue represent the inserted sites. And those shaded in yellow represent the inserted sites to be deleted. Below the MSA, the bottom curly brackets indicate the regions γ_κ ($\kappa = 3,5,6$ in this example) that actually accommodate local indel histories. And the yellow wedges indicate the regions γ_κ ($\kappa = 1,2,4,7$ in this example) that can potentially accommodate local indel histories, but that actually do not. In this example, $K = 3$, $N_1 = N_2 = N_3 = 2$, and $\kappa_{\max} = 7$. **c** The original representation of the local history set (LHS).

In each defining equation for $\bar{\hat{M}}[k]$ $(k=1,2,3)$, the expression in the middle is the local history represented by its action on the initial state (s_I). And on the right-most side is the representation by the actual indel events in the global history (in panel **a**), where the prime indicates that each defining event is equivalent to but not necessarily equal to the corresponding event in the global history. **d** The vector representation of the LHS. The "[ ]" denotes an empty local history, in which no indel event took place. The figure was adapted from Figure 10 of [32].

a Global indel history

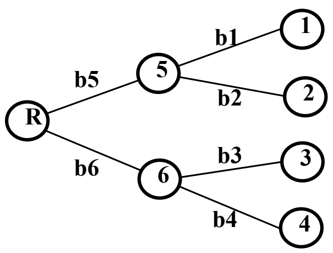

$\vec{\hat{M}}(b5)=\left[\hat{M}_I(5,1)\right]$,
$\vec{\hat{M}}(b6)=\left[\hat{M}_I(10,1)\right]$,
$\vec{\hat{M}}(b1)=\left[\hat{M}_D(11,11),\ \hat{M}_D(4,5)\right]$,
$\vec{\hat{M}}(b2)=\left[\hat{M}_I(5,1)\right]$,
$\vec{\hat{M}}(b3)=[\ ]$,
$\vec{\hat{M}}(b4)=\left[\hat{M}_D(4,4)\right]$.

b Resulting MSA (in S^{II}) and local regions

R		1	2	3	4	5	-	-	6	7	8	9	A	-	B	C
5		1	2	3	4	5	-	E	6	7	8	9	A	-	B	C
6		1	2	3	4	5	-	-	6	7	8	9	A	D	B	C
1		1	2	3	-	-	-	E	6	7	8	9	-	-	B	C
2		1	2	3	4	5	F	E	6	7	8	9	A	-	B	C
3		1	2	3	4	5	-	-	6	7	8	9	A	D	B	C
4		1	2	3	-	5	-	-	6	7	8	9	A	D	B	C

C_1 C_2 C_3 C_4 C_5 C_6 C_7 C_8 C_9 C_{10}

c LHSs along branches (vector representation):

R		1	2	3	4	5	-	6	7	8	9	A	B	C
5		1	2	3	4	5	E	6	7	8	9	A	B	C

$\gamma_1(b5)\ \gamma_2(b5)\ \gamma_3(b5)\ \gamma_4(b5)\ \gamma_5(b5)\ \gamma_6(b5)\ \gamma_7(b5)\ \gamma_8(b5)\ \gamma_9(b5)\ \gamma_{10}(b5)\ \gamma_{11}(b5)\ \gamma_{12}(b5)\ \gamma_{13}(b5)$

$$\vec{\vec{\hat{M}}}(b5)=\left(\vec{\hat{M}}[\gamma_1(b5)],\dots,\vec{\hat{M}}[\gamma_{13}(b5)]\right)=\left([],[],[],[],[],\left[\hat{M}_I(5,1)\right],[],[],[],[],[],[],[]\right).$$

5		1	2	3	4	5	E	6	7	8	9	A	B	C
1		1	2	3	-	-	E	6	7	8	9	-	B	C

$\gamma_1(b1)\ \gamma_2(b1)\ \gamma_3(b1)\ \gamma_4(b1)\ \gamma_5(b1)\ \gamma_6(b1)\ \gamma_7(b1)\ \gamma_8(b1)\ \gamma_9(b1)\ \gamma_{10}(b1)\ \gamma_{11}(b1)$

$$\vec{\vec{\hat{M}}}(b1)=\left(\vec{\hat{M}}[\gamma_1(b1)],\dots,\vec{\hat{M}}[\gamma_{11}(b1)]\right)=\left([],[],[],\left[\hat{M}_D(4,5)\right],[],[],[],[],\left[\hat{M}_D(11,11)\right],[],[]\right).$$

Similarly,

$$\vec{\vec{\hat{M}}}(b6)=\left(\vec{\hat{M}}[\gamma_1(b6)],\dots,\vec{\hat{M}}[\gamma_{13}(b6)]\right)=\left([],[],[],[],[],[],[],[],[],[],\left[\hat{M}_I(10,1)\right],[],[]\right),$$

$$\vec{\vec{\hat{M}}}(b2)=\left(\vec{\hat{M}}[\gamma_1(b2)],\dots,\vec{\hat{M}}[\gamma_{14}(b2)]\right)=\left([],[],[],[],[],\left[\hat{M}_I(5,1)\right],[],[],[],[],[],[],[],[]\right),$$

$$\vec{\vec{\hat{M}}}(b3)=\left(\vec{\hat{M}}[\gamma_1(b3)],\dots,\vec{\hat{M}}[\gamma_{14}(b3)]\right)=([],[],[],[],[],[],[],[],[],[],[],[],[],[]),$$

$$\vec{\vec{\hat{M}}}(b4)=\left(\vec{\hat{M}}[\gamma_1(b4)],\dots,\vec{\hat{M}}[\gamma_{13}(b4)]\right)=\left([],[],[],\left[\hat{M}_D(4,4)\right],[],[],[],[],[],[],[],[],[]\right).$$

d LHS along the tree (vector representation):

$$\left\{\vec{\vec{\hat{M}}}(b)\right\}_T=\left(\left\{\vec{\vec{\hat{M}}}(b)\right\}_T[C_1],\dots,\left\{\vec{\vec{\hat{M}}}(b)\right\}_T[C_{10}]\right),$$

with

$$\left\{\vec{\vec{\hat{M}}}(b)\right\}_T[C_K]=\{\ \}\quad for\quad K=1,2,3,5,6,7,9,10,$$
$$\left\{\vec{\vec{\hat{M}}}(b)\right\}_T[C_4]=\left\{\vec{\hat{M}}[\gamma_6(b5)]=\left[\hat{M}_I(5,1)\right],\ \vec{\hat{M}}[\gamma_4(b1)]=\left[\hat{M}_D(4,5)\right],\ \vec{\hat{M}}[\gamma_6(b2)]=\left[\hat{M}_I(5,1)\right],\ \vec{\hat{M}}[\gamma_4(b4)]=\left[\hat{M}_D(4,4)\right]\right\},$$
$$\left\{\vec{\vec{\hat{M}}}(b)\right\}_T[C_8]=\left\{\vec{\hat{M}}[\gamma_{11}(b6)]=\left[\hat{M}_I(10,1)\right],\ \vec{\hat{M}}[\gamma_9(b1)]=\left[\hat{M}_D(11,11)\right]\right\}.$$

Figure S2. MSA regions potentially able to accommodate local indel histories along tree. **a** A global indel history along a tree. Sequence IDs are assigned to the nodes. Each branch is accompanied with an ID ($b1$ - $b6$) and its own gobal indel history. The " R " stands for the root. **b** Resulting MSA of the "extant" sequences at external nodes and the ancestral sequences at internal nodes. The boldface letters in the leftmost column are the node IDs.

Below the MSA, the bottom curly brackets indicate regions C_{K} ($\mathrm{K} = 4{,}8$ in this example) that actually accommodate local indel histories along the tree, And the yellow wedges indicate the regions C_{K} ($\mathrm{K} = 1{,}2{,}3{,}5{,}6{,}7{,}9{,}10$ in this example) that can potentially accommodate local indel histories along the tree, but that actually do not. In this example, $\mathrm{K}_{\mathrm{max}} = 10$. **c** LHSs along the branches (in the vector representation). As examples, the PWAs along branches $b1$ and $b5$ are also shown, along with their own potentially local-history-accommodating regions. **d** LHS along the tree (vector representation). Only the non-empty components were shown explicitly.

The figure follows basically the same notation as Figure S1 does. A cell in the MSA is shaded only if it is inserted/deleted along an adjacent branch. The figure was adapted from Figure 11 of [32].

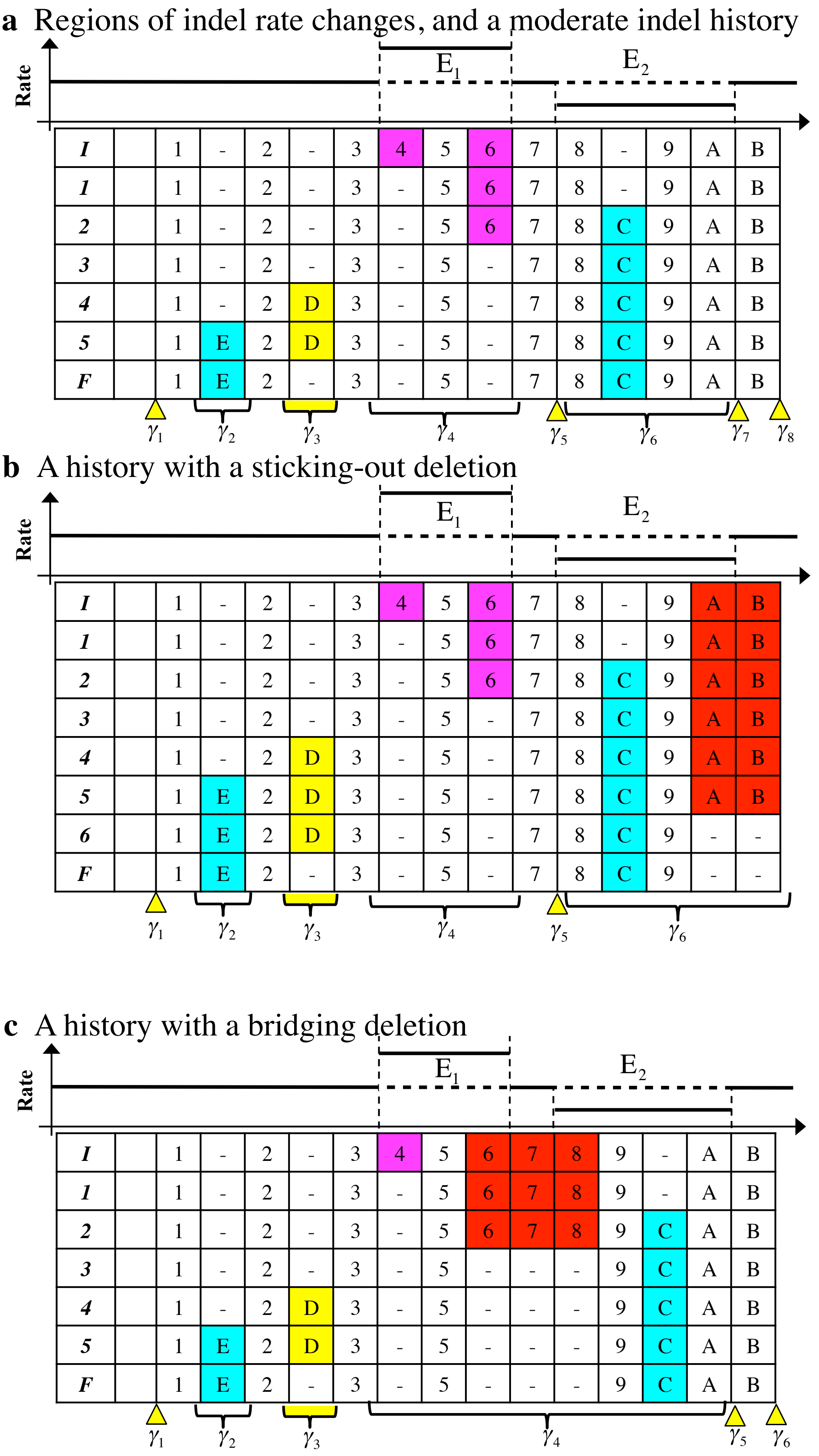

Figure S3. Example of the partially factorable indel model, Eqs.(R8-3.1,2).

a Regions confining indel rate changes. In this panel, all indels are either completely within or

outside of the regions. The graph above the MSA schematically indicates the indel rates of the regions. Indel rate changes are confined in two regions, E_1 and E_2. Other than that, the figure uses the same notation as in Figure S1. Although the deletion of a site with ancestry '4' and the deletion of a site with ancestry '6' are separated by a PAS (with ancestry '5'), they are lumped together to form a single local indel history, because they are both contained in E_1. **b** When a deletion sticks out of a region of changed indel rates. The deletion of the two sites (with ancestries 'A' and 'B') sticks out of region E_2. In this case, γ_6 is extended to encompass this deletion, and ends up engulfing the old γ_7 and γ_8. All indel events within this new γ_6 define a single local indel history. **c** When a deletion bridges two regions of changed indel rates. The deletion of the three sites (with ancestries '6,' '7' and '8') bridges regions E_1 and E_2. In this case, E_1 and E_2, as well as the spacer region between them, are put together to form a "meta-region" (the new γ_4). And the indel events within the meta-region are lumped together to form a single local indel history. The figure was adapted from Figure 12 of [32].

Supplementary table

Table S1. Mathematical symbols common in this paper

[NOTE: The symbols are arranged in the following order: Non-alphabetic symbols -> Roman alphabetic characters -> Greek alphabetic characters.]

Symbol	Description	First occurrence (or definition)
**Non-alphabetic symbols**		
$\langle x \vert$ (bra)	A bra-vector that represents the state x. (A bra-vector is an extension of a row-vector in the standard formulation.)	Background; Supplementary appendix SA-1
$\vert y \rangle$ (ket)	A ket-vector that "accepts" the state y. (A ket-vector is an extension of a column-vector in the standard formulation.)	Background; Supplementary appendix SA-1
$\hat{O}$ (hat)	An operator that represents the action of O. (An operator is an extension of a matrix in the standard formulation.)	Background; Supplementary appendix SA-1
$X \sim Y$ (tilde)	X is equivalent to Y.	In general
**Beginning with Roman alphabetic characters**		
$\{b\}_T$	The set of all branches of the tree (T).	Section R7, 2nd paragraph
$C_1, C_2, ..., C_{\mathrm{K_{max}}}$	The maximum possible set of regions each of which can accommodate local indel histories consistent with the portion of a given MSA confined in the region.	Section R7, above Eq.(R7.8)
$\tilde{\mathrm{H}}^{ID}(s_0)$	The set of all possible indel histories along a time axis (or a branch) that begin with the sequence state, s_0.	Section R7, above Eq.(R7.1)
$\mathrm{H}^{ID}(N; s_0)$	The set of all possible histories of N	Section R4,

	indels each along a time axis (or a branch) that begin with the sequence state, s_0 .	Eq.(R4.6)
$\tilde{\mathrm{H}}^{ID}\left[\alpha(s^A, s^D)\right]$	The set of all indel histories consistent with the PWA, $\alpha(s^A, s^D)$.	Section R4, above Eq.(R4.9)
$\mathrm{H}^{ID}\left[N; \alpha(s^A, s^D)\right]$	The set of all indel histories with N indels each that can result in the PWA, $\alpha(s^A, s^D)$.	Section R4, Eq.(R4.8)
$\hat{I}$	The identity operator.	Section R3, Eq.(R3.18)
$L(s)$	The length of a sequence in state s .	Section R3
$\hat{M}_D(x_B, x_E)$	The deletion of the subsequence between (and including) the x_B -th and x_E -th sites.	Section R2, Figure 3c
$\hat{M}_I(x, l)$	The insertion of l sites between the x -th and ($x+1$)-th sites.	Section R2, Figure 3b
$\hat{M}_\nu$	The ν -th event in an indel history.	Section R4, Eq.(R4.7)
$\vec{\hat{M}} = [\hat{M}_1, \hat{M}_2, \cdots, \hat{M}_N]$	An indel history consisting of N indel events, $\hat{M}_1, \hat{M}_2, \cdots, \hat{M}_N$.	Section R4, Eqs.(R4.6,7)
$\hat{M}_\nu(b)$	The ν th event in an indel history along the branch, b .	Section R7, Eq.(R7.1)
$\vec{\hat{M}}(b)$	An indel history along the branch, b .	Section R7, Eq.(R7.1)
$\left\{\vec{\hat{M}}(b)\right\}_T$	An indel history along the tree, T .	Section R7, Eq.(R7.1)
$\hat{M}[k, i_k]$	The operator representing the i_k -th event in the k -th local indel history isolated from a global indel history.	Section R5, Eq.(R5.4)
$\vec{\vec{\hat{M}}} = \left\{\left[\hat{M}[k,1], ..., \hat{M}[k, N_k]\right]\right\}_{k=1,...,K}$	A local history set (LHS) that consists of K local indel histories, which in isolation are: $\left[\hat{M}[k,1], ..., \hat{M}[k, N_k]\right]$ with $k = 1,...,K$.	Section R5 (2nd-last paragraph); Section R6, Eq.(R6.1)
$\vec{\hat{M}}[\gamma_\kappa]$	A local indel history that can yield the portion of a given PWA confined in the	Section R6, Eq.(R6.7)

	region, γ_κ.	
$\vec{\vec{\hat{M}}} = \left(\vec{\hat{M}}[\gamma_1], \vec{\hat{M}}[\gamma_2], ..., \vec{\hat{M}}[\gamma_{\kappa_{\max}}]\right)$	The vector representation of the LHS ($\vec{\vec{\hat{M}}}$), using the set of finest local regions, $\gamma_1, \gamma_2, ..., \gamma_{\kappa_{\max}}$.	Section R6, above Eq.(R6.7)
$\left[\vec{\vec{\hat{M}}}\right]_{LHS}$	A local-history-set (LHS) equivalence class represented by the LHS, $\vec{\vec{\hat{M}}}$ (*e.g.*, $= \left\{\left[\hat{M}[k,1], ..., \hat{M}[k, N_k]\right]\right\}_{k=1,...,K}$).	Section R6, Eq.(R6.1)
$\mathrm{N}_1 \left(= \{1, 2, 3, ...\}\right)$	The set of all positive integers.	In general
$N_{\min}\left[\alpha(s^A, s^D)\right]$	The minimum number of indels required for creating the PWA, $\alpha(s^A, s^D)$.	Section R4, Eq.(R4.8)
$\mathrm{N}^{IN}(T)$	The set of all internal nodes of the tree (T).	Section R7, 2nd paragraph
$N^X \left(\equiv \left\|\mathrm{N}^X(T)\right\|\right)$	The number of external nodes of the tree (T).	Section R7, 2nd paragraph
$\mathrm{N}^X(T)\left(= \{n_1, ..., n_{N^X}\}\right)$	The set of all external nodes of the tree (T).	Section R7, 2nd paragraph
$\{n\}_T \left(= \mathrm{N}^{IN}(T) + \mathrm{N}^X(T)\right)$	The set of all nodes of the tree (T).	Section R7, 2nd paragraph
$n^A(b)$	The “ancestral node” on the upstream end of the branch (b).	Section R7, 2nd paragraph
$n^D(b)$	The “descendant node” on the downstream end of the branch (b).	Section R7, 2nd paragraph
n^{Root}	The root node of a given tree.	Section R7, 2nd paragraph
$P\left[(s, n)\right]$	The probability that the sequence is in state s at node n of the tree.	Section R7, Eq.(R7.4)
$P\left[X \mid Y\right]$	The conditional probability that we have the outcome (X) conditioned on Y .	In general
$P\left[(s', t') \mid (s, t)\right]$	The conditional probability that the sequence is in state s' at time t' conditioned on	Section R3, Eq.(R3.17)

	that it was in state s at time t.	
$P\left[([\,],[t_I,t_F])\,\vert\,(s_0,t_I)\right]$ $\left(=\exp\left\{-\int_{t_I}^{t_F} d\tau\, R_X^{ID}(s_0,\tau)\right\}\right)$	The probability that the sequence with an initial state, s_0, underwent no indel during the time interval, $[t_I,t_F]$.	Section R4, below Eq.(R4.7)
$P_0\left[s_0^{Root}\,\vert\,T\right]$	The probability that the sequence was in state s_0^{Root} at the root and that it underwent no indels all across the tree (T).	Section R7, Eq.(R7.10)
$\hat{P}^{ID}(t,t')$	The finite-time transition operator of our indel evolutionary model, from time t to time t'.	Section R3, Eq.(R3.17)
$\hat{P}_0^{ID}(t',t'')$	$\equiv T\left\{\exp\left(\int_{t'}^{t''} d\tau\, \hat{Q}_0^{ID}(\tau)\right)\right\}$, *i.e.*, the operator describing the evolution from t' till t'' with no indel.	Section R4, Eq.(R4.4), below Eq.(SM-1.4)
$\hat{Q}^{ID}(t)\left(=\hat{Q}^I(t)+\hat{Q}^D(t)\right)$	The total rate operator (at time t) of our indel evolutionary model.	Section R3, Eq.(R3.11)
$\hat{Q}_0^{ID}(t)\left(\equiv\hat{Q}_X^I(t)+\hat{Q}_X^D(t)\right)$	The mutation-free part of the total rate operator ($\hat{Q}^{ID}(t)$).	Section R4, Eq.(R4.1), Eq.(R4.2)
$\hat{Q}_M^{ID}(t)\left(\equiv\hat{Q}_M^I(t)+\hat{Q}_M^D(t)\right)$	The part of the total rate operator ($\hat{Q}^{ID}(t)$) describing the single-mutation transition between states.	Section R4, Eq.(R4.1)
$\hat{Q}^m(t)=\left(\hat{Q}_M^m(t)+\hat{Q}_X^m(t)\right)$	The component of the rate operator (at time t) due to mutations of type $m\,(=I \text{ or } D)$.	Section R3, Eq.(R3.2)
$\hat{Q}_M^m(t)$	The "mutation part" of the rate operator that describes the instantaneous transition (at time t) via mutations of type $m\,(=I \text{ or } D)$.	Section R3, Eq.(R3.2), Eqs.(R3.12, 13)
$\hat{Q}_X^m(t)$	The "exit rate part" of the rate operator that attenuates the state retention probability via mutations of type $m\,(=I \text{ or } D)$.	Section R3, Eq.(R3.2), Eq.(R3.6)
$R_X^{ID}(s,t)\equiv R_X^I(s,t)+R_X^D(s,t)$	The total exit rate of the sequence state (s) at time t due to indels.	Section R4, Eq.(R4.3)
$R_X^m(s,t)$	The component of the exit rate of the	Section R3,

	sequence state (s) at time t due to mutations of type $m\left(= I \text{ or } D\right)$.	Eqs.(R3.14, 15)
$r(\hat{M}; s, t)$	The rate of the mutation represented by $\hat{M}$ on the sequence in state s at time t. (In general, the rate depends on s and t.)	Section R4, Eq.(R4.7); Eq.(SM-1.13)
$r_D\left(x_B, x_E; s, t\right)$	The rate of deletion of the subsequence between (and including) the x_B -th and x_E -th sites, from the sequence (in state s) at time t. (The rate generally depends on s and t.)	Section R3 (near the top)
$r_I\left(x, l; s, t\right)$	The rate of insertion of l sites between the x -th and ($x+1$)-th sites of the sequence (in state s) at time t. (The rate generally depends on s and t.)	Section R3 (near the top), Eq.(R3.16)
$S^{II}\left(\subset \Upsilon^* = \bigcup_{L=0}^{\infty} \Upsilon^L\right)$	The space of all basic sequence states.	Section R2
$s\left(= \vec{\upsilon} = \left[\upsilon_1, \upsilon_2, ..., \upsilon_L\right]\right)$	A basic sequence state (of length L), in which each site (x) is assigned an ancestry (υ_x) alone.	Section R2, Figure 2c
$\breve{s} = \left[(\upsilon_1, \omega_1), (\upsilon_2, \omega_2), ..., (\upsilon_L, \omega_L)\right]$	An extended sequence state (of length L), in which each site (x) is assigned an ancestry (υ_x) and a residue (ω_x).	Section R2, Figure 2b
$s(n)\left(\in S^{II}\right)$	The sequence state at the node $n \in \{n\}_T$.	Section R7, 2nd paragraph
$s^A(b)\left(\equiv s\left(n^A(b)\right)\right)$	The sequence state at the "ancestral node" on the upstream end of branch b.	Section R7, 2nd paragraph
$s^D(b)\left(\equiv s\left(n^D(b)\right)\right)$	The sequence state at the "descendant node" on the downstream end of branch b.	Section R7, 2nd paragraph
$s^{Root} = s(n^{Root})$	The sequence state at the root node.	Section R7, 3rd paragraph
s_0^{Root}	A "reference" root state.	Section R7, above Eq.(R7.8)
$\left\{s(n)\right\}_{\mathrm{N}^{IN}}$	A set of ancestral states at all internal nodes.	Section R7,

		above Eq.(R7.5)
$T\left(=\left(\{n\}_T, \{b\}_T\right)\right)$	A (rooted) phylogenetic tree.	Section R7, 2nd paragraph
$T\{...\}$	The (summation of) time-ordered product(s). It rearranges the operators in each product in the temporal order so that the earliest operator comes leftmost.	Section R3, Eq.(R3.18); Eq.(SA-1.11)
$\bigcup_{a \in A} X(a)$	The union of the sets (spaces), $X(a)$'s, which form a function on a space (set), A, over all elements (a's) in A.	In general
**Beginning with Greek alphabetic characters**		
$\alpha(s^A, s^D)$	A PWA between the ancestral sequence (s^A) and the descendant sequence (s^D).	Section R4, above Eq.(R4.8)
$\alpha[s_1, s_2, ..., s_{N^X}]$	A MSA among the sequence at the external nodes, $s_i = s(n_i) \in S^{II}$ ($n_i \in \mathrm{N}^X(T)$).	Section R7, above Eq.(R7.4)
$\gamma_1, \gamma_2, ..., \gamma_{\kappa_{\max}}$	The finest regions each of which can potentially accommodate local indel histories consistent with a given PWA.	Section R6, above Eq.(R6.7)
$\delta R_X^{ID}(s, s', t) \equiv R_X^{ID}(s, t) - R_X^{ID}(s', t)$	The difference of the exit rate of state s from that of state s' at time t.	Section R6, condition (ii); Eq.(SM-2.7)
$\Theta_{ID}(b)$	The model parameters for the indel processes along the branch, b.	Section R7, 2nd paragraph
$\mathrm{K}_{\max}$	The maximum possible number of the potentially local-history-accommodating regions consistent with a given MSA.	Section R7, above Eq.(R7.8)
$\kappa_{\max}$	The number of the finest potentially local-history-accommodating regions consistent with a given PWA.	Section R6, above Eq.(R6.7)

$\tilde{\Lambda}^{ID}\left[\alpha(s^A, s^D)\right]$	The set of all local history sets (LHSs) consistent with a PWA ($\alpha(s^A, s^D)$).	Section R6, Eq.(R6.5)
$\tilde{\Lambda}^{ID}\left[\gamma_K; \alpha(s^A, s^D)\right]$	The set of local indel histories that can give rise to the sub-PWA of $\alpha(s^A, s^D)$ confined in γ_K .	Section R6, Eq.(R6.7)
$\tilde{\tilde{\mathrm{M}}}_P\left[\begin{matrix}\alpha[s_1, s_2, ..., s_{N^X}]; \\ s_0^{Root}; C_K \quad \vert T\end{matrix}\right]$	The multiplication factor contributed from all local indel histories along the tree (T) each of which can yield the portion of a MSA ($\alpha[s_1, s_2, ..., s_{N^X}]$) confined in the region, C_K .	Section R7, Eq.(R7.9), below Eq.(R7.10)
$\mu_P\left[s^{Root}, s_0^{Root}, n^{Root}; C_K\right]$	The (multiplicative) change in the state probability at the root (n^{Root}) due to the difference between the states, s^{Root} and s_0^{Root} , within the region, C_K .	Section R7, Eq.(R7.8)
$\mu_P\left[\left(\left[\begin{matrix}\hat{M}[k,1], \\ ..., \\ \hat{M}[k,N_k]\end{matrix}\right], [t_I, t_F]\right) \middle\vert (s^A, t_I)\right]$	The probability quotient (multiplication factor) from the local indel history, $\left[\hat{M}[k,1], ..., \hat{M}[k,N_k]\right]$.	Section R6, Eq.(R6.2), Eq.(R6.3)
$\mu_P\left[\left(\left[\hat{\vec{\vec{M}}}\right]_{LHS}, [t_I, t_F]\right) \middle\vert (s^A, t_I)\right]$	The total probability quotient (multiplication factor) from the LHS equivalence class, $\left[\hat{\vec{\vec{M}}}\right]_{LHS}$.	Section R6, Eq.(R6.2), Eq.(R6.4)
$\prod_{a \in A} F(a)$	The product of the values of a function, $F(a)$, over all elements (a 's) in the space (set), A .	In general
$\sum_{a \in A} F(a)$	The summation of the values of a function, $F(a)$, over all elements (a 's) in the space (set) A .	In general
$\Sigma\left[\begin{matrix}\alpha[s_1, s_2, ..., s_{N^X}]; \\ \left\{n \in \mathrm{N}^{IN}(T)\right\}; T\end{matrix}\right]$	The set of all $\left\{s(n)\right\}_{\mathrm{N}^{IN}}$'s (*i.e.*, all sets of sequence states at internal nodes) that are consistent with the MSA, $\alpha[s_1, s_2, ..., s_{N^X}]$, and the tree, T .	Section R7, above Eq.(R7.5); above Eq.(SM-4.5)

Υ	The set of ancestry indices.	Section R2
$\upsilon_x \ (\in \Upsilon)$	The ancestry index assigned to the x-th site of a sequence.	Section R2
$\vec{\upsilon} = [\upsilon_1, \upsilon_2, ..., \upsilon_L]$	An array of ancestry indices assigned to the sites of a sequence (of length L).	Section R2, Figure 2c
$\tilde{\Psi}^{ID}[\alpha[s_1, s_2, ..., s_{N^X}]; T]$	The set of all pairs, $\left(s^{Root}, \left\{\hat{\vec{M}}(b)\right\}_T\right)$, defined on T that are consistent with the MSA, $\alpha[s_1, s_2, ..., s_{N^X}]$.	Section R7, above Eq.(R7.4); above Eq.(SM-4.4)
Ω	An alphabet, or the set of all possible residues (such as 4 bases for DNA or 20 amino acids for proteins).	Section R1
$\omega_x \ (\in \Omega)$	The residue at the x-th site of a sequence.	Section R1
$\vec{\omega} = [\omega_1, \omega_2, ..., \omega_L]$	An array of residues assigned to the sites of a sequence (of length L).	Section R1, Figure 2a
