# Supplementary material for: General continuous-time Markov model of sequence evolution via insertions/deletions: are alignment probabilities factorable?
Source: BMC Bioinformatics. 2016 Aug 11;17:304. doi: 10.1186/s12859-016-1105-7 (PMC5026781; doi:10.1186/s12859-016-1105-7)
Supplement: Additional file 2: — A PDF file that consists of Supplementary appendix (sections SA-1, SA-2 and SA-3). The sections of Supplementary appendix provide mathematical derivations of some important results. (PDF 9581 kb) [file 12859_2016_1105_MOESM2_ESM.pdf]

Additional file 2 of
"General continuous-time Markov model of sequence evolution via insertions/deletions: Are alignment probabilities factorable?"

by Kiyoshi Ezawa

Table of contents

Supplementary appendix **pp. 2-27**

SA-1. Equivalence of bra-ket formulation to vector-matrix formulation pp. 2-6

SA-2. Factorability of pairwise alignment probability: mathematically rigorous proof pp. 6-21

SA-2.1. Outline pp. 7-12

SA-2.2. Proof of factorization of multiple-time integration, Eq.(SA-2.1.3) pp. 12-17

SA-2.3. Proof of proposition SA-2.1.1 for factorization of exponential factor pp. 17-21

SA-3. Total probability of LHS equivalence class under "long indel" model pp. 21-27

Supplementary appendix

This part of the paper may not necessarily be essential for understanding the main points of the paper. Nevertheless, it may be crucial for those readers who intend to improve, extend or adapt our theoretical formulation to whatever suits their needs. It may also entertain those readers who are fond of mathematics. The sections below were all adapted from the relevant (sub-)sections of our unpublished paper deposited in bioRxiv [32].

SA-1. Equivalence of our bra-ket formulation to standard vector-matrix formulation of continuous-time Markov model

[This section was adapted from subsection 1.1 of [32]. See subsection 1.2 of *ibid.* for a simple example. And see subsection 1.3 of *ibid.* for a brief discussion on the differences from the use of bra-ket notation in quantum mechanics.]

Let us first recall the conventional formulation of a general continuous-time Markov model on a finite space consisting of N states, $i = 1, 2, \ldots, N$. One way of formulating the model is to specify a rate matrix, $Q = (q_{ij})$. Let q_{ij} denote the (i, j)-element of Q, *i.e.*, its element at the intersection of the i th row and the j th column. Then, the non-diagonal element q_{ij} ($i \neq j$) of a rate matrix Q is the rate (per certain unit time) at which the system moves to the j th state, given it was in the i th state immediately before the time in question. The diagonal element, q_{ii}, is usually given by the equation:

$$q_{ii} = -\sum_{\substack{j=1 \\ (j \neq i)}}^{N} q_{ij} \ , \qquad \text{--- Eq.(SA-1.1)}$$

to guarantee that the summation of the probabilities over the states remain 1 (unity) all the time. Now, let the probability vector, $\vec{p}(t) = \left(p_i(t)\right)$, be a row vector whose i th element, $p_i(t)$, is the probability that the system is in the i th state at time t. Then, under the above Markov model, $\vec{p}(t)$ satisfies the 1st order time differential equation:

$$\frac{d}{dt}\vec{p}(t) = \vec{p}(t)\, Q \qquad \left(\text{or} \quad \frac{d}{dt}p_i(t) = \sum_{j=1}^{N} p_j(t) q_{ji} \right). \text{--- Eq.(SA-1.2)}$$

The general solution of this equation at a finite time $t (> 0)$ is given by:

$$\vec{p}(t) = \vec{p}(0)\, P(t) \qquad \left(\text{or} \quad p_i(t) = \sum_{j=1}^{N} p_j(0)\, p_{ji}(t)\right). \qquad \text{--- Eq.(SA-1.3a)}$$

Here the finite-time transition matrix, $P(t) = \left(p_{ij}(t)\right) = \exp\left(tQ\right)$, is an $N \times N$ matrix whose (i, j)-element $p_{ij}(t)$ is the probability that the system is in the j th state at time t, conditioned on that it was in the i th state initially (*i.e.*, at time $t = 0$):

$$p_{ij}(t) = \left[\exp(tQ)\right]_{ij} = P\left[(j,t)\,\middle|(i,0)\right] \ . \qquad \text{--- Eq.(SA-1.3b)}$$

If Eq.(SA-1.1) holds, the matrix elements satisfy $\sum_{j=1}^{N} p_{ij}(t) = 1$ (unity) for all $i = 1, 2, \ldots, N$. Meanwhile, $\vec{p}(0) = \left(p_i(0)\right)$ is the initial probability vector, whose i th component, $p_i(0)$, is the probability that the system was in the i th state at time $t = 0$. They satisfy $\sum_{j=1}^{N} p_j(0) = 1$. This could be made more explicit by using the basic row vectors, $\{\vec{e}_i\}_{i=1,2,\ldots,N}$. Here, $\vec{e}_i \equiv (0,\ldots,1,\ldots,0)$ is the row vector with all zero except the i th component, which is 1 (unity). This "basic vector" represents the situation where the system is in the i th state. Using these basic vectors, the initial probability vector is expressed as:

$$\vec{p}(0) = \sum_{i=1}^{N} p_i(0)\vec{e}_i \ . \qquad \text{--- Eq.(SA-1.4)}$$

The expression is interpreted as the initial condition that the system is in the i th state with probability $p_i(0)$ ($i = 1, 2, \ldots, N$). Similarly, the probability vector at any time could be expressed as:

$$\vec{p}(t) = \sum_{i=1}^{N} p_i(t)\vec{e}_i \ . \qquad \text{--- Eq.(SA-1.5)}$$

It is interpreted as the situation where the system is in the i th state with probability $p_i(t)$ ($i = 1, 2, \ldots, N$) given by Eq.(SA-1.3a). Using the basic vectors, the conditional probabilities can be formally extracted from the finite-time transition matrix, $P(t) = \exp\left(tQ\right)$, by a matrix multiplication:

$$P\left[(j,t)\,\middle|(i,0)\right] = p_{ij}(t) = \vec{e}_i\, P(t)(\vec{e}_j)^t \ . \qquad \text{--- Eq.(SA-1.6)}$$

Here, $(\vec{e}_i)^t$ is the column vector obtained from the row vector, $\vec{e}_i$, by a matrix transposition operation (*i.e.*, by interchanging the rows with the columns).

Now we can introduce the bra-ket notation and operators. First, we replace each basic row vector, $\vec{e}_i$, with the corresponding basic bra-vector, $\langle i|$, and replace each basic column vector, $(\vec{e}_j)^t$, with the corresponding basic ket-vector, $|j\rangle$. Then, the bra-vector corresponding to the probability vector $\vec{p}(t) = \left(p_i(t)\right)$ in Eq.(SA-1.5) is given by the following linear combination of the basic bra-vectors:

$$\langle \vec{p}(t)| = \sum_{i=1}^{N} p_i(t)\langle i| \ . \qquad \text{--- Eq.(SA-1.5')}$$

In the present formulation, the exclusive role of a ket-vector is that it serves as an "acceptor" of bra-vectors. More specifically, we will make the ket-vector, $|j\rangle$, accept only the

corresponding bra-vector, $\langle j|$, by defining the scalar products:

$$\langle i|j\rangle = 1 \; if \; i = j, = 0 \; if \; i \neq j \; . \qquad \text{--- Eq.(SA-1.7)}$$

Using these scalar products, we get, *e.g.*, the equation, $\langle \vec{p}(t)|i\rangle = p_i(t)$, from Eq.(SA-1.5').
Next, we introduce (linear) operators that transform each bra-vector into a specified linear combination of bra-vectors. The operators are analogs of matrices in the traditional formulation. For example, we could define an operator, $\hat{m}(i \to j)$, that transforms (or "mutates") the i th state to the j th state, but does nothing else:

$$\begin{aligned} &\langle i|\hat{m}(i \to j) = \langle j|, \\ &\langle k|\hat{m}(i \to j) = 0 \quad for \; k \neq i \, . \end{aligned} \qquad \text{--- Eq.(SA-1.8)}$$

This operator corresponds to the matrix whose elements are all zero except the (i,j)–element, which is 1 (unity). Now, we define the (instantaneous transition) rate operator, $\hat{Q}$, as follows:

$$\langle i|\hat{Q} \;\; \equiv \;\; \sum\nolimits_{j=1}^{N} q_{ij} \langle j| \;\; . \qquad \text{--- Eq.(SA-1.9)}$$

Then, we get the following equation:

$$\langle \vec{p}(t)|\hat{Q} = \sum\nolimits_{j=1}^{N} p_j(t)\langle j|\hat{Q} = \sum\nolimits_{j=1}^{N} p_j(t)\left\{\sum\nolimits_{i=1}^{N} q_{ji}\langle i|\right\} = \sum\nolimits_{i=1}^{N}\left\{\sum\nolimits_{j=1}^{N} p_j(t) q_{ji}\right\}\langle i| \;\; .$$

Then, substituting Eq.(SA-1.2) for the expression in braces on the leftmost hand side, we have:

$$\langle \vec{p}(t)|\hat{Q} = \sum\nolimits_{i=1}^{N} \frac{d}{dt} p_i(t)\langle i| = \frac{d}{dt}\left\{\sum\nolimits_{i=1}^{N} p_i(t)\langle i|\right\} \;\; .$$

This means that we can recast the defining equation, Eq.(SA-1.2), of the continuous-time Markov model into the equation satisfied by the probability bra-vector $\langle \vec{p}(t)|$:

$$\frac{d}{dt}\langle \vec{p}(t)| = \langle \vec{p}(t)|\hat{Q} \quad . \qquad \text{--- Eq.(SA-1.2')}$$

This equation can be integrated as:

$$\langle \vec{p}(t)| = \langle \vec{p}(0)|\hat{P}(t) \quad , \qquad \text{--- Eq.(SA-1.3a')}$$

with the finite-time transition operator, $\hat{P}(t) \equiv \exp(t\hat{Q})$. And the counterpart of Eq.(SA-1.3b) is:

$$\langle i|\hat{P}(t)|j\rangle = \langle i|\exp(t\hat{Q})|j\rangle = P\left[(j,t)\,|(i,0)\right] \;\; . \qquad \text{--- Eq.(SA-1.3b')}$$

Solving Eq.(SA-1.2') for every possible initial probability bra-vector, $\langle \vec{p}(0)| = \sum\nolimits_{i=0}^{N} p_i(0)\langle i|$, is equivalent to solving the following equation for the operator $\hat{P}(t)$:

$$\frac{d}{dt}\hat{P}(t) = \hat{P}(t)\,\hat{Q} \quad , \qquad \text{--- Eq.(SA-1.10a)}$$

with the initial condition,

$$\hat{P}(0) = \hat{I} \, , \qquad \text{--- Eq.(SA-1.10b)}$$

where $\hat{I}$ is the identify operator: $\langle i|\hat{I} = \langle i|$ for every state i. Thus, if desired, Eqs.(SA-1.10a,b) could be considered as the defining equation of the continuous-time Markov model.

Thus far, we tacitly assumed that the Markov model is time-homogeneous, where the rate matrix Q, or the rate operator $\hat{Q}$, is independent of time t. In reality, the transition rate, q_{ij}, could depend on time due to, *e.g*., the temporal change of the environment the system is in. Here, we extend the formulation developed above to the system with a *time-dependent* rate matrix, $Q(t) = \left(q_{ij}(t)\right)$, whose operator counterpart is denoted as $\hat{Q}(t)$.

Because the model is no longer homogeneous in time, when we consider a finite-time evolution of the system, we need to specify the initial time t_I, in addition to the final time $t_F\ (> t_I)$. Let $\hat{P}(t_I, t_F)$ be the operator describing the finite-time transition during the closed time interval, $[t_I, t_F]$, that is:

$$\langle i|\hat{P}(t_I, t_F)|j\rangle = P\left[(j, t_F)\,|\,(i, t_I)\right] \quad for \quad {}^\forall i, j \in \{1, 2, ..., N\}, \quad t_F > t_I \, ,$$

under a *time-heterogeneous* continuous-time Markov model with the rate operator $\hat{Q}(t)$. Then, the defining equations, Eqs.(SA-1.10a,b), are extended to fit this model as:

$$\frac{d}{dt}\hat{P}(t_I, t) = \hat{P}(t_I, t)\,\hat{Q}(t) \quad , \qquad \text{--- Eq.(SA-1.10a')}$$

$$\hat{P}(t, t) = \hat{I} \quad for \quad {}^\forall t \quad . \qquad \text{--- Eq.(SA-1.10b')}$$

The general solution of the above equations is symbolically given by:

$$\hat{P}(t_I, t) = T\left\{\exp\left(\int_{t_I}^{t} dt'\,\hat{Q}(t')\right)\right\} \quad . \qquad \text{--- Eq.(SA-1.11)}$$

Here $T\{...\}$ denotes (the summation of) the time-ordered product(s), which arrange(s) multiplied operators in the temporal order so that the earliest operator will come leftmost. For example,

$$T\left\{\hat{A}(t_1)\,\hat{B}(t_2)\right\} \equiv \begin{cases} \hat{A}(t_1)\,\hat{B}(t_2) & for \quad t_1 < t_2 \, , \\ \hat{B}(t_2)\,\hat{A}(t_1) & for \quad t_2 < t_1 \, . \end{cases}$$

We could regard the time-ordered exponential in Eq.(SA-1.11) as defined by a limit:

$$T\left\{\exp\left(\int_{t_I}^{t} dt'\,\hat{Q}(t')\right)\right\} \equiv \lim_{L\to\infty}\left(\hat{I}+\tfrac{t-t_I}{L}\hat{Q}(t_1^{(L)})\right)\left(\hat{I}+\tfrac{t-t_I}{L}\hat{Q}(t_2^{(L)})\right)\cdots\left(\hat{I}+\tfrac{t-t_I}{L}\hat{Q}(t_L^{(L)})\right) ,$$

where $t_k^{(L)} \equiv t_I + (i-\frac{1}{2})\frac{t-t_I}{L}$, or as defined by a series:

$$T\left\{\exp\left(\int_{t_I}^{t} d\tau\,\hat{Q}(\tau)\right)\right\} \equiv \hat{I}+\sum_{n=1}^{\infty}\int d\tau_1\cdots\int_{t_I<\tau_1<\ldots<\tau_n<t} d\tau_n\,\hat{Q}(\tau_1)\cdots\hat{Q}(\tau_n)$$

$$= \hat{I}+\int_{t_I}^{t} d\tau_1\,\hat{Q}(\tau_1)+\int_{t_I}^{t} d\tau_1\int_{\tau_1}^{t} d\tau_2\,\hat{Q}(\tau_1)\hat{Q}(\tau_2)+\int_{t_I}^{t} d\tau_1\int_{\tau_1}^{t} d\tau_2\int_{\tau_2}^{t} d\tau_3\,\hat{Q}(\tau_1)\hat{Q}(\tau_2)\hat{Q}(\tau_3)+\ldots$$

Moreover, the finite-time transition operator given by Eq.(SA-1.11) also satisfies the "backward equation":

$$\frac{d}{dt}\hat{P}(t,t_F) = -\hat{Q}(t)\,\hat{P}(t,t_F) \quad , \qquad \text{--- Eq.(SA-1.12)}$$

as well as the Chapman-Kolmogorov equation (aka the multiplicativity condition):

$$\hat{P}(t_I,t_F) = \hat{P}(t_I,t_M)\,\hat{P}(t_M,t_F) \quad (t_I < t_M < t_F) \quad ., \qquad \text{--- Eq.(SA-1.13)}$$

The latter could be rewritten in terms of conditional probabilities:

$$P\left[(j,t_F)\,|(i,t_I)\right] = \sum_{k=1}^{N} P\left[(k,t_M)\,|(i,t_I)\right] P\left[(j,t_F)\,|(k,t_M)\right]. \qquad \text{--- Eq.(SA-1.13')}$$

The last equation can be obtained by sandwiching the both sides of Eq.(SA-1.13) with $\langle i|$ and $|j\rangle$, and by inserting the decomposition of the identity operator, $\hat{I} = \sum_{k=1}^{N}|k\rangle\langle k|$, between the two finite-time transition operators on its right-hand side.

As described above, we have reformulated a continuous-time Markov model on a finite set of states in terms of bra-vectors, ket-vectors and operators. Once we formulated it this way, we could extend the formulation to continuous-time Markov models defined on any discrete set of states, irrespective of whether it is finite, countably infinite, or uncountable, as long as the state space and the elementary transitions within it are well-defined. This notation facilitated the development of our *ab initio* theoretical formulation of the general continuous-time Markov model describing the evolution of an entire sequence along a time-axis via insertions/deletions (and substitutions if desired).

SA-2. Factorability of pairwise alignment probability: mathematically rigorous proof

This section presents probably the mathematically toughest among the proofs/derivations given in this paper. Nevertheless, if you keep in mind the intuitive pictures acquired in the main text (and in Additional file 1), they may not be so formidable as they appear at first glance.

SA-2.1. Outline

[This subsection was adapted from a part of subsection 4.1 of [32].]

Here, we give a mathematically rigorous proof of the factorability of the probability of a given pairwise alignment (PWA) under the conditions (i) and (ii) given in section R6 of the main Results and discussion (and also in SM-2 in Additional file 1). Actually, in SM-2 in Additional file 1, we already gave a proof that is *nearly rigorous*; the only loose point there was the proof of the ansatz:

$$\mu_P\left[\left(\left[\vec{\vec{\hat{M}}}\right]_{LHS}, [t_I, t_F]\right) \middle| (s^A, t_I)\right] = \prod_{k=1}^{K} \mu_P\left[\left(\left[\hat{M}[k,1], \ldots, \hat{M}[k,N_k]\right], [t_I, t_F]\right) \middle| (s^A, t_I)\right],$$

-- Eq.(SM-2.8)

among the probability quotients, Eqs.(SM-2.4-6) (see also Eq.(SM-2.7)). Thus, we here focus on rigorously proving Eq.(SM-2.8). As in SM-2, we consider a local history set (LHS) denoted as: $\vec{\vec{\hat{M}}} = \left\{\left[\hat{M}[k,1], \ldots, \hat{M}[k,N_k]\right]\right\}_{k=1,\ldots,K}$.

First we recall that the left-hand side of Eq.(SM-2.8) is rewritten as:

$$\mu_P\left[\left(\left[\vec{\vec{\hat{M}}}\right]_{LHS}, [t_I, t_F]\right) \middle| (s^A, t_I)\right] = \sum_{[\hat{M}_1, \hat{M}_2, \cdots, \hat{M}_N] \in \left[\vec{\vec{\hat{M}}}\right]_{LHS}} \mu_P\left[\left([\hat{M}_1, \hat{M}_2, \cdots, \hat{M}_N], [t_I, t_F]\right) \middle| (s^A, t_I)\right]$$

$$= \sum_{[\hat{M}_1, \hat{M}_2, \cdots, \hat{M}_N] \in \left[\vec{\vec{\hat{M}}}\right]_{LHS}} \int\cdots\int_{t_I = \tau_0 < \tau_1 < \cdots < \tau_N < \tau_{N+1} = t_F} d\tau_1 \cdots d\tau_N \left[\begin{array}{l} \left(\prod_{\nu=1}^{N} r(\hat{M}_\nu; s_{\nu-1}, \tau_\nu)\right) \\ \times \exp\left\{-\sum_{\nu=0}^{N} \int_{\tau_\nu}^{\tau_{\nu+1}} d\tau\, \delta R_X^{ID}(s_\nu, s^A, \tau)\right\} \end{array} \right]_{s_0 = s^A,\ \left\{\begin{array}{l}\langle s_\nu| = \langle s_{\nu-1}|\hat{M}_\nu \\ for\ \nu = 1,\ldots,N\end{array}\right\}}.$$

--- Eq.(SA-2.1.1)

(It corresponds to Eq.(SM-2.9).) Here, $\delta R_X^{ID}(s, s', t) \equiv R_X^{ID}(s,t) - R_X^{ID}(s',t)$ denotes an increment of the exit rate. We also recall that the right-hand side is rewritten as:

$$\prod_{k=1}^{K} \mu_P\left[\left(\left[\hat{M}[k,1], \ldots, \hat{M}[k,N_k]\right], [t_I, t_F]\right) \middle| (s^A, t_I)\right]$$

$$= \prod_{k=1}^{K} \left[\begin{array}{l} \int\cdots\int_{t_I = \tau(k,0) < \tau(k,1) < \cdots < \tau(k,N_k) < \tau(k,N_k+1) = t_F} d\tau(k,1) \cdots d\tau(k,N_k) \left(\prod_{i_k=1}^{N_k} r\left(\hat{M}[k,i_k]; s_{i_k-1}, \tau(k,i_k)\right)\right) \\ \times \exp\left\{-\sum_{i_k=0}^{N_k} \int_{\tau(k,i_k)}^{\tau(k,i_k+1)} d\tau\, \delta R_X^{ID}(s_{i_k}, s^A, \tau)\right\} \Bigg|_{\langle s_0| = \langle s^A|,\ \left\{\begin{array}{l}\langle s_{i_k}| = \langle s_{i_k-1}|\hat{M}[k,i_k] \\ for\ i_k = 1,\ldots,N_k\end{array}\right\}} \end{array} \right].$$

--- Eq.(SA-2.1.2)

(It corresponds to Eq.(SM-2.10).) Here, $\tau(k, i_k)$ denotes the time at which the event $\hat{M}[k, i_k]$ virtually occurred in the isolated k th local history. Second, we note that the

subject LHS equivalence class, $\left[\hat{\bar{\bar{M}}}\right]_{LHS}$, consists of $\dfrac{N!}{\prod_{k=1}^{K} N_k!}$ global indel histories. Each history corresponds to a map from each event in each local indel history (specified by k) to a temporal order within the global history:

$$\pi: \ (k, i_k) \ (k = 1, ..., K; i_k = 1, ..., N_k) \mapsto \nu \, (= 1, ..., N).$$

The map keeps the relative temporal order among indels in each local indel history. Then, $[\hat{M}_1, \hat{M}_2, \cdots, \hat{M}_N]$ in the middle and on the rightmost side of Eq.(SA-2.1.1) corresponding to the above π can be more precisely written as: $\left[\hat{M}'[\pi^{-1}(1)], \hat{M}'[\pi^{-1}(2)], \cdots, \hat{M}'[\pi^{-1}(N)]\right]$. Here $\hat{M}'[\pi^{-1}(\nu)]$ is an equivalent of $\hat{M}[\pi^{-1}(\nu)]$ $(= \hat{M}[k, i_k]$ for $^{\exists}(k, i_k))$ through a series of binary equivalence relations, Eqs.(R5.2a-d), needed for rearranging the events in $\hat{\bar{\bar{M}}}$ this way. Now, let $\Pi\left(\left[\hat{\bar{\bar{M}}}\right]_{LHS}\right)$ be the set of such $\dfrac{N!}{\prod_{k=1}^{K} N_k!}$ maps. Then, the middle and the rightmost side of Eq.(SA-2.1.1) become:

$$\sum_{\pi \in \Pi\left(\left[\hat{\bar{\bar{M}}}\right]_{LHS}\right)} \mu_P\left[\left([\hat{M}'[\pi^{-1}(1)], \hat{M}'[\pi^{-1}(2)], \cdots, \hat{M}'[\pi^{-1}(N)]], [t_I, t_F]\right) \middle| (s^A, t_I)\right]$$

$$= \sum_{\pi \in \Pi\left(\left[\hat{\bar{\bar{M}}}\right]_{LHS}\right)} \left[\begin{array}{l} \displaystyle\int \cdots \int_{t_I = \tau(\pi^{-1}(0)) < \tau(\pi^{-1}(1)) < \cdots < \tau(\pi^{-1}(N)) < \tau(\pi^{-1}(N+1)) = t_F} \prod_{k=1}^{K} \left(d\tau(k,1) \cdots d\tau(k, N_k)\right) \\ \displaystyle\times \prod_{k=1}^{K} \left(\prod_{i_k=1}^{N_k} r\left(\hat{M}'[k, i_k]; s(\pi(k, i_k) - 1), \tau(k, i_k)\right)\right) \\ \displaystyle\times \exp\left\{ -\sum_{\nu=0}^{N} \int_{\tau(\pi^{-1}(\nu))}^{\tau(\pi^{-1}(\nu+1))} d\tau \, \delta R_X^{ID}(s(\nu), s^A, \tau) \right\} \Bigg|_{\langle s(0)| = \langle s^A|, \ \left\{\langle s(\nu)| = \langle s(\nu-1)|\hat{M}'[\pi^{-1}(\nu)] \,\middle|\, \nu = 1, ..., N\right\}} \end{array} \right].$$

--- Eq.(SA-2.1.1’)

Comparing Eq.(SA-2.1.1’) with Eq.(SA-2.1.2), we can see that the ansatz, Eq.(SM-2.8), should hold if and *nearly* only if the following two equations are satisfied.

(A) The equation between the multiple-time integration operations:

$$\sum_{\pi\in\Pi\left(\left[\vec{\vec{\hat{M}}}\right]_{LHS}\right)} \int\cdots\int_{t_I<\tau(\pi^{-1}(1))<\cdots<\tau(\pi^{-1}(N))<t_F} \left(\prod_{k=1}^{K} d\tau(k,1)\cdots d\tau(k,N_k)\right) \prod_{k=1}^{K} F_k\left(\tau(k,1),\ldots,\tau(k,N_k)\right)$$
$$= \prod_{k=1}^{K}\left(\int\cdots\int_{t_I<\tau(k,1)<\cdots<\tau(k,N_k)<t_F} d\tau(k,1)\cdots d\tau(k,N_k)\, F_k\left(\tau(k,1),\ldots,\tau(k,N_k)\right)\right),$$

--- Eq.(SA-2.1.3)

for *any* set of non-singular functions, $\left\{F_k\left(\tau(k,1),\ldots,\tau(k,N_k)\right) \mid k=1,\ldots,K\right\}$. Because the both sides share the same integrands and the dummy time variables for integration, it is tantamount to the equation between the domains of integration.

(B) The equation between the integrands (*i.e.*, the probability densities):

$$\left(\prod_{k=1}^{K}\prod_{i_k=1}^{N_k} r\left(\hat{M}'[k,i_k];\, s(\pi(k,i_k)-1),\, \tau(k,i_k)\right)\right)\Bigg|_{\substack{\langle s(0)|=\langle s^A|,\\ \left\{\langle s(\nu)|=\langle s(\nu-1)|\hat{M}'[\pi^{-1}(\nu)]\,|\, \nu=1,\ldots,N\right\}}}$$
$$\times\exp\left\{-\sum_{\nu=0}^{N}\int_{\tau(\pi^{-1}(\nu))}^{\tau(\pi^{-1}(\nu+1))} d\tau\, \delta R_X^{ID}(s(\nu),\, s^A,\, \tau)\right\}\Bigg|_{\substack{\langle s(0)|=\langle s^A|,\\ \left\{\langle s(\nu)|=\langle s(\nu-1)|\hat{M}'[\pi^{-1}(\nu)]\,|\, \nu=1,\ldots,N\right\}}},$$
$$=\left(\prod_{k=1}^{K}\left[\prod_{i_k}^{N_k} r\left(\hat{M}[k,i_k];\, s_{i_k-1},\, \tau(k,i_k)\right)\right]\Bigg|_{\substack{\langle s_0|=\langle s^A|,\\ \left\{\langle s_{i_k}|=\langle s_{i_k-1}|\hat{M}[k,i_k]\,|\, i_k=1,\ldots,N_k\right\}}}\right)$$
$$\times\exp\left\{-\sum_{k=1}^{K}\left[\sum_{i_k=0}^{N_k}\int_{\tau(k,i_k)}^{\tau(k,i_k+1)} d\tau\, \delta R_X^{ID}(s_{i_k},\, s^A,\, \tau)\right]\Bigg|_{\substack{\langle s_0|=\langle s^A|,\\ \left\{\langle s_{i_k}|=\langle s_{i_k-1}|\hat{M}[k,i_k]\,|\, i_k=1,\ldots,N_k\right\}}}\right\}$$

--- Eq.(SA-2.1.4)

for every map $\pi\in\Pi\left(\left[\vec{\vec{\hat{M}}}\right]_{LHS}\right)$ and its associated temporal order of events.

Eq.(SA-2.1.3) is an identity that is intuitively plausible. However, its rigorous proof is not so straightforward, and will be given in section SA-2.2. In contrast, Eq.(SA-2.1.4) holds only under an appropriate set of conditions on the indel rate parameters. Here, let us delve further into this equation.

The both sides of Eq.(SA-2.1.4) exhibit very similar forms. Each of them is a product of the rates of indels that actually occurred or their equivalents, multiplied by an exponential. And the exponent is the summation of time-integrated increments, of the exit rates of the states that the sequence actually (or virtually) went through, compared to the exit rate of the ancestral state. Thus, aside from miraculous, exceptional cases, it would be natural

to expect the equations to be satisfied for each of the factors. This reasoning gives two types of equations. One is a set of equations for the factors in the product,

$$r\left(\hat{M}'[k,i_k];\, s(\pi(k,i_k)-1),\, \tau(k,i_k)\right) = r\left(\hat{M}[k,i_k];\, s_{i_k-1},\, \tau(k,i_k)\right) \quad \text{--- Eq.(SA-2.1.4'a)}$$

for $^{\forall}k=1,...,K$, $^{\forall}i_k=1,...,N_k$, and $^{\forall}\pi \in \Pi\left(\left[\vec{\vec{\hat{M}}}\right]_{LHS}\right)$. And the other is an equation for the exponent,

$$\left\{\sum_{\nu=0}^{N}\int_{\tau(\pi^{-1}(\nu))}^{\tau(\pi^{-1}(\nu+1))} d\tau\, \delta R_X^{ID}(s(\nu), s^A, \tau)\right\}\Bigg|_{\langle s(0)|=\langle s^A|,\ \left\{\langle s(\nu)|=\langle s(\nu-1)|\hat{M}'[\pi^{-1}(\nu)] \,|\, \nu=1,...,N\right\}}$$
$$= \sum_{k=1}^{K}\left[\sum_{i_k=0}^{N_k}\int_{\tau(k,i_k)}^{\tau(k,i_k+1)} d\tau\, \delta R_X^{ID}(s_{i_k}, s^A, \tau)\right]\Bigg|_{\langle s_0|=\langle s^A|,\ \left\{\langle s_{i_k}|=\langle s_{i_k-1}|\hat{M}[k,i_k] \,|\, i_k=1,...,N_k\right\}} \quad \text{--- Eq.(SA-2.1.4'b)}$$

for $^{\forall}\pi \in \Pi\left(\left[\vec{\vec{\hat{M}}}\right]_{LHS}\right)$. Here, we set $\tau(\pi^{-1}(0)) \equiv t_I$ and $\tau(\pi^{-1}(N+1)) \equiv t_F$. In Eq.(SA-2.1.4'a), $s(\pi(k,i_k)-1)$ is the sequence state immediately before $\hat{M}'[k,i_k]$ in the global indel history, and s_{i_k-1} is the state immediately before $\hat{M}[k,i_k]$ in the isolated k th local indel history. The only difference between both sides of Eq.(SA-2.1.4'a) is in the states. In general, $s(\pi(k,i_k)-1)$ on the left-hand side resulted from some of the events in the other local indel histories, on top of $\hat{M}[k,j]$ with $j<i_k$. In contrast, s_{i_k-1} on the right hand side will never be impacted by the other local histories. Thus, Eq.(SA-2.1.4'a) simply states, for the PWA probability to be factorized, "the rate parameter for each indel operator in each local indel history must never be influenced by the actions of any indels that occurred before $\tau(k,i_k)$ and that belong to any other local histories ."

Meanwhile, Eq.(SA-2.1.4'b) appear more formidable than Eq.(SA-2.1.4'a). Nevertheless, we can prove the following proposition.

[Proposition SA-2.1.1]

"Let $\langle s\cdot[k,i_k]| \equiv \langle s|\hat{M}'[k,i_k]$ and $\langle s\cdot[k',i_{k'}]| \equiv \langle s|\hat{M}''[k',i_{k'}]$ (with $k, k'(\neq k) \in \{1,...,K\}$) be the states resulting from the actions of the equivalents of events $\hat{M}[k,i_k]$ and $\hat{M}[k',i_{k'}]$, respectively, on $s \in S^{II}$. And let

$\langle s\cdot[k,i_k][k',i_{k'}]| \equiv \langle s|\hat{M}'[k,i_k]\hat{M}'[k',i_{k'}] = \langle s|\hat{M}''[k',i_{k'}]\hat{M}''[k,i_k]$ be the state resulting from the

consecutive actions of the equivalents of $\hat{M}[k, i_k]$ and $\hat{M}[k', i_{k'}]$ on s. The equation for the exponents, Eq.(SA-2.1.4'b), holds for every global history $\pi \in \Pi\left(\left[\hat{\bar{\bar{M}}}\right]_{LHS}\right)$ and for each of its sub-histories that could occur in any sub-interval, $[t_I, t]$ with $t \in [t_I, t_F]$, if and only if the equation,

$$R_X^{ID}(s,t) + R_X^{ID}(s \cdot [k,i_k][k',i_{k'}],t) = R_X^{ID}(s \cdot [k,i_k],t) + R_X^{ID}(s \cdot [k',i_{k'}],t), \quad \text{--- Eq.(SA-2.1.5)}$$

holds for every pair, $\hat{M}[k, i_k]$ and $\hat{M}[k', i_{k'}]$ (with $k \neq k'$), in the LHS $\hat{\bar{\bar{M}}}$, for every possible state $s \in S^{II}$ before both equivalents of $\hat{M}[k, i_k]$ and of $\hat{M}[k', i_{k'}]$ in the global histories in $\Pi\left(\left[\hat{\bar{\bar{M}}}\right]_{LHS}\right)$, and at any time $t \in [t_I, t_F]$."

The detailed proof of this proposition is given in subsection SA-2.3. In the proposition, the applicable scope of Eq.(SA-2.1.4'b) was extended to all sub-histories of global histories belonging to $\Pi\left(\left[\hat{\bar{\bar{M}}}\right]_{LHS}\right)$ and to any sub-interval, $[t_I, t]$, of $[t_I, t_F]$. This extension would be acceptable in practical analyses, where what we actually want is to factorize *all* alignment probabilities during *any* time interval. We can clarify the meaning of Eq.(SA-2.1.5) by rewriting it as follows:

$$\delta R_X^{ID}(s \cdot [k,i_k][k',i_{k'}], s \cdot [k',i_{k'}], t) = \delta R_X^{ID}(s \cdot [k,i_k], s, t), \quad \text{--- Eq.(SA-2.1.5')}$$

$$\delta R_X^{ID}(s \cdot [k,i_k][k',i_{k'}], s \cdot [k,i_k], t) = \delta R_X^{ID}(s \cdot [k',i_{k'}], s, t). \quad \text{--- Eq.(SA-2.1.5'')}$$

These equations mean that the increment of the exit rate due to an event in a local indel history must be independent of the effect of any event in any other local indel history.

To summarize, we derived a sufficient and nearly necessary set of conditions, Eq.(SA-2.1.4'a) and Eq.(SA-2.1.5), under which the integrand of the probability of an indel history can be factorized, as in Eq.(SA-2.1.4). To clarify what these conditions mean, we here rephrase them in words. First, Eq.(SA-2.1.4'a) can be rephrased as follows.

Condition (i): "The rate parameter, $r\left(\hat{M}'[k,i_k]; s', \tau(k,i_k)\right)$, for each actually occurred indel event ($\hat{M}'[k,i_k]$) will not be influenced by the action of any indel events outside of the k th local history before $\tau(k,i_k)$."

Second, we can rephrase Eq.(SA-2.1.5) as follows.

Condition (ii): "Let $\langle s(\nu)| = \langle s^A | \hat{M}'[\pi^{-1}(1)] \cdots \hat{M}'[\pi^{-1}(\nu)]$ be the state resulting from the actions of events up to (and including) the ν th event in a global history corresponding to a

map $\pi \in \Pi\left(\left[\hat{\bar{\bar{M}}}\right]_{LHS}\right)$, and let $\hat{M}'[\pi^{-1}(\nu)] = \hat{M}'[k(\nu), i_{k(\nu)}(\nu)]$ be the ν th event in the global history. Then, the increment of the exit rate, $\delta R_X^{ID}(s(\nu), s(\nu-1), t)$, due to the event $\hat{M}'[\pi^{-1}(\nu)] = \hat{M}'[k(\nu), i_{k(\nu)}(\nu)]$, will not be influenced by the actions of any indel events outside of the $k(\nu)$ th local history before $\hat{M}'[\pi^{-1}(\nu)]$."

If this set of conditions is satisfied for all global indel histories in a LHS equivalence class $\left[\hat{\bar{\bar{M}}}\right]_{LHS}$, then, Eq.(SA-2.1.4) holds for all integrands. This, combined with the identity on the domains of integration, Eq.(SA-2.1.3), makes the total probability of $\left[\hat{\bar{\bar{M}}}\right]_{LHS}$ factorable, thus proving the ansatz, Eq.(SM-2.8). [NOTE: Someone might guess that the condition (ii) should follow from the condition (i) almost trivially. We will see that this naïve guess is wrong in subsection R8-2 of the main Results and Discussion.]

SA-2.2. Proof of factorization of multiple-time integration, Eq.(SA-2.1.3)

[This subsection was adapted from section A4 of Appendix of [32].]

The identity, Eq.(SA-2.1.3):

$$\sum_{\pi \in \Pi\left(\left[\hat{\bar{\bar{M}}}\right]_{LHS}\right)} \int\cdots\int_{t_I<\tau(\pi^{-1}(1))<\cdots<\tau(\pi^{-1}(N))<t_F} \left(\prod_{k=1}^{K} d\tau(k,1)\cdots d\tau(k,N_k)\right) \prod_{k=1}^{K} F_k\left(\tau(k,1),\ldots,\tau(k,N_k)\right)$$

$$= \prod_{k=1}^{K}\left(\int\cdots\int_{t_I<\tau(k,1)<\cdots<\tau(k,N_k)<t_F} d\tau(k,1)\cdots d\tau(k,N_k)\, F_k\left(\tau(k,1),\ldots,\tau(k,N_k)\right)\right),$$

--- Eq.(SA-2.2.1)

where $\left\{F_k\left(\tau(k,1),\ldots,\tau(k,N_k)\right) \mid k=1,\ldots,K\right\}$ is any set of non-singular functions of multiple-time points, is one of the two essential elements for obtaining our sufficient and nearly necessary set of conditions for a factorable PWA probability. The identity states that, if we sum the multiple-time integration operations for global indel histories over a LHS equivalence class, it can be factorized into the product of multiple-time integration operations, each for a local indel history, over the LHS. Here, we prove this identity in a mathematically rigorous manner.

Let us remember here that $\Pi\left(\left[\hat{\bar{\bar{M}}}\right]_{LHS}\right)$ denotes the set of maps that correspond to global indel histories in a LHS equivalence class. Each of its elements is expressed as:

$$\pi: \ (k, i_k) \ (k = 1, ..., K; i_k = 1, ..., N_k) \ \mapsto \ \nu \, (= 1, ..., N) .$$

Then, we first note that, because the integrands and the sets of variables of integration are identical on both sides of Eq.(SA-2.2.1), proving this identity is equivalent to proving the equation (*modulo differences of measure zero*) between the domains of integration:

$$\bigcup_{\pi \in \Pi\left(\left[\vec{\vec{\hat{M}}}\right]_{LHS}\right)} D^{(N)}\left[\pi\left(\vec{\vec{\hat{M}}}\right); [t_I, t_F]\right] = \mathop{\times}_{k=1}^{K} D^{(N_k)}\left[\vec{\hat{M}}[k]; [t_I, t_F]\right] . \quad \text{--- Eq.(SA-2.2.2)}$$

Here, $D^{(N_k)}\left[\vec{\hat{M}}[k]; [t_I, t_F]\right]$ is the domain of integration for the k th local indel history,

$\vec{\hat{M}}[k] \equiv \left[\hat{M}[k,1], ..., \hat{M}[k,N_k]\right]$:

$$D^{(N_k)}\left[\vec{\hat{M}}[k]; [t_I, t_F]\right] \equiv \left\{ \left(\tau(k,1), ..., \tau(k,N_k)\right) \,\middle|\, t_I < \tau(k,1) < \cdots < \tau(k,N_k) < t_F \right\} .$$

--- Eq.(SA-2.2.3a)

And $D^{(N)}\left[\pi\left(\vec{\vec{\hat{M}}}\right); [t_I, t_F]\right]$ is the domain of integration for the global indel history, $\pi\left(\vec{\vec{\hat{M}}}\right)$:

$$D^{(N)}\left[\pi\left(\vec{\vec{\hat{M}}}\right); [t_I, t_F]\right] \equiv \left\{ \begin{pmatrix} \tau(1,1), ..., \tau(1,N_1); \\ ....; \\ \tau(K,1), ..., \tau(K,N_K) \end{pmatrix} \middle| \ t_I < \tau(\pi^{-1}(1)) < \cdots < \tau(\pi^{-1}(N)) < t_F \right\} .$$

--- Eq.(SA-2.2.3b)

To go further, let us introduce a new notation, $\Pi^{(K)}[N_1, ..., N_K]$, that represents the set $\Pi\left(\left[\vec{\vec{\hat{M}}}\right]_{LHS}\right)$. The new notation reminds us that each of the $\frac{N!}{\prod_{k=1}^{K} N_k!}$ elements of $\Pi\left(\left[\vec{\vec{\hat{M}}}\right]_{LHS}\right)$ can be re-interpreted as a rearrangement of K sets, whose sizes are $N_1, ..., N_K$, into a single set of size $N = \sum_{k=1}^{N} N_k$. Then, each map $\pi^{(K)} \in \Pi\left(\left[\vec{\vec{\hat{M}}}\right]_{LHS}\right) = \Pi^{(K)}[N_1, ..., N_K]$ can be re-expressed as a composite map, $\pi^{(K)} = \rho \circ \left[(\pi^{(K-1)}, I_{N_k})\right]$. Here $\pi^{(K-1)} \in \Pi^{(K-1)}[N_1, ..., N_{K-1}]$ is a rearrangement of $K-1$ of the original K sets excluding the K th set, I_{N_K} is the identity map from the N_k elements in the K th set to themselves, and $\rho \in \Pi^{(2)}[N - N_K, N_K]$ is a rearrangement of the K th set and the remainder made from the $K-1$ sets. The numbers of the elements exactly match, because

we have $\frac{N!}{\prod_{k=1}^{K} N_k!} = \frac{N!}{(N-N_k)!N_k!} \times \frac{(N-N_k)!}{\prod_{k=1}^{K-1} N_k!}$. Provided that the binary (*i.e.*, $K=2$) version of Eq.(SA-2.2.2) is proved, then we can apply them for each fixed $\pi^{(K-1)} \in \Pi^{(K-1)}[N_1,...,N_{K-1}]$ and all $\rho \in \Pi^{(2)}[N-N_K, N_K]$, and we can factor out the contribution from the K th local (or "separated") indel history.

This is formally proved as follows. First, the left-hand side of Eq.(SA-2.2.2) is re-expressed as:

$$\bigcup_{\pi \in \Pi^{(K)}[N_1,...,N_K]} D^{(N)}\left[\pi\left(\hat{\vec{\vec{M}}}\right);[t_I,t_F]\right] = \bigcup_{\pi' \in \Pi^{(K-1)}[N_1,...,N_{K-1}]} \left\{ \bigcup_{\rho \in \Pi^{(2)}[N-N_K,N_K]} D^{(N)}\left[\rho \circ \left[(\pi', I_{N_K})\right]\left(\hat{\vec{\vec{M}}}\right);[t_I,t_F]\right]\right\}.$$

--- Eq.(SA-2.2.4)

On the right-hand side, we have $\rho \circ \left[(\pi', I_{N_K})\right]\left(\hat{\vec{\vec{M}}}\right) = \rho\left(\left[\pi'\left(\hat{\vec{\vec{M}}}'\right), \hat{\vec{M}}[K]\right]\right)$ by definition. Here, $\hat{\vec{\vec{M}}}' = \left[\hat{\vec{M}}[1],...,\hat{\vec{M}}[K-1]\right]$ is the "reduced" LHS consisting of $K-1$ out of the original K local indel histories in $\hat{\vec{\vec{M}}}$, excluding the K th local history, $\hat{\vec{M}}[K]$. Substituting this into Eq.(SA-2.2.4), and assuming that Eq.(SA-2.2.2) holds with $K=2$, we have:

$$\begin{aligned}
&\bigcup_{\pi \in \Pi^{(K)}[N_1,...,N_K]} D^{(N)}\left[\pi\left(\hat{\vec{\vec{M}}}\right);[t_I,t_F]\right] \\
&= \bigcup_{\pi' \in \Pi^{(K-1)}[N_1,...,N_{K-1}]} \left\{ D^{(N-N_k)}\left[\pi'\left(\hat{\vec{\vec{M}}}'\right);[t_I,t_F]\right] \times D^{(N_k)}\left[\hat{\vec{M}}[k];[t_I,t_F]\right]\right\} \qquad \text{--- Eq.(SA-2.2.5)} \\
&= \left\{ \bigcup_{\pi' \in \Pi^{(K-1)}[N_1,...,N_{K-1}]} D^{(N-N_k)}\left[\pi'\left(\hat{\vec{\vec{M}}}'\right);[t_I,t_F]\right]\right\} \times D^{(N_k)}\left[\hat{\vec{M}}[k];[t_I,t_F]\right].
\end{aligned}$$

This series of equations re-expresses the above verbal reasoning in clear mathematical terms, and formally demonstrates that the domain of integration for the rightmost local indel history (*i.e.*, the K th local history) is indeed factored out. Iteratively applying the above reasoning to the remaining set of $K-1$ local indel histories, we can prove that the domains of integration for all local indel histories can be factored out. This finally gives Eq.(SA-2.2.2) and thus proves the identity, Eq.(SA-2.2.1), *i.e.*, Eq.(SA-2.1.3). Thus, the problem at hand was reduced to proving Eq.(SA-2.2.2) with $K=2$, which we will call the "binary domain identity" here. It is rewritten as:

$$\bigcup_{\rho\in\Pi^{(2)}[N_1,N_2]} D^{(N_1+N_2)}\left[\rho\left(\left[\vec{\hat{M}}[1],\vec{\hat{M}}[2]\right]\right);[t_I,t_F]\right] = D^{(N_1)}\left[\vec{\hat{M}}[1];[t_I,t_F]\right]\times D^{(N_2)}\left[\vec{\hat{M}}[2];[t_I,t_F]\right].$$

--- Eq.(SA-2.2.6)

Using Eqs.(SA-2.2.3a,b), and setting $\tau_i \equiv \tau(1,i)$ and $\tau_i' \equiv \tau(2,i)$, it can be rewritten further as:

$$\bigcup_{\rho\in\Pi^{(2)}[N_1,N_2]} \left\{(\tau_1,...,\tau_{N_1};\tau_1',...,\tau_{N_2}') \,\middle|\, t_I < \tau(\rho^{-1}(1)) < \cdots < \tau(\rho^{-1}(N_1+N_2)) < t_F\right\}$$

$$= \left\{(\tau_1,...,\tau_{N_1}) \,\middle|\, t_I < \tau_1 < \cdots < \tau_{N_1} < t_F\right\} \times \left\{(\tau_1',...,\tau_{N_2}') \,\middle|\, t_I < \tau_1' < \cdots < \tau_{N_2}' < t_F\right\} .$$

--- Eq.(SA-2.2.6')

(It should be noted that, in this subsection, the identities between the domains are considered *modulo differences of measure zero*.)

We will prove this identity, Eq.(SA-2.2.6'), *via* mathematical induction regarding N_2. First, we show Eq.(SA-2.2.6') with $N_2 = 1$ holds for every fixed positive integer N_1. In this case, $\Pi^{(2)}[N_1, N_2 = 1]$ consists of $N_1 + 1$ elements, each of which inserts the event in the 2nd local history between the i th and $i+1$ th events in the 1st local history ($i = 1,...,N_1 - 1$), or places it before or after all events in the 1st local history. Thus, we have:

$$\bigcup_{\rho\in\Pi^{(2)}[N_1,1]} \left\{(\tau_1,...,\tau_{N_1};\tau_1') \,\middle|\, t_I < \tau(\rho^{-1}(1)) < \cdots < \tau(\rho^{-1}(N_1+1)) < t_F\right\}$$

$$= \bigcup_{i=0}^{N_1} \left\{(\tau_1,...,\tau_{N_1};\tau_1') \,\middle|\, t_I < \tau_1 < \cdots < \tau_{N_1} < t_F,\ \tau_i < \tau_1' < \tau_{i+1}\right\}$$

$$= \left\{(\tau_1,...,\tau_{N_1};\tau_1') \,\middle|\, t_I < \tau_1 < \cdots < \tau_{N_1} < t_F,\ \bigcup_{i=0}^{N_1}\left\{\tau_i < \tau_1' < \tau_{i+1}\right\}\right\}$$

$$= \left\{(\tau_1,...,\tau_{N_1};\tau_1') \,\middle|\, t_I < \tau_1 < \cdots < \tau_{N_1} < t_F,\ t_I < \tau_1' < t_F\right\}$$

$$= \left\{(\tau_1,...,\tau_{N_1}) \,\middle|\, t_I < \tau_1 < \cdots < \tau_{N_1} < t_F\right\} \times \left\{(\tau_1') \,\middle|\, t_I < \tau_1' < t_F\right\}.$$

Here we set $\tau_0 \equiv t_I$ and $\tau_{N_1+1} \equiv t_F$. This shows that Eq.(SA-2.2.6') with $N_2 = 1$ holds for every $N_1 \in \mathrm{N}_1$ (N_1 is the set of positive integers).

Next, let us assume that the binary domain identity, Eq.(SA-2.2.6'), holds for $N_2 = N$ and for every $N_1 \in \mathrm{N}_1$, and see if the identity also holds for $N_2 = N + 1$. For this purpose, we classify $\rho \in \Pi^{(2)}[N_1, N+1]$ according to the position of τ_{N+1}' relative to $\tau_1, ..., \tau_{N_1}$, and let $\Pi'^{(2)}[N_1, N+1; i]$ (with $i = 0,1,...,N_1$) be the subset of $\Pi^{(2)}[N_1, N+1]$ whose elements satisfy $\tau_i < \tau_{N+1}' < \tau_{i+1}$. Here we set $\tau_0 \equiv t_I$ and $\tau_{N_1+1} \equiv t_F$ again. For every $\rho \in \Pi'^{(2)}[N_1, N+1; i]$, there exist a unique $\sigma \in \Pi^{(2)}[i, N]$ such that: $\tau\left(\rho^{-1}(\nu)\right) = \tau\left(\sigma^{-1}(\nu)\right)$ for $\nu = 1,..., N+i$, $= \tau_{N+1}'$ for $\nu = N+i+1$, and $= \tau_{\nu-N-1}$ for $\nu = N+i+2,..., N+N_1+1$. It could also be represented as:

$$\left(\tau\left(\rho^{-1}(1)\right),...,\tau\left(\rho^{-1}(N+N_1+1)\right)\right)=\left(\tau\left(\sigma^{-1}(1)\right),...,\tau\left(\sigma^{-1}(N+i)\right),\tau'_{N+1},\tau_{i+1},...,\tau_{N_1}\right).$$

-- Eq.(SA-2.2.7)

Thus, $\sigma\in\Pi^{(2)}[i,N]$ corresponds to the sub-history before τ'_{N+1}. Taking advantage of these facts, the left-hand side of Eq.(SA-2.2.6') with $N_2=N+1$ is re-expressed as:

$$\bigcup_{\rho\in\Pi^{(2)}[N_1,N+1]}\left\{(\tau_1,...,\tau_{N_1};\tau'_1,...,\tau'_{N+1})\,\middle|\,t_I<\tau(\rho^{-1}(1))<\cdots<\tau(\rho^{-1}(N_1+N_2+1))<t_F\right\}$$
$$=\bigcup_{i=0}^{N_1}\left[\bigcup_{\rho\in\Pi'^{(2)}[N_1,N+1;i]}\left\{(\tau_1,...,\tau_{N_1};\tau'_1,...,\tau'_{N+1})\,\middle|\,t_I<\tau(\rho^{-1}(1))<\cdots<\tau(\rho^{-1}(N_1+N_2+1))<t_F\right\}\right]$$
$$=\bigcup_{i=0}^{N_1}\left[\bigcup_{\sigma\in\Pi^{(2)}[i,N]}\left\{(\tau_1,...,\tau_{N_1};\tau'_1,...,\tau'_{N+1})\,\middle|\,t_I<\tau(\sigma^{-1}(1))<\cdots<\tau(\sigma^{-1}(N+i))<\tau'_{N+1}<\tau_{i+1}<\cdots<\tau_{N_1}<t_F\right\}\right].$$

--- Eq.(SA-2.2.8)

Applying the assumed Eq.(SA-2.2.6') with $N_2=N$ and $N_1=i$, and with t_F replaced by τ'_{N+1}, to the expression in the square bracket on the rightmost hand side of Eq.(SA-2.2.8), we have:

$$\bigcup_{\sigma\in\Pi^{(2)}[i,N]}\left\{(\tau_1,...,\tau_{N_1};\tau'_1,...,\tau'_{N+1})\,\middle|\,t_I<\tau(\sigma^{-1}(1))<\cdots<\tau(\sigma^{-1}(N+i))<\tau'_{N+1}<\tau_{i+1}<\cdots<\tau_{N_1}<t_F\right\}$$
$$=\left\{(\tau_1,...,\tau_{N_1};\tau'_1,...,\tau'_{N+1})\,\middle|\,\begin{array}{l}t_I<\tau_1<\cdots<\tau_i<\tau'_{N+1}<\tau_{i+1}<\cdots<\tau_{N_1}<t_F,\\ t_I<\tau'_1<\cdots<\tau'_N<\tau'_{N+1}\end{array}\right\}$$
$$=\left\{(\tau_1,...,\tau_{N_1};\tau'_1,...,\tau'_{N+1})\,\middle|\,\begin{array}{l}t_I<\tau_1<\cdots<\tau_i<\tau_{i+1}<\cdots<\tau_{N_1}<t_F,\\ t_I<\tau'_1<\cdots<\tau'_N<\tau'_{N+1},\ \tau_i<\tau'_{N+1}<\tau_{i+1}\end{array}\right\}.$$

--- Eq.(SA-2.2.9)

Substituting Eq.(SA-2.2.9) back into the rightmost hand side of Eq.(SA-2.2.8), we finally get:

$$\bigcup_{\rho\in\Pi^{(2)}[N_1,N+1]}\left\{(\tau_1,...,\tau_{N_1};\tau'_1,...,\tau'_{N+1})\,\middle|\,t_I<\tau(\rho^{-1}(1))<\cdots<\tau(\rho^{-1}(N_1+N_2+1))<t_F\right\}$$
$$=\bigcup_{i=0}^{N_1}\left\{(\tau_1,...,\tau_{N_1};\tau'_1,...,\tau'_{N+1})\,\middle|\,\begin{array}{l}t_I<\tau_1<\cdots<\tau_i<\tau_{i+1}<\cdots<\tau_{N_1}<t_F,\\ t_I<\tau'_1<\cdots<\tau'_N<\tau'_{N+1},\ \tau_i<\tau'_{N+1}<\tau_{i+1}\end{array}\right\}$$
$$=\left\{(\tau_1,...,\tau_{N_1};\tau'_1,...,\tau'_{N+1})\,\middle|\,\begin{array}{l}t_I<\tau_1<\cdots<\tau_{N_1}<t_F,\\ t_I<\tau'_1<\cdots<\tau'_N<\tau'_{N+1},\ \bigcup_{i=0}^{N_1}\left\{\tau'_{N+1}\,\middle|\,\tau_i<\tau'_{N+1}<\tau_{i+1}\right\}\end{array}\right\}$$
$$=\left\{(\tau_1,...,\tau_{N_1};\tau'_1,...,\tau'_{N+1})\,\middle|\,\begin{array}{l}t_I<\tau_1<\cdots<\tau_{N_1}<t_F,\\ t_I<\tau'_1<\cdots<\tau'_N<\tau'_{N+1},\ t_I<\tau'_{N+1}<t_F\end{array}\right\}$$

$$= \left\{ (\tau_1,...,\tau_{N_1}; \tau'_1,...,\tau'_{N+1}) \mid t_I < \tau_1 < \cdots < \tau_{N_1} < t_F, \quad t_I < \tau'_1 < \cdots < \tau'_{N+1} < t_F \right\}$$

$$= \left\{ (\tau_1,...,\tau_{N_1}) \mid t_I < \tau_1 < \cdots < \tau_{N_1} < t_F \right\} \times \left\{ (\tau'_1,...,\tau'_{N+1}) \mid t_I < \tau'_1 < \cdots < \tau'_{N+1} < t_F \right\} .$$

--- Eq.(SA-2.2.10)

This final expression is nothing other than the right-hand side of Eq.(SA-2.2.6') with $N_2 = N+1$. Thus, assuming that Eq.(SA-2.2.6') holds for $N_2 = N$ and for every $N_1 \in \mathrm{N}_1$, we did indeed show that it also holds for $N_2 = N+1$ and for every $N_1 \in \mathrm{N}_1$. Therefore, the binary domain identity, Eq.(SA-2.2.6'), holds for every pair, $(N_1, N_2) \in \mathrm{N}_1 \times \mathrm{N}_1$. This completes the proof of our key identity, Eq.(SA-2.2.2), and therefore the proof of the factorization of the multiple-time integration, Eq.(SA-2.2.1).

SA-2.3. Proof of proposition SA-2.1.1 for factorization of exponential factor

[This subsection was adapted from section A5 of Appendix of [32].]

The other core element for factorable PWA probabilities is the proposition SA-2.1.1:

"Let $\langle s \cdot [k, i_k] | \equiv \langle s | \hat{M}'[k, i_k]$ and $\langle s \cdot [k', i_{k'}] | \equiv \langle s | \hat{M}''[k', i_{k'}]$ be the states resulting from the actions of the equivalents of events $\hat{M}[k, i_k]$ and $\hat{M}[k', i_{k'}]$, respectively, on $s \in S^{II}$. And let $\langle s \cdot [k, i_k][k', i_{k'}] | \equiv \langle s | \hat{M}'[k, i_k] \hat{M}'[k', i_{k'}] = \langle s | \hat{M}''[k', i_{k'}] \hat{M}''[k, i_k]$ be the state resulting from the consecutive actions of the equivalents of $\hat{M}[k, i_k]$ and $\hat{M}[k', i_{k'}]$ on s. The equation for the exponents, Eq.(SA-2.1.4'b), holds for every global history $\pi \in \Pi\left(\left[\hat{\bar{\bar{M}}}\right]_{LHS}\right)$ and for each of its sub-histories that could occur in any sub-interval, $[t_I, t]$ with $t \in [t_I, t_F]$, if and only if the equation,

$R_X^{ID}(s,t) + R_X^{ID}(s \cdot [k,i_k][k',i_{k'}],t) = R_X^{ID}(s \cdot [k,i_k],t) + R_X^{ID}(s \cdot [k',i_{k'}],t)$, --- Eq.(SA-2.1.5)

holds for every pair, $\hat{M}[k, i_k]$ and $\hat{M}[k', i_{k'}]$ (with $k \neq k'$), in the LHS $\hat{\bar{\bar{M}}}$, for every possible state $s \in S^{II}$ before the equivalents of $\hat{M}[k, i_k]$ and $\hat{M}[k', i_{k'}]$ in the global histories in $\Pi\left(\left[\hat{\bar{\bar{M}}}\right]_{LHS}\right)$, and at any time $t \in [t_I, t_F]$."

The proposition provides an essential part of our sufficient and nearly necessary set of conditions for the factorability of the PWA probability. Here, we prove this proposition via mathematical induction, similarly to the proof in subsection SA-2.2.

We first reduce the problem into a binary one by mathematical induction regarding the number of local indel histories, K. As in subsection SA-2.2, let $\Pi^{(K)}[N_1,..., N_K]$ denote

the set of maps, $\Pi\left(\left[\vec{\hat{\bar{M}}}\right]_{LHS}\right)$, each of whose elements is a rearrangement of K sets, of sizes $N_1, ..., N_K$, into a single set of size $N = \sum_{k=1}^{N} N_k$. And re-express each map $\pi \in \Pi^{(K)}[N_1, ..., N_K]$ as a composite map, $\pi = \rho \circ \left[(\sigma, I_{N_k})\right]$, where $\sigma \in \Pi^{(K-1)}[N_1, ..., N_{K-1}]$ and $\rho \in \Pi^{(2)}[N - N_K, N_K]$. Then, also as in subsection SA-2.2, we have $\rho \circ \left[(\sigma, I_{N_K})\right]\left(\vec{\hat{\bar{M}}}\right) = \rho\left(\left[\sigma\left(\vec{\hat{\bar{M}}}'\right), \vec{\hat{M}}[K]\right]\right)$ by definition, where $\vec{\hat{\bar{M}}}' = \left[\vec{\hat{M}}[1], ..., \vec{\hat{M}}[K-1]\right]$ is the reduced LHS defined below Eq.(SA-2.2.4). Thus, if the binary version of the proposition SA-2.1.1, with $\Pi\left(\left[\vec{\hat{\bar{M}}}\right]_{LHS}\right)$ replaced by $\Pi^{(2)}[N - N_K, N_K]$, is true for each fixed $\sigma \in \Pi^{(K-1)}[N_1, ..., N_{K-1}]$, we have the binary version of the factorization, Eq.(SA-2.1.4'b):

$$\left\{\sum_{\nu=0}^{N}\int_{\tau(\pi^{-1}(\nu))}^{\tau(\pi^{-1}(\nu+1))} d\tau\, \delta R_X^{ID}(s(\nu), s^A, \tau)\right\}\Bigg|_{\substack{\langle s(0)| = \langle s^A|, \\ \{\langle s(\nu)| = \langle s(\nu-1)|\hat{M}'[\pi^{-1}(\nu)] \mid \nu = 1,...,N\}}}$$

$$= \left\{\sum_{\nu'=0}^{N-N_K}\int_{\tau(\sigma^{-1}(\nu'))}^{\tau(\sigma^{-1}(\nu'+1))} d\tau\, \delta R_X^{ID}(s'(\nu'), s^A, \tau)\right\}\Bigg|_{\substack{\langle s'(0)| = \langle s^A|, \\ \{\langle s'(\nu)| = \langle s'(\nu'-1)|\hat{M}''[\sigma^{-1}(\nu')] \mid \nu' = 1,...,N-N_K\}}} \quad \text{--- Eq.(SA-2.3.1)}$$

$$+ \left[\sum_{i_K=0}^{N_K}\int_{\tau(k, i_K)}^{\tau(k, i_K+1)} d\tau\, \delta R_X^{ID}(s_{i_K}, s^A, \tau)\right]\Bigg|_{\substack{\langle s_0| = \langle s^A|, \\ \{\langle s_{i_K}| = \langle s_{i_K-1}|\hat{M}[k, i_K] \mid i_K = 1,...,N_K\}}}$$

for every possible $\pi = \rho \circ \left[(\sigma, I_{N_k})\right]$ with the fixed σ and any $\rho \in \Pi^{(2)}[N - N_K, N_K]$. The first summation on the right-hand side is the left-hand side of Eq.(SA-2.1.4'b) with $\pi \in \Pi^{(K)}[N_1, ..., N_K]$ replaced by $\sigma \in \Pi^{(K-1)}[N_1, ..., N_{K-1}]$. Thus, the problem was reduced to that of the factorization for the global indel histories equivalent to a set of $K-1$ local indel histories. By iteratively applying the binary version of the proposition SA-2.1.1 to the reduced problems, we will finally obtain the fully factorized form, *i.e.*, the right-hand side of Eq.(SA-2.1.4'b).

Therefore, all we have to do is prove the binary version of the proposition SA-2.1.1. To do so, we will rewrite it into a more tractable form. We first pick two integers, $i \in \{0,1,...,N_1\}$ and $j \in \{0,1,...,N_2\}$, and consider all sub-histories of indels composed of two local sub-histories, $\left[\hat{M}[1,1], ..., \hat{M}[1,i]\right]$ and $\left[\hat{M}[2,1], ..., \hat{M}[2,j]\right]$. (If $i = 0$ or $j = 0$,

the corresponding local sub-history is considered as empty.) Each such sub-history corresponds to a map, $\rho \in \Pi^{(2)}[i, j]$, and the state resulting from the action of this sub-history on the state $s^A \in S^{II}$ is represented, *e.g.*, as:

$\left\langle s^A \cdot \rho \right| \equiv \left\langle s^A \right| \hat{M}'[\rho^{-1}(1)] \cdots \hat{M}'[\rho^{-1}(i+j)]$. As in section R5 of the main Results and discussion, through the binary equivalence relations, Eq.(R5.2a-d), we can show that $\left\langle s^A \cdot \rho \right|$ for each sub-history $\rho \in \Pi^{(2)}[i, j]$ is in fact equal to the state:

$$\left\langle s[i;j] \right| \equiv \left\langle s^A \right| \left[\hat{M}[2,1] \cdots \hat{M}[2,j]\right] \left[\hat{M}[1,1] \cdots \hat{M}[1,i]\right] \left(\in S^{II}\right), \qquad \text{--- Eq.(SA-2.3.2a)}$$

that is uniquely determined solely by the local sub-histories, $\left[\hat{M}[1,1],...,\hat{M}[1,i]\right]$ and $\left[\hat{M}[2,1],...,\hat{M}[2,j]\right]$, and the initial state, $s^A \in S^{II}$. That is, the state $s^A \cdot \rho \; (= s[i;j])$ is independent of further details of $\rho \in \Pi^{(2)}[i, j]$. (Naturally, we have $s[0,0] = s^A$.) Thus, the binary version of the proposition SA-2.1.1 is rephrased as follows.

[Proposition SA-2.3.1]

"Eq.(SA-2.1.4'b) with $K = 2$ holds true for $^\forall \pi \in \Pi^{(2)}[N_1, N_2]$ and for each of their sub-histories during $[t_I, t]$ with $^\forall t \in (t_I, t_F)$ if and only if the equation,

$$R_X^{ID}(s[i-1; j-1], t) + R_X^{ID}(s[i;j], t) = \delta R_X^{ID}(s[i; j-1], t) + \delta R_X^{ID}(s[i-1; j], t),$$

--- Eq.(SA-2.3.2b)

holds for $^\forall i \in \{1,...,N_1\}$, $^\forall j \in \{1,...,N_2\}$, and for $^\forall t \in (t_I, t_F)$."

Here comes the proof of the proposition SA-2.3.1. First of all, we rewrite Eq. (SA-2.3.2b) in two different ways, as:

$$\delta R_X^{ID}(s[i;j], s[i;j-1], t) = \delta R_X^{ID}(s[i-1;j], s[i-1;j-1], t), \qquad \text{---Eq.(SA-2.3.2b')}$$

and

$$\delta R_X^{ID}(s[i;j], s[i-1;j], t) = \delta R_X^{ID}(s[i;j-1], s[i-1;j-1], t). \qquad \text{---Eq.(SA-2.3.2b'')}$$

These equations collectively indicate that the increment of the exit rate due to an indel event in one local indel history will not be influenced by the past events in the other local history. Indeed, these equations can be "solved" to give:

$$\delta R_X^{ID}(s[i;j], s[i;j-1], t) = \delta R_X^{ID}(s[0;j], s[0;j-1], t), \qquad \text{---Eq.(SA-2.3.3a)}$$

$$\delta R_X^{ID}(s[i;j], s[i-1;j], t) = \delta R_X^{ID}(s[i;0], s[i-1;0], t). \qquad \text{---Eq.(SA-2.3.3b)}$$

The right-hand sides of Eq.(SA-2.3.3a) and Eq.(SA-2.3.3b) are, respectively, the increment purely within the 2nd local history and that purely within the 1st local history. Replacing i

with i' in Eq.(SA-2.3.3b), and summing the result over $i'=1,..,i$, we find:

$$\delta R_X^{ID}(s[i;j],s[0;j],t)=\sum_{i'=1}^{i}\delta R_X^{ID}(s[i';j],s[i'-1;j],t)$$

$$=\sum_{i'=1}^{i}\delta R_X^{ID}(s[i';0],s[i'-1;0],t)\ =\delta R_X^{ID}(s[i;0],s[0;0],t).$$

Using $\delta R_X^{ID}(s[i;j],s[0;j],t)=\delta R_X^{ID}(s[i;j],s^A,t)-\delta R_X^{ID}(s[0;j],s^A,t)$ and $s[0,0]=s^A$, we get a key equation:

$$\delta R_X^{ID}(s[i;j],s^A,t)=\delta R_X^{ID}(s[i;0],s^A,t)+\delta R_X^{ID}(s[0;j],s^A,t). \quad \text{--- Eq.(SA-2.3.4)}$$

This means that the increment of the exit rate by a sub-history $\rho\in\Pi^{(2)}[i,j]$ is decomposed as the summation of two increments, each by one of the local sub-histories, $\left[\hat{M}[1,1],...,\hat{M}[1,i]\right]$ and $\left[\hat{M}[2,1],...,\hat{M}[2,j]\right]$.

Now, pick an indel history corresponding to a map $\pi\in\Pi^{(2)}[N_1,N_2]$, and consider the left-hand side of Eq.(SA-2.1.4'b) with $K=2$, *i.e.*, $\sum_{\nu=0}^{N}\int_{\tau(\pi^{-1}(\nu))}^{\tau(\pi^{-1}(\nu+1))}d\tau\ \delta R_X^{ID}(s(\nu),s^A,\tau)$ with $\langle s(0)|=\langle s^A|$ and $\langle s(\nu)|=\langle s(\nu-1)|\hat{M}'[\pi^{-1}(\nu)]$ for $\nu=1,...,N$. Let $i_k(\nu)$ ($k=1,2$) be the number of events in the local history $\left[\hat{M}[k,1],...,\hat{M}[k,N_k]\right]$ that are equivalent to those included in the sub-history $\left[\hat{M}'[\pi^{-1}(1)],...,\hat{M}'[\pi^{-1}(\nu)]\right]$ ($\nu=0,1,...,N$). Then, we have $i_1(\nu)+i_2(\nu)=\nu$, and $s(\nu)=s[i_1(\nu);i_2(\nu)]$. Thus, using Eq.(SA-2.3.4), the left-hand side of Eq.(SA-2.1.4'b) with $K=2$ can be decomposed into the contributions from two local sub-histories:

$$\sum_{\nu=0}^{N}\int_{\tau(\pi^{-1}(\nu))}^{\tau(\pi^{-1}(\nu+1))}d\tau\ \delta R_X^{ID}(s[i_1(\nu);0],s^A,\tau)\ +\ \sum_{\nu=0}^{N}\int_{\tau(\pi^{-1}(\nu))}^{\tau(\pi^{-1}(\nu+1))}d\tau\ \delta R_X^{ID}(s[0;i_2(\nu)],s^A,\tau).$$

--- Eq.(SA-2.3.5)

In each summation, $i_k(\nu)$ remains a particular value, *e.g.*, i_k, since $\nu=\pi([k,i_k])$ until (and excluding) $\nu=\pi([k,i_k+1])$ (for $k=1,2$). Taking account of it, Eq.(SA-2.3.5) becomes:

$$\sum_{i_1=0}^{N_1}\int_{\tau(1,i_1)}^{\tau(1,i_1+1)}d\tau\ \delta R_X^{ID}(s[i_1;0],s^A,\tau)\ +\ \sum_{i_2=0}^{N_2}\int_{\tau(2,i_2)}^{\tau(2,i_2+1)}d\tau\ \delta R_X^{ID}(s[0;i_2],s^A,\tau). \quad \text{--- Eq.(SA-2.3.5')}$$

From the definition of $s[i;j]$, Eq.(SA-2.3.2a), we can see that Eq.(SA-2.3.5') is nothing other than the right-hand side of Eq.(SA-2.1.4'b) with $K=2$. The argument after Eq.(SA-2.3.4) applies to every history corresponding to $\pi\in\Pi^{(2)}[N_1,N_2]$. Thus, we proved that Eq.(SA-2.1.4'b) with $K=2$ holds if Eq.(SA-2.3.2b) holds.

To prove the converse, we now assume that Eq.(SA-2.1.4'b) with $K = 2$ holds for the indel history corresponding to every $\pi \in \Pi^{(2)}[N_1, N_2]$, as well as for each of its sub-histories during $[t_I, t]$ with $^\forall t \in (t_I, t_F)$. Then, taking the time-derivative of both sides of Eq.(SA-2.1.4'b) with $K = 2$ for any incomplete time-interval $[t_I, t]$, we have, for a particular $\pi \in \Pi^{(2)}[N_1, N_2]$:

$$\delta R_X^{ID}(s(\nu), s^A, t) = \delta R_X^{ID}(s[i_1(\nu);0], s^A, t) + \delta R_X^{ID}(s[0;i_2(\nu)], s^A, t),$$

using the $i_k(\nu)$ ($k = 1, 2$) defined above. Because this equation holds for any time-interval $[t_I, t] \subset [t_I, t_F]$ and for every map $\pi \in \Pi^{(2)}[N_1, N_2]$, we get exactly Eq.(SA-2.3.4) for $^\forall i \in \{0,1,...,N_1\}$, $^\forall j \in \{0,1,...,N_2\}$, and for $^\forall t \in (t_I, t_F)$. Then it is easy to show Eq.(SA-2.3.2b). Starting with the right-hand side of Eq.(SA-2.3.2b), we find:

$$\delta R_X^{ID}(s[i;j-1], s^A, t) + \delta R_X^{ID}(s[i-1;j], s^A, t)$$
$$= \left\{\delta R_X^{ID}(s[i;0], s^A, t) + \delta R_X^{ID}(s[0;j-1], s^A, t)\right\} + \left\{\delta R_X^{ID}(s[i-1;0], s^A, t) + \delta R_X^{ID}(s[0;j], s^A, t)\right\}.$$

Swapping the 1st and 3rd terms on the right-hand side, we have:

$$\delta R_X^{ID}(s[i;j-1], s^A, t) + \delta R_X^{ID}(s[i-1;j], s^A, t)$$
$$= \left\{\delta R_X^{ID}(s[i-1;0], s^A, t) + \delta R_X^{ID}(s[0;j-1], s^A, t)\right\} + \left\{\delta R_X^{ID}(s[i;0], s^A, t) + \delta R_X^{ID}(s[0;j], s^A, t)\right\}$$
$$= \delta R_X^{ID}(s[i-1;j-1], s^A, t) + \delta R_X^{ID}(s[i;j], s^A, t) \ .$$

Adding $2R_X^{ID}(s^A, t)$ to the leftmost and rightmost sides of the above equation, we get Eq.(SA-2.3.2b). Thus, the converse was proved.

This proof of the proposition SA-2.3.1, combined with the proof above it, which resorts to the mathematical induction regarding K given the proposition SA-2.3.1, completes the proof of the key proposition SA-2.1.1.

SA-3. Total probability of LHS equivalence class under "long indel" model

[This section was adapted from section A6 of Appendix of [32].]

Here, we consider the "long indel" model [21]. Its indel rate parameters are given as follows:

$$r_I(x, l; s, t) = \begin{cases} \lambda_l & for \quad 1 \le x \le L(s) - 1, \\ \tilde{\lambda}_l^{(end)} & for \quad x = 0, L(s) \quad with \ \ L(s) > 0, \\ \tilde{\tilde{\lambda}}_l^{(whole)} & for \quad x = 0 \quad with \ \ L(s) = 0, \end{cases} \quad \text{--- Eq.(SA-3.1a)}$$

$$r_D(x_B, x_E; x, t) = \begin{cases} \mu_{l_D} & for \quad 1 \le x_B \le x_E \le L(s) - 1, \\ \tilde{\mu}_{l_D}^{(end)} & for \quad x_B = 1 \ or \ x_E = L(s), \\ \tilde{\tilde{\mu}}_{l_D}^{(whole)} & for \quad x_B = 1 \ and \ x_E = L(s). \end{cases} \quad \text{--- Eq.(SA-3.1b)}$$

Here $L(s)$ is the number of sites in the sequence state $s\left(\in S^{II}\right)$. And we set $l_D \equiv x_E - x_B + 1$, $\tilde{\mu}_l^{(end)} \equiv \sum_{l'=l}^{L_D^{CO}} \mu_{l'}$, and $\tilde{\tilde{\mu}}_l^{(whole)} \equiv \sum_{l'=l}^{L_D^{CO}} (l'-l+1)\mu_{l'}$. [NOTE: If the time reversibility is imposed on the model, the following equations also hold [21]: $\lambda_l = (\lambda_1/\mu_1)^l \mu_l$, $\tilde{\lambda}_l^{(end)} = (\lambda_1/\mu_1)^l \tilde{\mu}_l^{(end)}$ and $\tilde{\tilde{\lambda}}_l^{(whole)} = (\lambda_1/\mu_1)^l \tilde{\tilde{\mu}}_l^{(whole)}$. In this section, however, these equations do not play any particular roles.] The bulk parts of the above indel rates could be related to those in the indel model of Dawg [26] as follows:

$$\lambda_l = \lambda_I f_I(l), \quad \lambda_I = \sum_{l=1}^{L_I^{CO}} \lambda_l, \quad \mu_l = \lambda_D f_D(l), \quad \lambda_D = \sum_{l=1}^{L_I^{CO}} \mu_l \quad . \quad \text{--- Eq.(SA-3.2a,b,c,d)}$$

Here λ_I and λ_D are the total rates per site of bulk insertions and deletions, respectively, and $f_I(l)$ and $f_D(l)$ are the length distributions of insertions and deletions, respectively. (See also subsection R8-1 of the main Results and discussion.) Then, the exit rate under the "long indel" model can be calculated as:

$$R_X^{ID}(s,t) = (\lambda_I + \lambda_D)L(s) + \Delta^{Long}\left[\lambda_I, \{\tilde{\lambda}_l^{(end)}\}, \lambda_D, f_D(.)\right] \quad . \quad \text{--- Eq.(SA-3.2e)}$$

Here, $\Delta^{Long}\left[\lambda_I, \{\tilde{\lambda}_l^{(end)}\}, \lambda_D, f_D(.)\right] \equiv -\lambda_I + 2\left(\sum_{l=1}^{L_I^{CO}} \tilde{\lambda}_l^{(end)}\right) + \lambda_D(\bar{l}_D - 1)$, with $\bar{l}_D \equiv \sum_{l=1}^{L_D^{CO}} l\, f_D(l)$, is a constant that depends on the indel rate parameters.

Under this "long indel" model, we will calculate the total probability of a local-history-set (LHS) equivalence class of (global) indel histories, conditioned on a given ancestral sequence, according to the prescription proposed by Miklós et al. [21]. And we will show that the probability calculated this way is indeed identical to that calculated via our *ab initio* theoretical formulation.

We first briefly review the method of Miklós et al. [21]. In their method, each PWA is scanned from left to right, and horizontally partitioned into "chop-zones." In the bulk of the PWA, a chop-zone starts immediately on the right of a preserved ancestral site (PAS) and ends exactly at the next PAS. The leftmost chop-zone starts at the left-end of the PWA and ends exactly at the first PAS if at all, or otherwise ends at the right-end of the PWA. The rightmost chop-zone starts immediately on the right of the rightmost PAS, if at all, and ends at the right-end of the PWA. It should be noted that each chop-zone contains *at most* one PAS, and that the PAS contained in the chop-zone always resides at the right-end of the zone.

Conceptually, the conditional probability of each chop-zone (conditioned on the existence of the left-flanking PAS, if at all) is calculated by summing the contributions of all

local indel histories consistent with the homology structure [48] of the chop-zone. Then, according to the recipe of Miklós et al. [21], (the indel component of) the probability of a given PWA, conditioned on the ancestral sequence, is given as the product of the conditional probabilities over all chop-zones that make up the PWA. Therefore, by extension, Miklós et al.'s total probability of a LHS equivalence class of indel histories (consistent with the PWA) should be given by the product of the contributions from the local indel histories (including the empty histories), each confined in every chop-zone, over all chop-zones constituting the PWA. This is exactly what we will calculate in the following.

Now, as in section R6 of the main Results and discussion, consider a LHS equivalence class, $\left[\vec{\vec{\hat{M}}}\right]_{LHS}$ with $\vec{\vec{\hat{M}}} = \left\{\left[\hat{M}[k,1], ..., \hat{M}[k,N_k]\right]\right\}_{k=1,...,K}$, that is consistent with a given PWA, $\alpha(s^A, s^D)$, of an ancestral sequence (s^A) and its descendant (s^D). As near the bottom of section R6, we can define the regions of $\alpha(s^A, s^D)$ each of which potentially accommodates a local indel history, namely, $\gamma_1, \gamma_2, ..., \gamma_{\kappa_{\max}}$, as the region on the left of the leftmost PAS, the regions between two PASs next to each other, and the region on the right of the rightmost PAS. (Because the indel model at hand is space-homogeneous and has freely mutable flanking regions, every local indel history in each such region is independent of the histories outside, both physically and regarding the multiplication factor, as shown in Subsection R8-1 of the main Results and discussion.) Then, by appropriately distributing the local histories into such regions, we can provide a vector-representation of the LHS: $\vec{\vec{\hat{M}}} = \left(\vec{\hat{M}}[\gamma_1], \vec{\hat{M}}[\gamma_2], ..., \vec{\hat{M}}[\gamma_{\kappa_{\max}}]\right)$. Using these regions, each chop-zone of Miklós et al. [21] can be constructed by putting together a region γ_κ with its right-flanking PAS (for $\kappa = 1, ..., \kappa_{\max} - 1$), or by a region alone (for $\kappa = \kappa_{\max}$). According to Appendix A of [21], the contribution from the local history, $\vec{\hat{M}}[\gamma_\kappa] = \left[\hat{M}_1[\gamma_\kappa], ..., \hat{M}_{N_\kappa}[\gamma_\kappa]\right]$, in the chop-zone, $cz(\gamma_\kappa)$, that is associated with γ_κ is calculated as:

$$P_{Mik}\left[\left(\vec{\hat{M}}[\gamma_\kappa], [t_I, t_F]\right) \middle| \left(s^A[cz(\gamma_\kappa)], t_I\right)\right] = \prod_{\nu=1}^{N_\kappa} r(\hat{M}_\nu[\gamma_\kappa]; \phi_{\nu-1})$$
$$\times \left. \int\cdots\int_{t_I \equiv \tau_0 < \tau_1 < \cdots < \tau_{N_\kappa} < \tau_{N_\kappa+1} \equiv t_F} d\tau_1 \cdots d\tau_{N_\kappa} \exp\left\{-\sum_{\nu=0}^{N_\kappa} (\tau_{\nu+1} - \tau_\nu) R_{X(Mik)}^{ID}(\phi_\nu; cz(\gamma_\kappa))\right\}\right|_{\substack{\phi_0 = s^A[cz(\gamma_\kappa)], \\ \left\{\langle\phi_\nu| = \langle\phi_{\nu-1}|\hat{M}_\nu[\gamma_\kappa] \,|\, \nu=1,...,N_\kappa\right\}}}.$$

--- Eq.(SA-3.3)

Here, $s^A[cz(\gamma_\kappa)]$ is the portion of the ancestral state confined in the chop-zone $cz(\gamma_\kappa)$, and $\phi_0 (= s^A[cz(\gamma_\kappa)]), \phi_1, ..., \phi_{N_\kappa}$ are the chop-zone-confined states that the local indel history went

through. The expression is quite similar to each term in the perturbation expansion, Eq.(R4.7). Because each indel rate, $r(\hat{M}_v[\gamma_\kappa]; \phi_{v-1})$, is independent of time, it was put outside of the multiple-time integration. And, because each "exit rate," $R^{ID}_{X(Mik)}(\phi_v; cz(\gamma_\kappa))$ (detailed later), is also time-independent, its time integration (in the exponent) was reduced to a simple multiplication by the time-lapse ($\tau_{v+1} - \tau_v$). The "exit rate" $R^{ID}_{X(Mik)}(\phi_v; cz(\gamma_\kappa))$ needs some explanation. Because each chop zone (except $cz(\gamma_1)$) is defined conditionally on the PAS that is left-flanking the zone, and *because we now know that the probability is factorable* (see subsection R8-1 in the main Results and discussion), we do not have to consider deletions that pierce this PAS. Neither do we have to consider indel events completely outside of the chop zone. Therefore, taking advantage of the space-homogeneity of the indel rates, using the relationship with Dawg's indel model [26], Eqs.(SA-3.2a,b,c,d), and letting $L(\phi_v)$ be the number of sites in the state ϕ_v (including the PAS at the right-end of the zone, if at all), the "exit rate" $R^{ID}_{X(Mik)}(\phi_v; cz(\gamma_\kappa))$ according to Miklos et al.'s definition is expressed as:

$$R^{ID}_{X(Mik)}(\phi_v; cz(\gamma_\kappa)) = \sum_{x=0}^{L(\phi_v)-1} \sum_{l=1}^{L_I^{CO}} \lambda_I f_I(l) + \sum_{x=1}^{L(\phi_v)} \sum_{l=1}^{L_D^{CO}} \lambda_D f_D(l) \quad \text{--- Eq.(SA-3.4a)}$$

for $\kappa = 2, ..., \kappa_{\max} - 1$. It should be noted that the summation over the insertion positions (x) has the upper bound $x = L(\phi_v) - 1$, because an insertion on the immediate right of $x = L(\phi_v)$ belongs to the right-neighboring chop-zone ($cz(\gamma_{\kappa+1})$). The summation over the indel lengths (l's) is easily performed, and we get:

$$R^{ID}_{X(Mik)}(\phi_v; cz(\gamma_\kappa)) = (\lambda_I + \lambda_D) L(\phi_v) \quad for \quad \kappa = 2, ..., \kappa_{\max} - 1. \quad \text{--- Eq.(SA-3.4a')}$$

When $\kappa = \kappa_{\max} (\neq 1)$, the expression of $R^{ID}_{X(Mik)}(\phi_v; cz(\gamma_\kappa))$ is almost the same as Eq.(SA-3.4a); the only difference is that it also needs to include the insertions right-flanking the PWA (i.e., with $x = L(\phi_v)$), whose rates are given by $\tilde{\lambda}_l^{(end)}$ in Eq.(SA-3.1a). Thus, we have:

$$R^{ID}_{X(Mik)}(\phi_v; \gamma_{\kappa_{\max}}) = (\lambda_I + \lambda_D) L(\phi_v) + \sum_{l=1}^{L_I^{CO}} \tilde{\lambda}_l^{(end)} \quad for \quad \kappa_{\max} \neq 1. \quad \text{--- Eq.(SA-3.4b)}$$

When $\kappa = 1 (\neq \kappa_{\max})$, Eq.(SA-3.4a) is still useful, but we need two modifications, both because this chop-zone is not left-flanked by a PAS. First, insertions on the left-end (*i.e.*, with $x = 0$) must have the rates $\tilde{\lambda}_l^{(end)}$, again given in Eq.(SA-3.1a). Second, deletions "starting" at $x = 1$ must have the rates $\tilde{\mu}_l^{(end)} \left(= \sum_{l'=l}^{L_D^{CO}} \mu_{l'} \right)$ (in Eq.(SA-3.1b)). Taking account of these modifications, we have:

$$R^{ID}_{X(Mik)}(\phi_v; cz(\gamma_1)) = (\lambda_I + \lambda_D)(L(\phi_v) - 1) + \sum_{l=1}^{L_I^{CO}} \tilde{\lambda}_l^{(end)} + \sum_{l=1}^{L_D^{CO}} \tilde{\mu}_l^{(end)} \quad \text{--- Eq.(SA-3.4c)}$$

when $\kappa_{\max} \neq 1$. Because $\sum_{l=1}^{L_D^{CO}} \tilde{\mu}_l^{(end)} = \sum_{l=1}^{L_D^{CO}} \sum_{l'=l}^{L_D^{CO}} \mu_{l'} = \sum_{l'=1}^{L_D^{CO}} \sum_{l=1}^{l'} \mu_{l'} = \sum_{l'=1}^{L_D^{CO}} l' \mu_{l'} = \bar{l}_D \lambda_D$, we get:

$$R_{X(Mik)}^{ID}(\phi_\nu; cz(\gamma_1)) = (\lambda_I + \lambda_D) L(\phi_\nu) - \lambda_I + \sum_{l=1}^{L_I^{CO}} \tilde{\lambda}_l^{(end)} + \lambda_D(\bar{l}_D - 1). \qquad \text{--- Eq.(SA-3.4c')}$$

From Eqs.(SA-3.4a',b,c'), we find that $R_{X(Mik)}^{ID}(\phi_\nu; cz(\gamma_\kappa))$'s are always affine functions of $L(\phi_\nu)$ with the slope $(\lambda_I + \lambda_D)$, which is the same as that of the exit rate, $R_X^{ID}(s,t)$ given by Eq.(SA-3.2e), for the evolution of an *entire* sequence under the "long indel" model. Thus, we have:

$$\delta R_{X(Mik)}^{ID}(\phi_\nu, \phi_{\nu-1}; cz(\gamma_\kappa)) \equiv R_{X(Mik)}^{ID}(\phi_\nu; cz(\gamma_\kappa)) - R_{X(Mik)}^{ID}(\phi_{\nu-1}; cz(\gamma_\kappa)) = (\lambda_I + \lambda_D)\delta l\left(\hat{M}_\nu[\gamma_\kappa]\right),$$

--- Eq.(SA-3.5)

where $\delta l\left(\hat{M}_\nu[\gamma_\kappa]\right)$ is the change in $L(\phi_\nu)$ caused by the event $\hat{M}_\nu[\gamma_\kappa]$. This is exactly the same as the increment of the (actually time-independent) exit-rate:

$$\delta R_X^{ID}(s \cdot \hat{M}_\nu[\gamma_\kappa], s, t) \equiv R_X^{ID}(s \cdot \hat{M}_\nu[\gamma_\kappa], t) - R_X^{ID}(s,t) = (\lambda_I + \lambda_D)\delta l\left(\hat{M}_\nu[\gamma_\kappa]\right),$$

--- Eq.(SA-3.6)

caused by the event $\hat{M}_\nu[\gamma_\kappa]$ on the entire sequence. By successively applying $\hat{M}_{\nu'}[\gamma_\kappa]$ ($\nu' = 1,...,\nu$), we have:

$$\delta R_{X(Mik)}^{ID}(\phi_\nu, \phi_0; cz(\gamma_\kappa)) = \delta R_X^{ID}(s_\nu, s^A)\Big|_{\langle s_\nu| = \langle s^A|\hat{M}_1[\gamma_\kappa]\cdots\hat{M}_\nu[\gamma_\kappa]}. \qquad \text{--- Eq.(SA-3.7)}$$

Therefore, we can rewrite the exponent in Eq.(SA-3.3) as:

$$\begin{aligned}
&-\sum_{\nu=0}^{N_\kappa}(\tau_{\nu+1} - \tau_\nu)\, R_{X(Mik)}^{ID}(\phi_\nu; cz(\gamma_\kappa)) \\
&= -(\tau_{N_\kappa+1} - \tau_0) R_{X(Mik)}^{ID}(\phi_0; cz(\gamma_\kappa)) - \sum_{\nu=0}^{N_\kappa}(\tau_{\nu+1} - \tau_\nu)\, \delta R_{X(Mik)}^{ID}(\phi_\nu, \phi_0; cz(\gamma_\kappa)) \\
&= -(\tau_{N_\kappa+1} - \tau_0) R_{X(Mik)}^{ID}(\phi_0; cz(\gamma_\kappa)) - \left[\sum_{\nu=0}^{N_\kappa}(\tau_{\nu+1} - \tau_\nu)\, \delta R_X^{ID}(s_\nu, s^A)\right]\Bigg|_{\substack{s_0 = s^A \\ \{\langle s_\nu| = \langle s_{\nu-1}|\hat{M}_\nu[\gamma_\kappa] \,|\, \nu=1,...,N_\kappa\}}}.
\end{aligned}$$

--- Eq.(SA-3.8)

Substituting this back into the right hand side of Eq.(SA-3.3), and comparing the result with Eq.(SM-2.7) in Additional file 1, we have:

$$P_{Mik}\left[\left(\vec{\hat{M}}[\gamma_\kappa],[t_I,t_F]\right)\Big|\,(s^A[cz(\gamma_\kappa)],t_I)\right]$$
$$=\exp\left\{-(t_F-t_I)\,R^{ID}_{X(Mik)}(s^A[cz(\gamma_\kappa)];cz(\gamma_\kappa))\right\}\mu_P\left[\left(\vec{\hat{M}}[\gamma_\kappa],[t_I,t_F]\right)\Big|\,(s^A,t_I)\right].$$

--- Eq.(SA-3.9)

According to the method of Miklós et al. [21], we will *define* the total probability of the LHS equivalence class, $\left[\vec{\vec{\hat{M}}}\right]_{LHS}$ with $\vec{\vec{\hat{M}}}=\left(\vec{\hat{M}}[\gamma_1],\vec{\hat{M}}[\gamma_2],...,\vec{\hat{M}}[\gamma_{\kappa_{\max}}]\right)$, as:

$$P_{Mik}\left[\left(\left[\vec{\vec{\hat{M}}}\right]_{LHS},[t_I,t_F]\right)\Big|\,(s^A,t_I)\right]\equiv\prod_{\kappa=1}^{\kappa_{\max}}P_{Mik}\left[\left(\vec{\hat{M}}[\gamma_\kappa],[t_I,t_F]\right)\Big|\,(s^A[cz(\gamma_\kappa)],t_I)\right].$$

--- Eq.(SA-3.10)

Substituting Eq.(SA-3.9) into Eq.(SA-3.10) yields:

$$P_{Mik}\left[\left(\left[\vec{\vec{\hat{M}}}\right]_{LHS},[t_I,t_F]\right)\Big|\,(s^A,t_I)\right]$$
$$=\exp\left\{-(t_F-t_I)\sum_{\kappa=1}^{\kappa_{\max}}R^{ID}_{X(Mik)}(s^A[cz(\gamma_\kappa)];cz(\gamma_\kappa))\right\}\prod_{\kappa'=1}^{\kappa_{\max}}\mu_P\left[\left(\vec{\hat{M}}[\gamma_{\kappa'}],[t_I,t_F]\right)\Big|\,(s^A,t_I)\right].$$

--- Eq.(SA-3.10')

Substituting Eqs.(SA-3.4a',b,c') into the summation in the exponent on the right hand side, we get:

$$\sum_{\kappa=1}^{\kappa_{\max}}R^{ID}_{X(Mik)}(s^A[cz(\gamma_\kappa)];cz(\gamma_\kappa))$$
$$=(\lambda_I+\lambda_D)\sum_{\kappa=1}^{\kappa_{\max}}L(s^A[cz(\gamma_\kappa)])+\left\{-\lambda_I+2\left(\sum_{l=1}^{L_I^{CO}}\tilde{\lambda}_l^{(end)}\right)+\lambda_D(\bar{l}_D-1)\right\}.$$

--- Eq.(SA-3.11)

On the right hand side, the expression in the braces is exactly $\Delta^{Long}\left[\lambda_I,\{\lambda_l^{(end)}\},\lambda_D,f_D(.)\right]$ in Eq.(SA-3.2e), and we also have $\sum_{\kappa=1}^{\kappa_{\max}}L(s^A[cz(\gamma_\kappa)])=L(s^A)$. Thus, the expression is further reduced to:

$$\sum_{\kappa=1}^{\kappa_{\max}}R^{ID}_{X(Mik)}(s^A[cz(\gamma_\kappa)];cz(\gamma_\kappa))=(\lambda_I+\lambda_D)L(s^A)+\Delta^{Long}\left[\lambda_I,\{\lambda_l^{(end)}\},\lambda_D,f_D(.)\right]=R_X^{ID}(s^A,t)$$

--- Eq.(SA-3.11')

for $\kappa_{\max}>1$. [NOTE: In the case where $\kappa_{\max}=1$, by the way, arguments similar to those leading to Eqs.(SA-3.4b,c') reveals that $R^{ID}_{X(Mik)}(s^A[cz(\gamma_1)];cz(\gamma_1))=R_X^{ID}(s^A,t)$ holds, and thus that Eq.(SA-3.11') trivially holds.] Now, substituting Eq.(SA-3.11') back into Eq.(SA-3.10') while taking account of the time-independence of $R_X^{ID}(s^A,t)$ under this model, we finally get:

$$P_{Mik}\left[\left(\left[\vec{\hat{M}}\right]_{LHS},[t_I,t_F]\right)\middle|\,(s^A,t_I)\right]=\exp\left\{-\int_{t_I}^{t_F}d\tau\; R_X^{ID}(s^A,\tau)\right\}\prod_{\kappa=1}^{\kappa_{\max}}\mu_P\left[\left(\vec{\hat{M}}[\gamma_\kappa],[t_I,t_F]\right)\middle|\,(s^A,t_I)\right].$$

--- Eq.(SA-3.10")

The right hand side of Eq.(SA-3.10") is exactly that of Eq.(SM-2.12) (in Additional file 1) multiplied by $P\left[\left([\,],[t_I,t_F]\right)\middle|\,(s^A,t_I)\right]=\exp\left\{-\int_{t_I}^{t_F}d\tau\; R_X^{ID}(s^A,\tau)\right\}$. It is nothing other than the total probability of the LHS equivalence class $\left[\vec{\hat{M}}\right]_{LHS}$ calculated via our *ab initio* theoretical formulation, under the "long indel" model, Eqs.(SA-3.1a-d) (and Eqs.(SA-3.2a-e))(2.4.7a-e).

Actually, this equivalence between the probability via our *ab initio* formulation and that via Miklos et al.'s method [21] should hold under any indel models with factorable PWA probabilities described in Section R8 of the main Results and discussion, as long as the "chop-zones" are re-defined appropriately. Its explicit proof will be left as an exercise for the readers. (The key should be the decomposition of the entire exit rate into the contributions from (modified) chop-zones.)
